# The global burden of non-typhoidal salmonella invasive disease: a systematic analysis for the Global Burden of Disease Study 2017

**DOI:** 10.1016/S1473-3099(19)30418-9

**Published:** 2019-12

**Authors:** GBD 2017 Non-Typhoidal Salmonella Invasive Disease Collaborators, Jeffrey D. Stanaway, Andrea Parisi, Kaushik Sarkar, Brigette F. Blacker, Robert C Reiner, Simon I. Hay, Molly R Nixon, Christiane Dolecek, Spencer L. James, Ali H Mokdad, Getaneh Abebe, Elham Ahmadian, Fares Alahdab, Birhan Tamene T Alemnew, Vahid Alipour, Fatemeh Allah Bakeshei, Megbaru Debalkie Animut, Fereshteh Ansari, Jalal Arabloo, Ephrem Tsegay Asfaw, Mojtaba Bagherzadeh, Quique Bassat, Yaschilal Muche Muche Belayneh, Félix Carvalho, Ahmad Daryani, Feleke Mekonnen Demeke, Asmamaw Bizuneh Bizuneh Demis, Manisha Dubey, Eyasu Ejeta Duken, Susanna J Dunachie, Aziz Eftekhari, Eduarda Fernandes, Reza Fouladi Fard, Getnet Azeze Gedefaw, Birhanu Geta, Katherine B Gibney, Amir Hasanzadeh, Chi Linh Hoang, Amir Kasaeian, Amir Khater, Zelalem Teklemariam Kidanemariam, Ayenew Molla Lakew, Reza Malekzadeh, Addisu Melese, Desalegn Tadese Mengistu, Tomislav Mestrovic, Bartosz Miazgowski, Karzan Abdulmuhsin Mohammad, Mahdi Mohammadian, Abdollah Mohammadian-Hafshejani, Cuong Tat Nguyen, Long Hoang Nguyen, Son Hoang Nguyen, Yirga Legesse Nirayo, Andrew T Olagunju, Tinuke O Olagunju, Hadi Pourjafar, Mostafa Qorbani, Mohammad Rabiee, Navid Rabiee, Anwar Rafay, Aziz Rezapour, Abdallah M. Samy, Sadaf G. Sepanlou, Masood Ali Shaikh, Mehdi Sharif, Mika Shigematsu, Belay Tessema, Bach Xuan Tran, Irfan Ullah, Ebrahim M Yimer, Zoubida Zaidi, Christopher J L Murray, John A Crump

## Abstract

**Background:**

Non-typhoidal salmonella invasive disease is a major cause of global morbidity and mortality. Malnourished children, those with recent malaria or sickle-cell anaemia, and adults with HIV infection are at particularly high risk of disease. We sought to estimate the burden of disease attributable to non-typhoidal salmonella invasive disease for the Global Burden of Diseases, Injuries, and Risk Factors Study (GBD) 2017.

**Methods:**

We did a systematic review of scientific databases and grey literature, and estimated non-typhoidal salmonella invasive disease incidence and mortality for the years 1990 to 2017, by age, sex, and geographical location using DisMod-MR, a Bayesian meta-regression tool. We estimated case fatality by age, HIV status, and sociodemographic development. We also calculated the HIV-attributable fraction and estimated health gap metrics, including disability-adjusted life-years (DALYs).

**Findings:**

We estimated that 535 000 (95% uncertainty interval 409 000–705 000) cases of non-typhoidal salmonella invasive disease occurred in 2017, with the highest incidence in sub-Saharan Africa (34·5 [26·6–45·0] cases per 100 000 person-years) and in children younger than 5 years (34·3 [23·2–54·7] cases per 100 000 person-years). 77 500 (46 400–123 000) deaths were estimated in 2017, of which 18 400 (12 000–27 700) were attributable to HIV. The remaining 59 100 (33 300–98 100) deaths not attributable to HIV accounted for 4·26 million (2·38–7·38) DALYs in 2017. Mean all-age case fatality was 14·5% (9·2–21·1), with higher estimates among children younger than 5 years (13·5% [8·4–19·8]) and elderly people (51·2% [30·2–72·9] among those aged ≥70 years), people with HIV infection (41·8% [30·0–54·0]), and in areas of low sociodemographic development (eg, 15·8% [10·0–22·9] in sub-Saharan Africa).

**Interpretation:**

We present the first global estimates of non-typhoidal salmonella invasive disease that have been produced as part of GBD 2017. Given the high disease burden, particularly in children, elderly people, and people with HIV infection, investigating the sources and transmission pathways of non-typhoidal salmonella invasive disease is crucial to implement effective preventive and control measures.

**Funding:**

Bill & Melinda Gates Foundation.

## Introduction

Non-typhoidal salmonella infections most commonly result in self-limiting diarrhoeal illness with low case fatality. The Global Burden of Diseases, Injuries, and Risk Factors Study (GBD) 2017 estimated that salmonella enterocolitis resulted in 95·1 million cases (95% uncertainty interval [UI] 41·6–184·8), 50 771 deaths (2824–129 736), and 3·10 million DALYs (0·39–7·39) in 2017.^1^, ^2^, ^3^ In addition to diarrhoeal disease, non-typhoidal salmonella infections can invade normally sterile sites, resulting in bacteraemia, meningitis, and other focal infections. Referred to as invasive non-typhoidal salmonella disease, these infections are not typically associated with diarrhoea but present as non-specific febrile illnesses with symptoms that are clinically indistinguishable from other febrile illnesses, and with higher case fatality than is seen with non-invasive infection.^4^ Malnourished infants, elderly people, and individuals with sickle-cell disease, HIV, and acute or recent malaria are at particular risk.^5^, ^6^, ^7^, ^8^, ^9^, ^10^

Invasive disease is seen most commonly in sub-Saharan Africa, where certain non-typhoidal salmonella serovars and sequence types are endemic, including *Salmonella enterica* serovars Typhimurium sequence type (ST) 313, Enteritidis ST 11, Dublin, and Isangi.^8^, ^11^, ^12^, ^13^ A recently emerged highly invasive *S* Typhimurium strain, ST 313, has caused large epidemics of non-typhoidal salmonella bacteraemia showing resistance to multiple antimicrobial agents, including those recommended as first-line treatment.^12^ Similarly, a new variant of multidrug-resistant *S* Typhimurium, ST 34, has been associated with invasive disease in immunocompromised patients in Vietnam.^14^ Antimicrobial-resistant infections are associated with poorer clinical outcomes and higher case fatality.^15^ Studies evaluating the proportion of total isolates that are invasive, referred to as the invasiveness index, have identified *Salmonella enterica* serovars Dublin, Choleraesuis, Heidelberg, and Virchow as the most invasive.^10^, ^16^, ^17^ Conversely, *Salmonella enterica* serovar Newport has been associated with a lower risk of bacteraemia than *S* Typhimurium.^16^

**Research in context**

**Evidence before this study**

We searched PubMed, Web of Science, Scopus, and the grey literature using the search terms “(*Salmonella* OR salmonellosis) AND (nontyphoidal) AND (bacteraemia OR septicaemia OR bloodstream OR invasive) AND (incidence OR prevalence OR burden OR proportion OR fatality OR risk)” between Jan 1, 1980, and July 19, 2017, with no language restrictions. We identified two studies reporting the global non-typhoidal salmonella invasive disease burden. Both studies estimated the incidence of non-typhoidal salmonella invasive disease based on data from ten studies, of which seven were done in Africa. One estimated 597 000 cases and 63 300 deaths from non-typhoidal salmonella invasive disease in 2010; the other estimated 3·4 million cases and 681 000 deaths per year. No study has, to the best of our knowledge, estimated disease trends, modelled case fatality, or calculated the attributable fraction of risk factors, including co-infection with HIV.

**Added value of this study**

We present estimates of incidence, mortality, and case fatality for non-typhoidal salmonella invasive disease by year, age, sex, and country using data from 66 studies. We also calculated the HIV-attributable fraction and health gap metrics, including years of life lost (YLLs) to premature mortality, years lived with disability (YLDs), and disability-adjusted life-years (DALYs), as part of the Global Burden of Diseases, Injuries, and Risk Factors Study (GBD) 2017. Using a broadly scoped systematic review allowed us to identify many studies that were not included in the previous two estimates of non-typhoidal salmonella invasive disease burden, and using the DisMod modelling tool allowed us to integrate data with GBD and include other significant covariates in our model. This systematic analysis is, to the best of our knowledge, the first study presenting estimates of non-typhoidal salmonella invasive disease temporal trends, HIV-attributable fraction, and case fatality derived analytically at country, regional, and global levels. Finally, our estimates allow non-typhoidal salmonella invasive disease to be placed within the general context of the global burden of other diseases as presented in GBD 2017.

**Implications of all the available evidence**

Although the available evidence on non-typhoidal salmonella invasive disease is scarce, particularly in Latin America, we identified a high disease burden of non-typhoidal salmonella invasive disease in children, elderly people, and people with HIV infection, and in areas of low socioeconomic development. Non-typhoidal salmonella invasive disease is not as common as salmonella enterocolitis, but it results in similar numbers of deaths and DALYs. Therefore, investigating sources and transmission pathways of non-typhoidal salmonella invasive disease is crucial to implement effective preventive and control measures and conduct more studies from other geographical locations. Lastly, additional studies on other risk factors, including malaria, acute malnutrition, and sickle cell disease, are needed to calculate the attributable fraction of these risk factors to the non-typhoidal salmonella invasive disease burden.

Two previous studies have published estimates of the global burden of non-typhoidal salmonella invasive disease. Ao and colleagues^5^ used an HIV and malaria burden matrix extrapolated to countries without data and estimated that 3·4 million cases of non-typhoidal salmonella invasive disease and 681 316 deaths from non-typhoidal salmonella invasive disease occurred in 2010. WHO estimated that 596 824 cases of non-typhoidal salmonella invasive disease occurred in 2010, resulting in 63 312 deaths—considerably lower estimates than in the previous study.^5^ Both estimates were based on a small number of studies primarily from sub-Saharan Africa, and neither study estimated disease trends or modelled case fatality.

We present estimates of the non-typhoidal salmonella invasive disease burden from GBD 2017. GBD 2017 is the first iteration of GBD to include non-typhoidal salmonella invasive disease in its cause list. An updated and broadly scoped systematic review and improved estimation methods have allowed us to address some of the shortcomings of previously published estimates and provide a more complete picture of the non-typhoidal salmonella invasive disease burden. We present estimates of non-typhoidal salmonella invasive disease incidence and mortality by year, age, sex, and location. Additionally, we modelled non-typhoidal salmonella invasive disease case fatality and the proportion of cases attributable to HIV, and estimated health gap metrics, including years of life lost (YLLs) to premature mortality, years lived with disability (YLDs), and disability-adjusted life-years (DALYs).

## Methods

### Systematic review

We searched scientific databases, including PubMed, Web of Science, and Scopus, grey literature databases including Open Grey and Dart Europe, and other grey literature sources including websites of WHO, the European Food and Safety Authority, Health Canada, and the US Centers for Disease Control and Prevention for studies published between Jan 1, 1980, and July 19, 2017, that reported data on non-typhoidal salmonella invasive disease incidence, case fatality, or HIV co-infection. We defined a case of non-typhoidal salmonella invasive disease as culture-confirmed non-typhoidal salmonella infection of a normally sterile site (eg, blood). We excluded editorial letters, case studies, and reviews. No language restrictions were applied. Studies were independently screened by two authors (AP and KS), with any discrepancies discussed with a third author (JDS). Backward citation screening of eligible studies was also done. Search strategies and inclusion and exclusion criteria are detailed in the appendix (pp 3–6).

The review protocol is available in PROSPERO, CRD42017077141.^18^ The complete methodology is presented in the appendix (pp 3–6).

### Incidence estimation

We used data from cohort studies, clinical trials, and case notification systems to estimate non-typhoidal salmonella invasive disease incidence. Clinic-based and hospital-based studies often applied adjustments to correct for incomplete case capture. We extracted estimates that made adjustments for the catchment population and incomplete enrolment of eligible patients. Given the severity of non-typhoidal salmonella invasive disease, we did not include adjustments for health-seeking behaviour, which are generally based on responses in household surveys to questions about health-seeking behaviour for less severe, uncomplicated fever.

We modelled the incidence of non-typhoidal salmonella invasive disease by location, year, age, and sex by use of DisMod-MR, a Bayesian meta-regression tool that has been described elsewhere.^3^ Briefly, DisMod is a non-linear mixed-effects model that uses a Bayesian cascading geographical hierarchy in which all data are pooled to estimate a global fit, which then acts as a prior for each of the seven GBD super-regions (appendix p 2). The model includes hierarchical nested random effects at every level of the geographical hierarchy, and fixed effects for covariates. DisMod uses age integration to allow use of data with non-standardised age categorisation schemes and estimate location-specific age patterns.

We included three predictive covariates in our incidence model: HIV mortality rate, malaria incidence adjusted for antimalarial coverage and drug effectiveness, and an index of unsafe water exposure. All covariates were derived from GBD estimates.^1^, ^19^, ^20^ HIV and malaria were included as they are known risk factors for non-typhoidal salmonella invasive disease and have been previously shown to be strong predictors of non-typhoidal salmonella invasive disease incidence,^5^ and we included exposure to unsafe water supply types as a proxy for potential exposure to the underlying pathogen. We quantified unsafe water exposure using the summary exposure value, a metric of the risk-weighted prevalence of exposure to unimproved water, weighted by the type of water supply.^19^

### Case fatality

We estimated case fatality by age, HIV status, and socioeconomic development. Not all studies reported case fatality separately for non-typhoidal salmonella invasive disease with and without HIV infection. Where studies reported case fatality separately for HIV status, we included those as separate data points for which we assigned each point an HIV prevalence of 1 or 0. For studies that reported overall case fatality, we included a single case fatality data point with the HIV prevalence corresponding to the reported prevalence of HIV among non-typhoidal salmonella invasive disease cases in that study. We quantified development using the Socio-demographic Index (SDI), a measure of development level based on the total fertility rate among women younger than 25 years of age, mean education for those aged 15 years and older, and lag-distributed income per capita.^19^ Finally, we used a generalised additive model with linear terms on HIV prevalence and SDI, and P-splines on age to allow for a flexible, non-linear age pattern.

### HIV attribution

For each location, year, age, and sex, we estimated the proportion of cases attributable to HIV based on the relative risk (RR) of non-typhoidal salmonella invasive disease, comparing those with HIV to those without HIV. We used negative binomial regression to model RRs as a function of age and the summary exposure value for diarrhoea risk:

$$In(RR_{HIV})=\propto +\beta_{1}\times \ln(age)+\beta_{2}\times \ln(SEV_{diarrhoea})$$

The diarrhoea summary exposure value is an index of exposure to risk factors for diarrhoea that is estimated in the GBD risk factor analysis and has been detailed elsewhere.^19^ These variables were included as we expect them to be strong predictors of the underlying risk of non-typhoidal salmonella invasive disease in the absence of HIV, and should thus strongly predict the baseline risk of non-typhoidal salmonella invasive disease (ie, the risk among the unexposed). We estimated the proportion of non-typhoidal salmonella invasive disease cases attributable to HIV as the population attributable fraction (PAF), calculated as

$$PAF=\frac{P_{hiv}\times (RR-1)}{P_{hiv}\times (RR-1)+1}$$

where *P*_hiv_ is the prevalence of HIV in the population. The PAF does not equal the prevalence of HIV among those with non-typhoidal salmonella invasive disease; rather, it equals the proportion of non-typhoidal salmonella invasive disease cases that are attributable to HIV infection. This approach assumes that some portion of non-typhoidal salmonella invasive disease cases among those with HIV were not a direct result of the HIV infection.

We estimated the incidence of non-typhoidal salmonella invasive disease attributable to HIV for each location, year, age, and sex as the product of the total incidence of non-typhoidal salmonella invasive disease and the HIV PAF, and the incidence of non-typhoidal salmonella invasive disease not attributable to HIV for each location, year, age, and sex as the product of the total incidence of non-typhoidal salmonella invasive disease and one minus the HIV PAF.

### Mortality estimation

We estimated HIV-attributable non-typhoidal salmonella invasive disease mortality rates as the product of the HIV-attributable non-typhoidal salmonella invasive disease incidence and the corresponding estimate of non-typhoidal salmonella invasive disease case fatality among those living with HIV, and non-HIV-attributable non-typhoidal salmonella invasive disease mortality rates as the product of the non-HIV-attributable non-typhoidal salmonella invasive disease incidence and the corresponding estimate of non-typhoidal salmonella invasive disease case fatality among those living without HIV (appendix p 8).

A fundamental principle of the GBD approach is that the list of causes of death is considered to be mutually exclusive and collectively exhaustive such that each death must be assigned to a single cause and the sum of all cause-specific mortality estimates must equal all-cause mortality within each age, sex, year, and location. Where a death might be considered to result from multiple causes, we assigned the death to the underlying cause. We therefore considered the underlying cause of death to be HIV for all non-typhoidal salmonella invasive disease deaths that were attributable to HIV. Although we estimated total non-typhoidal salmonella invasive disease mortality, the final GBD mortality estimates include only deaths from non-typhoidal salmonella invasive disease not attributable to HIV. Finally, we imposed consistency between cause-specific and all-cause mortality estimates across all causes in GBD through CoDCorrect, a process in which we rescaled cause-specific mortality estimates to fit the all-cause mortality envelope.^1^

### Health gap metric estimation

We estimated three health gap metrics: YLLs, YLDs, and DALYs. The number of YLLs resulting from a given death is equal to the life expectancy at the age of death, based on a reference life table, and the total number of YLLs for a given location and year equal the sum of all age-specific YLLs. YLDs are calculated as the product of prevalence and a disability weight that quantifies the severity of the outcome. DALYs are calculated as the sum of YLLs and YLDs.

We calculated prevalence as the product of incidence and duration, doing all calculations at the draw level to incorporate uncertainty in both incidence and duration estimates. Given the broad but almost universally severe clinical presentations of non-typhoidal salmonella invasive disease, we applied the health state “severe acute infectious disease episode” to all cases of non-typhoidal salmonella invasive disease, with a corresponding disability weight of 0·133 (95% UI 0·088–0·190). We assumed that 95% of causes would result in symptoms lasting between 7 and 21 days.^21^ We estimated YLDs as the product of this disability weight and prevalence. Finally, we adjusted YLDs through a comorbidity correction to ensure that comorbidities cannot result in an aggregate disability weight greater than one (equivalent to death).

Data management was done with Stata, version 13, and R, version 3.4.3. We estimated RRs for HIV using Stata version 13, and case fatality using the mgcv package in R, version 1.8.23.

### Role of the funding source

The sponsor of the study had no role in study design, data collection, data analysis, data interpretation, or writing of the report. The corresponding author had full access to all the data in the study and had final responsibility for the decision to submit for publication.

## Results

After removing 2086 duplicate records, we screened 2480 publications by title and 588 by abstract, and 408 underwent full-text screening. 66 studies met our inclusion criteria: 35 reported data on incidence, 35 on case fatality rate, and six on co-infections with HIV (appendix pp 7, 52–56).

**Figure 1:**
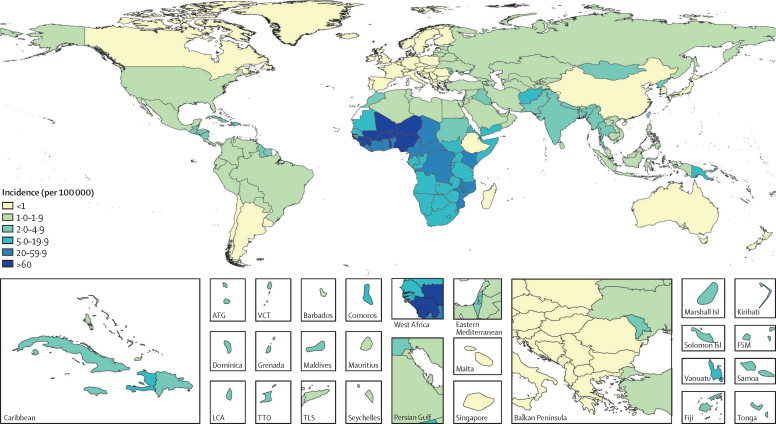
Non-typhoidal salmonella invasive disease incidence rates (per 100 000), by country, in 2017 Locations filled in white are those for which the Global Burden of Disease Study (GBD) does not produce estimates. The inset maps detail smaller locations. ATG=Antigua and Barbuda. VCT=Saint Vincent and the Grenadines. Isl=Islands. FSM=Federated States of Micronesia. LCA=Saint Lucia. TTO=Trinidad and Tobago. TLS=Timor-Leste.

An estimated 342 000 (95% UI 265 000–451 000) cases of non-typhoidal salmonella invasive disease occurred globally in 1990, increasing to 691 000 (552 000–874 000) cases in 2005 and declining to 535 000 (409 000–705 000) cases in 2017 (table 1). Age-standardised incidence showed a similar trend, with 5·9 (4·6–7·7) cases per 100 000 person-years in 1990, increasing to 10·7 (8·5–13·6) cases per 100 000 person-years in 2005 and decreasing to 7·5 (5·7–10·0) cases per 100 000 person-years in 2017. For all years, we observed the largest numbers of cases and highest incidence in sub-Saharan Africa (figure 1; appendix pp 15, 24): in 2017 the age-standardised incidence of non-typhoidal salmonella invasive disease in sub-Saharan Africa was 34·5 (26·6–45·0) cases per 100 000 person-years, and this super-region accounted for 78·8% (74·6–82·9) of all cases globally.

**Table 1:** Cases and incidence of non-typhoidal salmonella invasive disease, and the proportion of cases attributable to HIV, by year, super-region, age, and sex

	**Cases (thousands)**	**Incidence (per 100 000)**	**Proportion of cases attributable to HIV**
**Year**			
1990	342·0 (264·8 to 451·2)	5·9 (4·6 to 7·7)	5·2 (2·6 to 8·5)
1995	410·5 (319·7 to 535·9)	6·8 (5·3 to 8·8)	8·9 (4·8 to 14·2)
2000	521·2 (410·4 to 669·4)	8·3 (6·6 to 10·7)	11·0 (6·0 to 17·5)
2005	690·7 (552·2 to 873·6)	10·7 (8·5 to 13·6)	10·4 (5·5 to 16·9)
2010	622·0 (490·1 to 800·0)	9·2 (7·3 to 12·0)	8·5 (4·3 to 14·0)
2017	534·6 (409·0 to 705·0)	7·5 (5·7 to 10·0)	8·2 (4·4 to 13·2)
**GBD super-region**			
Southeast Asia, east Asia, and Oceania	21·5 (15·7 to 28·3)	1·2 (0·9 to 1·6)	2·0 (1·1 to 3·3)
Central Europe, eastern Europe, and central Asia	4·8 (3·6 to 6·1)	1·3 (1·0 to 1·7)	5·3 (3·2 to 8·2)
High-income	10·9 (8·1 to 13·7)	1·1 (0·8 to 1·4)	19·1 (9·0 to 32·6)
Latin America and Caribbean	11·2 (8·4 to 14·4)	2·0 (1·5 to 2·6)	5·5 (3·1 to 8·9)
North Africa and Middle East	15·8 (12·3 to 20·3)	2·5 (2·0 to 3·2)	0·5 (0·2 to 0·9)
South Asia	48·9 (35·8 to 64·6)	2·7 (2·0 to 3·5)	0·9 (0·5 to 1·7)
Sub-Saharan Africa	421·6 (316·0 to 574·1)	34·5 (26·6 to 45·0)	9·5 (5·0 to 15·7)
**Age group**			
<5 years	233·4 (158·2 to 372·4)	34·3 (23·2 to 54·7)	0·5 (0·0 to 1·3)
5–14 years	120·8 (68·7 to 183·2)	9·3 (5·3 to 14·1)	1·6 (0·7 to 3·1)
15–49 years	146·9 (107·4 to 201·2)	3·8 (2·7 to 5·1)	22·3 (13·7 to 32·7)
50–69 years	26·8 (16·6 to 39·6)	2·0 (1·3 to 3·0)	25·4 (13·9 to 38·4)
≥70 years	6·6 (4·5 to 9·8)	1·5 (1·0 to 2·3)	15·5 (7·2 to 26·6)
**Sex**			
Male	278·2 (212·8 to 370·3)	7·7 (5·8 to 10·3)	7·6 (4·0 to 12·4)
Female	256·4 (196·1 to 340·8)	7·4 (5·6 to 9·9)	8·9 (4·9 to 14·3)

95% uncertainty intervals are included in parentheses. Year-specific, age-specific, and sex-specific estimates are global estimates for 2017. Rates are age standardised, except for age-specific estimates. GBD=Global Burden of Disease.

**Figure 2:**
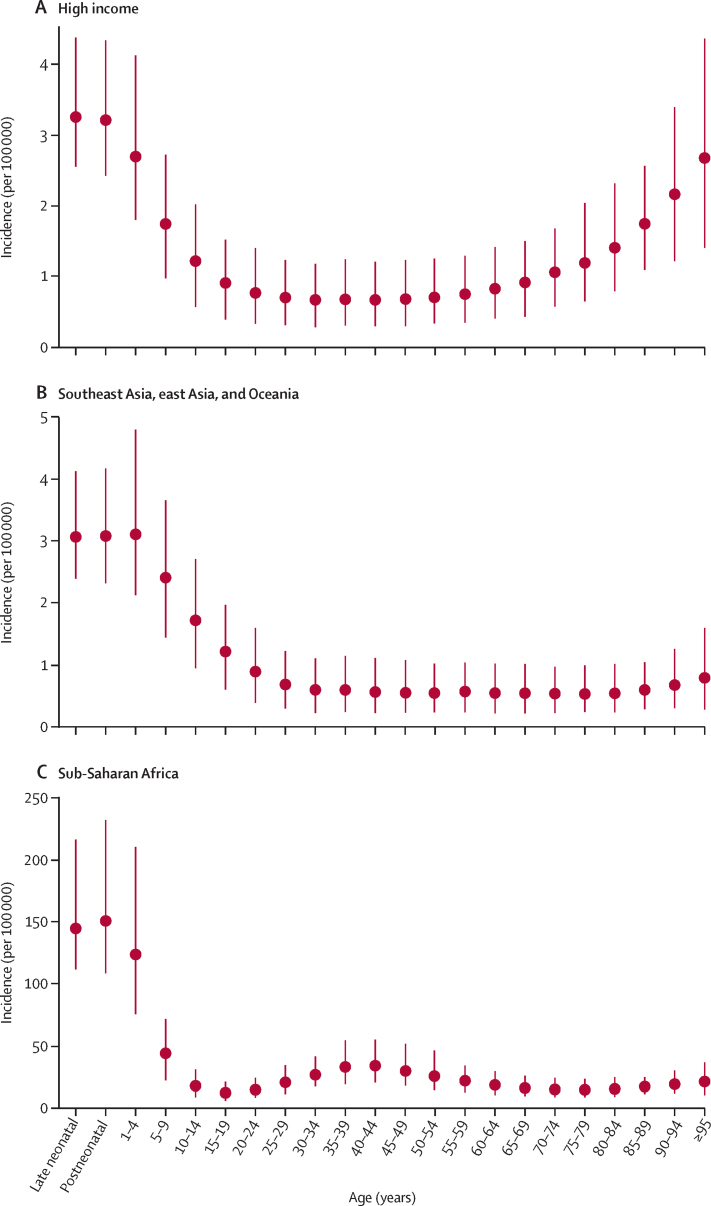
Incidence by age in 2017, in three super-regions (A) High-income. (B) Southeast Asia, east Asia, and Oceania. (C) Sub-Saharan Africa. 95% uncertainty intervals are shown.

Globally, we observed the highest incidence of non-typhoidal salmonella invasive disease among children younger than 5 years, with substantially declining incidence between ages 5 years and 15 years, followed by low incidence among those aged 15 years and older, and slight increases around age 35 years and with increasing age among those older than 85 years (appendix p 11). Slightly different patterns emerged within different epidemiological contexts. In the high-income super-region, the age pattern was U-shaped, with the highest incidence among young and elderly people (figure 2A); in low-income and middle-income regions outside of sub-Saharan Africa, we saw the highest rates among children, with lower rates throughout adulthood (figure 2B); and in sub-Saharan Africa we saw the highest rates among children, but also an approximately two-fold to three-fold increase between age 15–19 years and age 35–49 years, reflecting the effect of the region's high HIV prevalence on the risk of non-typhoidal salmonella invasive disease (figure 2C). Incidence was not significantly different for males and females; the 95% UIs for each sex include the point estimate for the other sex (table 1).

**Figure 3:**
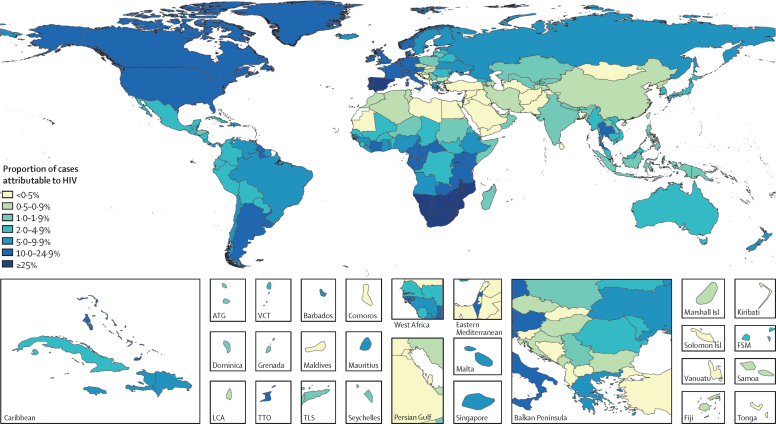
The percentage of non-typhoidal salmonella invasive disease cases that were attributable to HIV in 2017, by country Unfilled locations are those for which the Global Burden of Disease Study (GBD) does not produce estimates. The inset maps detail smaller locations. ATG=Antigua and Barbuda. VCT=Saint Vincent and the Grenadines. Isl=Islands. FSM=Federated States of Micronesia. LCA=Saint Lucia. TTO=Trinidad and Tobago. TLS=Timor-Leste.

Estimates of the RR of non-typhoidal salmonella invasive disease associated with HIV infection were higher with older age and with lower levels of diarrhoea risk exposure (appendix p 13). In 2017, RR estimates ranged from 2·89 (95% UI 1·14–7·32) among those younger than 1 year of age in sub-Saharan Africa to 232·64 (76·81–704·57) among those aged 90–95 years in the high-income super-region. Globally, we estimated that 5·2% (2·6–8·5) of non-typhoidal salmonella invasive disease cases were attributable to HIV in 1990, increasing to 11·0% (6·0–17·5) in 2000, and declining to 8·2% (4·4–13·2) in 2017 (table 1). Our model suggests that the proportion of cases attributable to HIV was highest in countries with higher HIV risk and lower diarrhoea risk (figure 3).

Mean all-age case fatality was 14·5% (95% UI 9·2–21·1) in 2017, with higher estimates among children younger than 5 years (13·5% [8·4–19·8]) and elderly people (51·2% [30·2–72·9] among those aged ≥70 years), people with HIV infection (41·8% [30·0–54·0]), and in areas of low sociodemographic development (eg, 15·8% [10·0–22·9] in sub-Saharan Africa; appendix pp 12, 13). In 2017, mean case fatality among those living with HIV was 41·8% (30·0–54·0), compared with 12·0% (7·3–18·0) among those without HIV. At the global level, estimates of non-typhoidal salmonella invasive disease case fatality in 2017 ranged from a low of 8·8% (5·3–13·4) among those aged 5–14 years without HIV, to a high of 75·7% (58·0–88·1) among those aged 70 years and older with HIV (appendix p 12).

**Figure 4:**
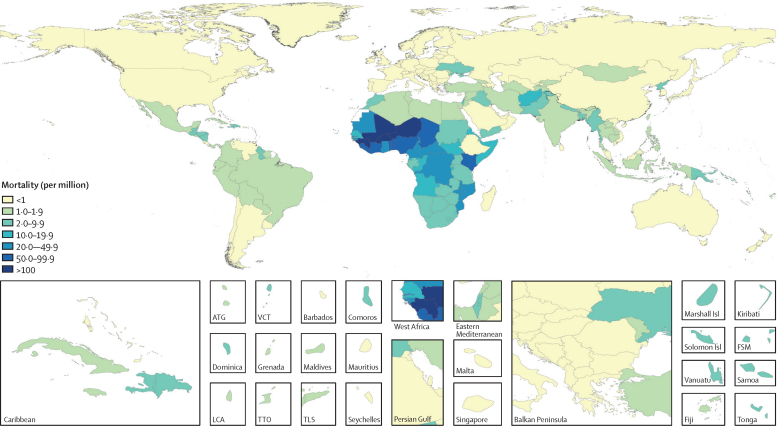
Non-typhoidal salmonella invasive disease mortality rates (per million), by country, in 2017 Unfilled locations are those for which the Global Burden of Disease Study (GBD) does not produce estimates. The inset maps detail smaller locations. ATG=Antigua and Barbuda. VCT=Saint Vincent and the Grenadines. Isl=Islands. FSM=Federated States of Micronesia. LCA=Saint Lucia. TTO=Trinidad and Tobago. TLS=Timor-Leste.

We estimated that 51 800 (95% UI 29 800–83 900) deaths from non-typhoidal salmonella invasive disease occurred globally in 1990, increasing to 108 000 (66 600–164 000) in 2005, and declining to 77 500 (46 400–123 000) in 2017 (table 2). Age-standardised mortality rates showed a similar trend, with 9·4 (5·4–15·0) deaths per 100 000 person-years in 1990, increasing to 16·8 (10·4–25·5) per 100 000 person-years in 2005, and decreasing to 10·7 (6·4–17·0) per 100 000 person-years in 2017. Of these, 17·9% (12·3–23·9) were attributable to HIV in 1990, as were 31·1% (23·3–40·1) in 2005, and 24·3% (16·9–32·9) in 2017. After removing deaths from non-typhoidal salmonella invasive disease for which HIV was considered the underlying cause of death, final GBD estimates for non-typhoidal salmonella invasive disease mortality were 42 800 (23 600–72 700) deaths in 1990, 74 800 (43 300–120 000) deaths in 2005, and 59 100 (33 300–98 100) deaths in 2017. The spatial distribution of mortality rates is similar to that for incidence rates (figure 4).

**Table 2:** Deaths and mortality rates for non-typhoidal salmonella invasive disease, among those living with and without HIV, and the total population, by year, super-region, age, and sex

	**Deaths and mortality rates in people without HIV infection**		**Deaths and mortality rates in people with HIV infection**		**Total deaths and mortality rates**		**Proportion of cases attributable to HIV (%)**
	Deaths (thousands)	Mortality rate (per million)	Deaths (thousands)	Mortality rate (per million)	Deaths (thousands)	Mortality rate (per million)	
**Year**							
1990	42·78 (23·62 to 72·70)	7·60 (4·27 to 12·55)	9·02 (5·96 to 13·22)	1·78 (1·18 to 2·59)	51·81 (29·75 to 83·92)	9·38 (5·44 to 15·02)	17·9% (12·3 to 23·9)
1995	48·58 (27·01 to 80·99)	8·27 (4·64 to 13·63)	17·87 (12·17 to 25·81)	3·19 (2·17 to 4·59)	66·45 (39·59 to 104·91)	11·46 (6·91 to 17·83)	27·5% (20·1 to 35·5)
2000	59·06 (32·68 to 97·53)	9·71 (5·38 to 15·94)	27·48 (18·65 to 40·13)	4·48 (3·05 to 6·49)	86·54 (52·48 to 133·64)	14·19 (8·63 to 21·85)	32·3% (24·4 to 41·3)
2005	74·81 (43·33 to 120·08)	11·81 (6·87 to 19·00)	33·13 (21·99 to 49·05)	4·98 (3·33 to 7·35)	107·93 (66·61 to 163·87)	16·79 (10·36 to 25·49)	31·1% (23·3 to 40·1)
2010	67·56 (39·19 to 110·02)	10·14 (5·84 to 16·53)	23·36 (15·30 to 35·10)	3·25 (2·14 to 4·88)	90·92 (55·54 to 140·01)	13·39 (8·21 to 20·70)	26·2% (19·1 to 34·2)
2017	59·07 (33·32 to 98·06)	8·37 (4·73 to 13·99)	18·40 (12·01 to 27·68)	2·31 (1·50 to 3·45)	77·47 (46·36 to 122·80)	10·68 (6·42 to 17·02)	24·3% (16·9 to 32·9)
**GBD super-region**							
Southeast Asia, east Asia, and Oceania	1·60 (0·86 to 2·68)	0·83 (0·45 to 1·38)	0·16 (0·09 to 0·24)	0·06 (0·04 to 0·10)	1·75 (0·96 to 2·88)	0·89 (0·48 to 1·47)	9·2% (6·5 to 12·0)
Central Europe, eastern Europe, and central Asia	0·38 (0·20 to 0·66)	0·89 (0·47 to 1·53)	0·06 (0·03 to 0·11)	0·13 (0·07 to 0·22)	0·45 (0·24 to 0·76)	1·02 (0·54 to 1·76)	14·4% (9·7 to 19·7)
High-income	0·70 (0·32 to 1·29)	0·50 (0·24 to 0·89)	0·68 (0·38 to 1·03)	0·41 (0·22 to 0·63)	1·38 (0·72 to 2·25)	0·91 (0·49 to 1·51)	50·0% (39·4 to 59·0)
Latin America and Caribbean	0·81 (0·45 to 1·34)	1·46 (0·81 to 2·40)	0·23 (0·14 to 0·34)	0·38 (0·22 to 0·56)	1·04 (0·59 to 1·68)	1·84 (1·04 to 2·99)	22·3% (16·9 to 28·3)
North Africa and Middle East	1·63 (0·91 to 2·66)	2·76 (1·56 to 4·45)	0·03 (0·01 to 0·05)	0·05 (0·03 to 0·08)	1·66 (0·93 to 2·70)	2·81 (1·60 to 4·52)	1·7% (1·0 to 2·7)
South Asia	4·50 (2·47 to 7·45)	2·60 (1·43 to 4·25)	0·17 (0·10 to 0·26)	0·10 (0·06 to 0·15)	4·67 (2·55 to 7·72)	2·69 (1·48 to 4·42)	3·6% (2·4 to 5·1)
Sub-Saharan Africa	49·44 (27·57 to 83·50)	43·11 (24·33 to 70·34)	17·08 (11·05 to 25·73)	23·66 (15·48 to 35·37)	66·52 (40·13 to 105·50)	66·78 (41·72 to 99·09)	26·2% (18·0 to 35·7)
**Age group**							
<5 years	31·20 (16·20 to 56·59)	45·84 (23·80 to 83·14)	0·43 (0·21 to 0·85)	0·64 (0·31 to 1·24)	31·63 (16·40 to 57·49)	46·48 (24·09 to 84·47)	1·4% (0·8 to 2·3)
5–14 years	10·40 (5·16 to 18·72)	8·02 (3·98 to 14·43)	0·76 (0·36 to 1·36)	0·58 (0·27 to 1·05)	11·16 (5·53 to 19·90)	8·60 (4·27 to 15·34)	6·9% (5·0 to 9·2)
15–49 years	10·03 (4·73 to 18·02)	2·56 (1·21 to 4·61)	12·49 (7·57 to 19·75)	3·19 (1·93 to 5·05)	22·52 (12·80 to 36·79)	5·76 (3·27 to 9·41)	56·0% (45·7 to 65·7)
50–69 years	4·92 (2·52 to 8·18)	3·73 (1·91 to 6·21)	3·84 (2·31 to 5·91)	2·92 (1·76 to 4·48)	8·76 (4·91 to 13·77)	6·65 (3·73 to 10·45)	44·3% (35·8 to 53·0)
≥70 years	2·52 (1·15 to 4·47)	5·82 (2·66 to 10·33)	0·88 (0·54 to 1·39)	2·03 (1·26 to 3·22)	3·40 (1·72 to 5·74)	7·85 (3·98 to 13·26)	26·7% (19·4 to 37·0)
**Sex**							
Male	30·97 (17·36 to 51·91)	8·62 (4·81 to 14·48)	9·00 (5·83 to 13·50)	2·27 (1·47 to 3·37)	39·97 (23·63 to 64·21)	10·88 (6·43 to 17·56)	23·0% (15·8 to 31·6)
Female	28·10 (16·01 to 46·61)	8·14 (4·62 to 13·65)	9·40 (6·09 to 14·32)	2·36 (1·53 to 3·60)	37·50 (22·89 to 58·79)	10·50 (6·34 to 16·54)	25·6% (18·0 to 34·4)

95% uncertainty intervals are included in parentheses. Year-specific, age-specific, and sex-specific estimates are global estimates for 2017. Rates are age standardised, except for age-specific estimates. GBD=Global Burden of Disease.

We estimated that non-typhoidal salmonella invasive disease was responsible for 1750 (95% UI 1000–2740) YLDs in 1990, 3490 (2060–5440) YLDs in 2005, and 2730 (1550–4290) YLDs in 2017. On average, each death from non-typhoidal salmonella invasive disease resulted in 72·0 (67·4–76·3) YLLs, resulting in 2·99 million (1·63–5·22) YLLs due to non-typhoidal salmonella invasive disease in 1990, 5·33 million (3·11–8·72) in 2005, and 4·26 million (2·38–7·38) in 2017. Non-typhoidal salmonella invasive disease was responsible for 3·00 million (1·63–5·22) DALYs in 1990, 5·33 million (3·11–8·73) in 2005, and 4·26 million (2·38–7·38) in 2017 (table 3).

**Table 3:** Years lived with disability, years of life lost, and disability-adjusted life-years from non-typhoidal salmonella invasive disease, by year, super-region, age, and sex

	**YLDs**		**YLLs**		**DALYs**	
	Number (thousands)	Rate (per millions)	Number (thousands)	Rate (per millions)	Number (thousands)	Rate (per millions)
**Year**						
1990	1·75 (1·00 to 2·74)	0·30 (0·17 to 0·47)	2993·9 (1626·4 to 5216·5)	500·6 (273·0 to 867·2)	2995·6 (1628·5 to 5218·6)	500·9 (273·3 to 867·7)
1995	2·09 (1·18 to 3·26)	0·35 (0·20 to 0·53)	3422·3 (1889·7 to 5936·7)	559·3 (310·0 to 964·8)	3424·4 (1892·1 to 5939·2)	559·6 (310·2 to 965·3)
2000	2·64 (1·51 to 4·07)	0·42 (0·24 to 0·65)	4200·6 (2321·8 to 7173·7)	676·0 (373·4 to 1152·3)	4203·2 (2324·4 to 7175·5)	676·4 (373·8 to 1152·6)
2005	3·49 (2·06 to 5·44)	0·54 (0·32 to 0·85)	5325·6 (3106·9 to 8722·7)	835·8 (487·5 to 1370·6)	5329·0 (3110·6 to 8726·4)	836·4 (488·1 to 1371·3)
2010	3·16 (1·81 to 5·03)	0·47 (0·27 to 0·75)	4847·6 (2743·8 to 8116·5)	731·8 (415·1 to 1234·0)	4850·7 (2745·7 to 8121·7)	732·3 (415·4 to 1234·6)
2017	2·73 (1·55 to 4·29)	0·38 (0·22 to 0·61)	4260·8 (2382·0 to 7378·6)	616·5 (346·8 to 1075·8)	4263·5 (2384·9 to 7382·0)	616·8 (347·3 to 1076·2)
**GBD super-region**						
Southeast Asia, east Asia, and Oceania	0·11 (0·06 to 0·17)	0·06 (0·04 to 0·10)	85·3 (47·1 to 145·7)	49·8 (27·4 to 87·5)	85·4 (47·2 to 145·7)	49·9 (27·4 to 87·6)
Central Europe, eastern Europe, and central Asia	0·02 (0·01 to 0·04)	0·07 (0·04 to 0·10)	17·0 (9·1 to 29·4)	49·1 (25·4 to 88·6)	17·0 (9·2 to 29·4)	49·2 (25·5 to 88·7)
High-income	0·06 (0·03 to 0·08)	0·06 (0·03 to 0·09)	22·1 (11·0 to 39·5)	24·9 (12·2 to 46·9)	22·2 (11·0 to 39·6)	25·0 (12·3 to 47·0)
Latin America and Caribbean	0·06 (0·03 to 0·09)	0·10 (0·06 to 0·16)	48·8 (27·2 to 82·5)	89·5 (50·5 to 150·4)	48·9 (27·3 to 82·6)	89·6 (50·6 to 150·6)
North Africa and Middle East	0·08 (0·05 to 0·13)	0·13 (0·08 to 0·20)	109·0 (61·2 to 181·5)	175·1 (98·4 to 289·7)	109·1 (61·2 to 181·6)	175·2 (98·5 to 289·8)
South Asia	0·25 (0·14 to 0·39)	0·14 (0·08 to 0·22)	286·2 (154·2 to 488·0)	157·0 (85·4 to 264·5)	286·5 (154·4 to 488·3)	157·2 (85·6 to 264·6)
Sub-Saharan Africa	2·15 (1·17 to 3·55)	1·76 (0·98 to 2·77)	3692·3 (2040·5 to 6427·8)	2685·9 (1493·7 to 4550·6)	3694·4 (2042·7 to 6430·4)	2687·7 (1495·4 to 4552·8)
**Age group**						
<5 years	1·19 (0·60 to 2·12)	1·74 (0·88 to 3·12)	2685·5 (1394·9 to 4872·0)	3945·6 (2049·3 to 7158·0)	2686·7 (1396·0 to 4873·0)	3947·3 (2051·0 to 7159·3)
5–14 years	0·62 (0·29 to 1·07)	0·48 (0·22 to 0·83)	823·7 (407·2 to 1485·1)	634·9 (313·9 to 1144·6)	824·4 (407·7 to 1486·2)	635·4 (314·2 to 1145·4)
15–49 years	0·75 (0·43 to 1·20)	0·19 (0·11 to 0·31)	573·0 (267·5 to 1037·9)	146·5 (68·4 to 265·3)	573·7 (268·1 to 1038·8)	146·7 (68·5 to 265·6)
50–69 years	0·14 (0·07 to 0·24)	0·10 (0·05 to 0·18)	144·5 (73·1 to 241·8)	109·6 (55·5 to 183·4)	144·6 (73·2 to 241·9)	109·7 (55·5 to 183·5)
≥70 years	0·03 (0·02 to 0·06)	0·08 (0·04 to 0·13)	34·1 (16·0 to 59·7)	78·7 (37·0 to 137·9)	34·1 (16·0 to 59·7)	78·8 (37·0 to 138·0)
**Sex**						
Male	1·42 (0·80 to 2·25)	0·39 (0·22 to 0·62)	2237·7 (1234·8 to 3852·1)	629·6 (348·8 to 1087·1)	2239·1 (1236·3 to 3854·1)	630·0 (349·3 to 1087·8)
Female	1·31 (0·75 to 2·08)	0·38 (0·21 to 0·60)	2,023·1 (1146·5 to 3499·5)	602·9 (339·8 to 1047·9)	2024·4 (1147·6 to 3500·6)	603·3 (340·0 to 1048·5)

95% uncertainty intervals are included in parentheses. Year-specific, age-specific, and sex-specific estimates are global estimates for 2017. Rates are age standardised, except for age-specific estimates. Estimates of YLLs and DALYs do not include burden for non-typhoidal salmonella invasive disease deaths attributable to HIV. YLD=years lived with disability. YLL=years of life lost. DALY=disability-adjusted life-year. GBD=Global Burden of Disease.

## Discussion

We estimate that 535 000 (95% UI 409 000–705 000) cases of non-typhoidal salmonella invasive disease occurred in 2017, which caused 77 500 (46 400–123 000) deaths (including those for which non-typhoidal salmonella invasive disease was attributable to HIV) and 4·26 million (2·38–7·38) DALYs. In the same year, an estimated 14·3 million (12·5–16·3) cases of typhoid and paratyphoid fever occurred, which led to 136 000 (76 900–219 000) deaths and 9·8 million (5·6–15·8) DALYs.^21^ So, although typhoid and paratyphoid fever were responsible for more than 25 times as many cases as non-typhoidal salmonella invasive disease, the greater severity and case fatality of non-typhoidal salmonella invasive disease resulted in almost half as many deaths and DALYs as did typhoid and paratyphoid fever. Similarly, an estimated 95·1 million (41·6–184·8) cases of salmonella enterocolitis occurred in 2017, which led to 50 800 (2820–130 000) deaths and 3·10 million (0·39–7·39) DALYs.^1^, ^2^, ^3^ Although salmonella enterocolitis occurs far more commonly than does non-typhoidal salmonella invasive disease, the number of deaths and DALYs caused by each disease is similar.

Two previous studies reported non-typhoidal salmonella invasive disease burden estimates, and both produced estimates for the year 2010.^5^, ^22^ Compared with our estimates for the same year, those published by WHO were slightly, but not substantially, lower. Whereas WHO estimated that non-typhoidal salmonella invasive disease caused 597 000 illnesses, 63 300 (95% UI 39 000–94 200) deaths, and 3·90 million (2·40–5·79) DALYs in 2010, we estimated 622 000 (490 000–800 000) cases, 67 600 (39 200–110 000) deaths, and 4·85 million (2·75–8·12) DALYs.^21^ Our estimates for 2010 are, however, significantly lower than those by Ao and colleagues, of 3·4 million non-typhoidal salmonella invasive disease cases and 681 000 deaths for the same year.^5^ As previously reported, the highest number of non-typhoidal salmonella invasive disease cases occurred in Africa and among children younger than 5 years.^5^, ^21^ Ao and colleagues estimated the case fatality rate to be 20%.^5^ The WHO study assumed that the case fatality rate for non-typhoidal salmonella invasive disease in individuals not infected with HIV ranged from 3·9% to 20%, with higher values in countries with higher overall child and adult mortality rates.^22^ In our study, mean all-age case fatality was 14·5% in 2017, with higher estimates among children, elderly people, and those with HIV, and in low SDI settings.

Our comprehensive systematic review allowed us to identify studies not included in previous estimates.^5^, ^22^ We present the first non-typhoidal salmonella invasive disease burden estimates to include temporal trends and HIV-attributable fraction, and the first analytically derived estimates of non-typhoidal salmonella invasive disease case fatality. The DisMod modelling tool allowed us to use data with disparate age classification schemes and estimate location-year-specific age patterns, and it allowed us to use information from predictive covariates while ensuring that our estimates maintained a strong spatial structure. Using posterior simulation allowed us to propagate uncertainty from all components through the modelling chain. Finally, integrating our model within the larger GBD framework allowed us to correct our mortality estimates to account for other causes of death, through CoDCorrect, and to adjust non-fatal burden for comorbidities, through comorbidity correction.

Still, data on non-typhoidal salmonella invasive disease remain scarce, and we identified few studies reporting incidence from Asia and no such studies from Latin America (appendix p 10). As studies were often done in high-risk communities, they might not be nationally representative, potentially yielding overestimates of non-typhoidal salmonella invasive disease burden. Conversely, clinic-based surveillance would fail to capture individuals with non-typhoidal salmonella invasive disease who did not seek care. Although non-typhoidal salmonella invasive disease is generally regarded as a severe illness that would result in care-seeking, relatively mild or moderate forms of the disease might exist. Alternatively, given its rapid onset and high case fatality, some deaths from non-typhoidal salmonella invasive disease might occur before patients are able to reach health-care facilities, particularly for those living in remote areas who have to travel long distances to reach such facilities. To the extent that either mild or rapidly fatal cases occur, some proportion of non-typhoidal salmonella invasive disease cases might not present to health-care facilities, which would result in our model underestimating burden.

Our estimates of HIV PAFs have notably wide uncertainty, which reflects the scarce data available to inform this model: we found only six studies from four countries reporting RRs (or data allowing calculation of RRs), comparing the risk of non-typhoidal salmonella invasive disease among those with HIV to the risk among those without HIV (appendix p 56). Although increased use of antiretroviral therapy (ART) has been associated with decreasing non-typhoidal salmonella invasive disease incidence,^23^ we found insufficient data to allow us to account for effective ART in our HIV-attributable estimates. Accounting for the possible role of ART is complicated by evidence suggesting that the risk of bloodstream infections, including non-typhoidal salmonella invasive disease, might dramatically increase during the first year after initiating ART, and then decline below pre-ART levels in subsequent years.^24^, ^25^ Our results might, therefore, underestimate HIV-attributable proportions in the years before widespread ART use, and overestimate those proportions in recent years.

Improved access to ART in the past decade has probably helped to reduce the burden of non-typhoidal salmonella invasive disease among those living with HIV, and continued support of ART programmes is essential for sustained progress on this front. Still, our estimates suggest that most non-typhoidal salmonella invasive disease cases are not attributable to HIV infection, and reducing the burden of non-typhoidal salmonella invasive disease requires continued efforts to reduce acute malnutrition, prevent and treat malaria, improve access to safe water and sanitation, improve hygiene and food handling practices, and improve diagnostic techniques. Although better data and methods allowed us to improve upon previous estimates of non-typhoidal salmonella invasive disease burden, data limitations remain a crucial challenge. Efforts to reduce the burden of non-typhoidal salmonella invasive disease will hinge on the quality of evidence driving decision making. More population-based studies from selected countries and regions, particularly in Latin America, are essential, as are studies on non-typhoidal salmonella invasive disease risk factors, including HIV, malaria, acute malnutrition, and sickle-cell disease.

Our results have important implications for clinical practice and public health policy. Given the high case fatality of non-typhoidal salmonella invasive disease, especially among elderly people and those living with HIV, clinicians in high-burden areas need to be aware of the scale of the problem and respond with timely and appropriate treatment. The paucity of data and uncertainties about the epidemiology of the disease highlight the urgent need for better screening and surveillance efforts to capture the true burden of non-typhoidal salmonella invasive disease and to better inform policy makers. Despite these challenges, non-typhoidal salmonella invasive disease is clearly a major cause of death and disability that is likely to be responsible for a similar number of deaths and DALYs to salmonella enterocolitis. The inadequate attention paid to non-typhoidal salmonella invasive disease suggests that it remains an under-recognised cause of global morbidity and mortality.

## Data sharing

Data used for this study were extracted from publicly available sources that are listed in the appendix. Further details are available in the online Global Health Data Exchange (GHDx) at http://ghdx.healthdata.org/gbd-2017/data-input-sources.
