# Supplementary material for: The global burden of non-typhoidal salmonella invasive disease: a systematic analysis for the Global Burden of Disease Study 2017
Source: Lancet Infect Dis. 2019 Dec;19(12):1312–24. doi: 10.1016/S1473-3099(19)30418-9 (PMC6892270; doi:10.1016/S1473-3099(19)30418-9)
Supplement: Supplementary appendix [file mmc1.pdf]

THE LANCET Infectious Diseases

## Supplementary webappendix

This webappendix formed part of the original submission and has been peer reviewed. We post it as supplied by the authors.

Supplement to: GBD 2017 Non-Typhoidal Salmonella Invasive Disease Collaborators. The global burden of non-typhoidal salmonella invasive disease: a systematic analysis for the Global Burden of Disease Study 2017. *Lancet Infect Dis* 2019; published online Sept 24. https://doi.org/10.1016/S1473-3099(19)30418-9.

# Contents

**A. Methods Appendix** .......... 2
**A1: GBD Locations** .......... 2
**A2. Systematic Review** .......... 3
**A3. Overview of modelling process** .......... 8
**A4. CodCorrect** .......... 9
**B. Supplemental results** .......... 10
**C. Source Appendix** .......... 52
**C1. Incidence data sources** .......... 52
**C2. Case fatality data sources** .......... 54
**C3. HIV coinfection data sources** .......... 56

## A. Methods Appendix

### A1: GBD Locations

GBD uses a set of hierarchically nested geographies for both analysis and reporting. Seven super-regions are nested within the world (Global); 21 regions are nested within the super-regions; and 195 countries and territories are nested inside of regions. Figure S1 below shows the super-regions and region. Note that North Africa and Middle East, and South Asia are treated as both super-regions and regions (i.e. each of these super-regions has only one region nested within it). All other super-regions contain between three and five regions.

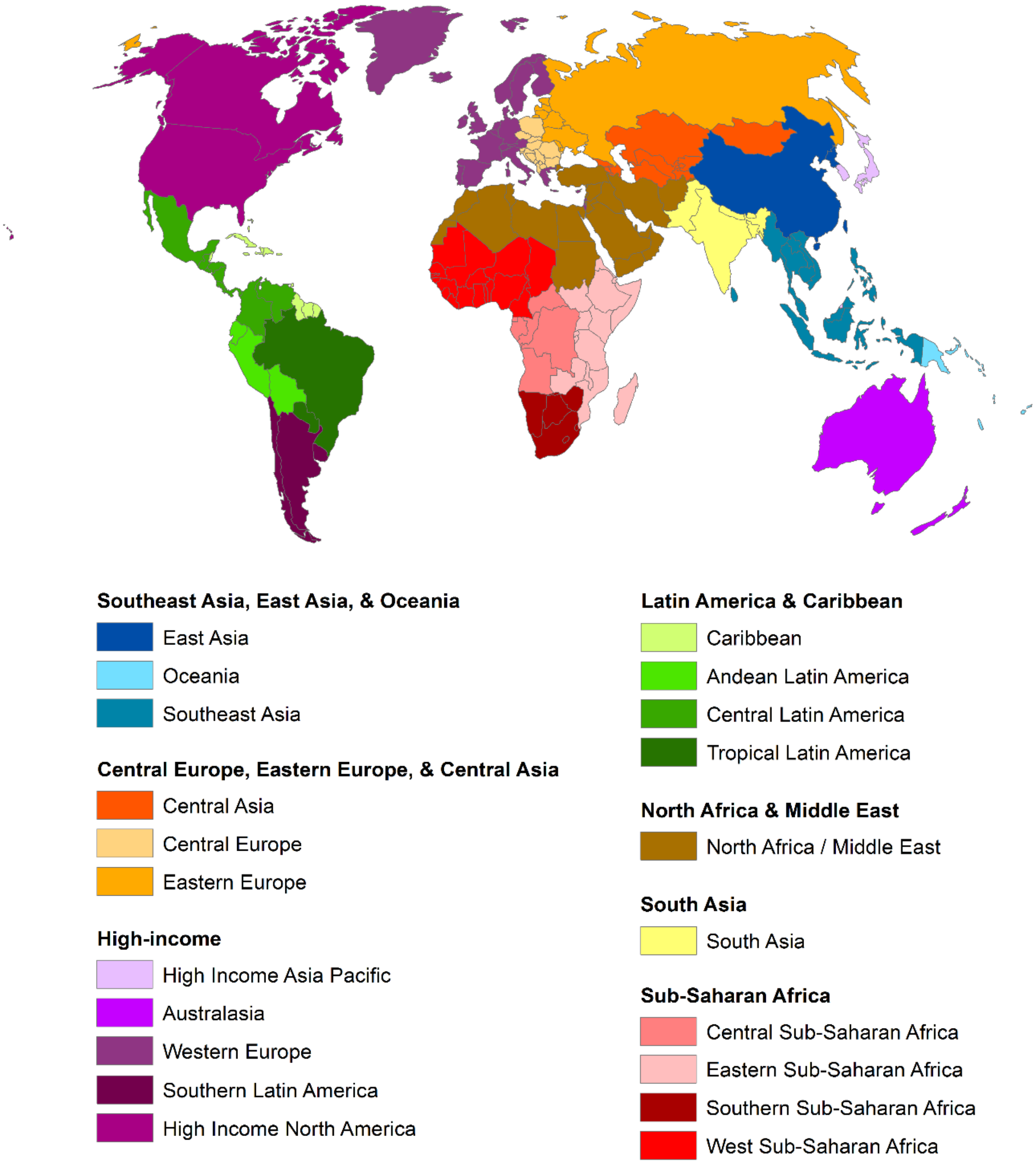


**Figure S1: GBD regions and super-regions**

## A2. Systematic Review

We searched through 1) scientific databases including PubMed (including MEDLINE), Scopus, Web of Science, Western Pacific Region Index Medicus, The Cochrane Library, LILACS, OVID, ANU Super Search, Google Scholar, University of Washington library; 2) grey literature databases including Open Access Theses and Dissertations, Dart Europe, Open Doar, OAIster, OpenGrey, and The World Health Organization (WHO) library; 3) other grey literature sources including the websites of WHO, European Food and Safety Authority, U.S. Food and Drug Administration, Health Canada, Centers for Disease Control and Prevention, The International Centre for Diarrhoeal Disease Research in Bangladesh, The International Vaccine Institute; and the National Institutes of Health Research Portfolio Online Reporting Tools. Reference list search of eligible studies was also performed.

Search strategies were discussed with a librarian using key terminology from primary studies and were specifically tailored for each database (Table S1). We included all studies published between January 1980 and July 19 2017 which reported information on iNTS within at least one of the following categories: incidence, case fatality rate, and risk factors including co-infections with HIV and malaria. We defined iNTS disease as individuals with laboratory confirmed iNTS infection including bloodstream infection, meningitis, or infection of other normally sterile site. All original study designs were included in the review except for editorial letters, case studies, and reviews. No language restrictions were applied.

Identified studies were imported into Endnote X7 and undergone several stages of independent screening by two authors (AP and KS), with any discrepancies discussed with a third author (JDS). The list of inclusion and exclusion criteria for each stage of the systematic review is presented in Table S2. Backward citation screening of eligible studies was also performed. Studies published in languages other than English were reviewed by the researchers familiar with those languages (French, AP and JDS), Spanish (AP), Polish (AP), Czech (AP), and Chinese (JC). Flow diagram of eligible studies is depicted in Figure S2.

Data from included studies was extracted by two authors (AP and JDS) using the Institute for Health Metrics and Evaluation (IHME) extraction tool. Among others, this included data on location, time period, data source type, case definition, case diagnostics, type of measure, sample size, age, sex, representativeness, and urbanicity. Studies reporting incidence or mortality were population-based studies with a clearly defined denominator. We also considered hospital-based studies with a clearly defined catchment population. Where studies reported total sample size and age-specific incidence rates, but not age-specific sample size, we assumed the age distribution of the sample matched that of the population. Where detailed (eg, age-specific rates) data were presented in graphs, we extracted data from graphs using WebPlotDigitizer, version 3.8 (Ankit Rohatgi, Austin Texas). Studies reporting the case fatality rate were included if they were based on ≥ 10 cases.

We included studies presenting data on iNTS-malaria coinfection in our systematic review and extraction with the intention of estimating relative risks comparing iNTS incidence between those with and without malaria. Upon inspection, we found the data too limited to support this analysis and did not use the data on iNTS-malaria coinfection for this study. Data on other components of the analysis (i.e. incidence, case fatality, and HIV coinfection) from studies presenting malaria coinfection data were retained.

**Table S1: Search strategies**

| **PubMed *(n=817)*** |
|---|
| (salmonella OR salmonellosis) AND (nontyph* OR non-typh* OR typhimurium OR enteritidis OR Newport OR Heidelberg) AND (bacteraemi* OR bacteremi* OR septicaemi* OR septicemi* OR septic OR sepsis OR “blood isolate” OR bloodstream OR invasive) AND ( infant OR child OR children OR adolescent OR adult OR patient OR human OR community OR participant OR volunteer OR subject OR incidence OR hospital OR men OR women OR man OR woman) AND (incidence OR prevalence OR burden OR proportion OR "fatality rate" OR "fatality ratio" OR predictor OR determinant OR "risk factor" OR "risk group" OR "at risk" OR environment OR climate OR foodborne OR "food borne" OR zoonosis OR zoonotic OR animal OR waterborne OR "water borne" OR soil OR "land use" OR flood OR drought OR earthquake OR mountain OR "drinking water" OR sanitation OR ecology OR ecological OR "sanitation condition" OR epidemic OR outbreak OR reservoir OR HIV OR "human immunodeficiency virus" OR AIDS OR malaria OR anemia OR anaemia OR malnutrition OR undernutrition OR malnourished)<br>restricted to humans, from 1/1/1980 to 19/07/2017 |

| **Web of Science *(n=837)*** |
|---|
| TS=((salmonella OR salmonellosis) AND ( nontyph* OR non-typh* OR typhimurium OR enteritidis OR Newport OR Heidelberg ) AND ( bacteraemi* OR bacteremi* OR septicaemi* OR septicemi* OR septic OR sepsis OR blood isolate OR bloodstream OR invasive) AND ( infant OR child OR children OR adolescent OR adult OR patient OR human OR community OR participant OR volunteer OR subject OR incidence OR hospital OR men OR women OR man OR woman ) AND ( incidence OR prevalence OR burden OR proportion OR "fatality rate" OR "fatality ratio" OR predictor OR determinant OR "risk factor" OR "risk group" OR "at risk" OR environment OR climate OR foodborne OR "food borne" OR zoonosis OR zoonotic OR animal OR waterborne OR "water borne" OR soil OR "land use" OR flood OR drought OR earthquake OR mountain OR "drinking water" OR sanitation OR ecology OR ecological OR "sanitation condition" OR epidemic OR outbreak OR reservoir OR HIV OR "human immunodeficiency virus" OR AIDS OR malaria OR anemia OR anaemia OR malnutrition OR undernutrition OR malnourished))<br>Indexes=SCI-EXPANDED, SSCI, A&HCI, CPCI-S, CPCI-SSH, ESCI, CCR-EXPANDED, IC Timespan=1990-2017 |
| **Scopus *(n=1237)*** |
| TITLE-ABS-KEY ( ( salmonella OR salmonellosis ) AND ( nontyph* OR non-typh* OR typhimurium OR enteritidis OR newport OR heidelberg ) AND ( bacteraemi* OR bacteremi* OR septicaemi* OR septicemi* OR septic OR sepsis OR "blood isolate" OR bloodstream OR invasive ) AND ( infant OR child OR children OR adolescent OR adult OR patient OR human OR community OR participant OR volunteer OR subject OR incidence OR hospital OR men OR women OR man OR woman ) AND ( incidence OR prevalence OR burden OR proportion OR "fatality rate" OR "fatality ratio" OR predictor OR determinant OR "risk factor" OR "risk group" OR "at risk" OR environment OR climate OR foodborne OR "food borne" OR zoonosis OR zoonotic OR animal OR waterborne OR "water borne" OR soil OR "land use" OR flood OR drought OR earthquake OR mountain OR "drinking water" OR sanitation OR ecology OR ecological OR "sanitation condition" OR epidemic OR outbreak OR reservoir OR hiv OR "human immunodeficiency virus" OR aids OR malaria OR anemia OR anaemia OR malnutrition OR undernutrition OR malnourished ) ) AND ( LIMIT-TO ( PUBYEAR , 2017 ) OR LIMIT-TO ( PUBYEAR , 2016 ) OR LIMIT-TO ( PUBYEAR , 2015 ) OR LIMIT-TO ( PUBYEAR , 2014 ) OR LIMIT-TO ( PUBYEAR , 2013 ) OR LIMIT-TO ( PUBYEAR , 2012 ) OR LIMIT-TO ( PUBYEAR , 2011 ) OR LIMIT-TO ( PUBYEAR , 2010 ) OR LIMIT-TO ( PUBYEAR , 2009 ) OR LIMIT-TO ( PUBYEAR , 2008 ) OR LIMIT-TO ( PUBYEAR , 2007 ) OR LIMIT-TO ( PUBYEAR , 2006 ) OR LIMIT-TO ( PUBYEAR , 2005 ) OR LIMIT-TO ( PUBYEAR , 2004 ) OR LIMIT-TO ( PUBYEAR , 2003 ) OR LIMIT-TO ( PUBYEAR , 2002 ) OR LIMIT-TO ( PUBYEAR , 2001 ) OR LIMIT-TO ( PUBYEAR , 2000 ) OR LIMIT-TO ( PUBYEAR , 1999 ) OR LIMIT-TO ( PUBYEAR , 1998 ) OR LIMIT-TO ( PUBYEAR , 1997 ) OR LIMIT-TO ( PUBYEAR , 1996 ) OR LIMIT-TO ( PUBYEAR , 1995 ) OR LIMIT-TO ( PUBYEAR , 1994 ) OR LIMIT-TO ( PUBYEAR , 1993 ) OR LIMIT-TO ( PUBYEAR , 1992 ) OR LIMIT-TO ( PUBYEAR , 1991 ) OR LIMIT-TO ( PUBYEAR , 1990 ) OR LIMIT-TO ( PUBYEAR , 1989 ) OR LIMIT-TO ( PUBYEAR , 1988 ) OR LIMIT-TO ( PUBYEAR , 1987 ) OR LIMIT-TO ( PUBYEAR , 1986 ) OR LIMIT-TO ( PUBYEAR , 1985 ) OR LIMIT-TO ( PUBYEAR , 1984 ) OR LIMIT-TO ( PUBYEAR , 1983 ) OR LIMIT-TO ( PUBYEAR , 1982 ) OR LIMIT-TO ( PUBYEAR , 1981 ) OR LIMIT-TO ( PUBYEAR , 1980 ) ) |
| **WPRIM *(n=42)*** |
| Limits: 1980-2017; Humans<br>All:Salmonella or All:Salmonellosis |
| **Lilacs *(n=37)*** |
| ((tw:((salmonella OR salmonellosis) AND ( nontyph* OR non-typh* OR typhimurium OR enteritidis OR newport OR heidelberg) AND ( bacteraemi* OR bacteremi* OR septicaemi* OR septicemi* OR septic OR sepsis OR bloodstream OR "blood isolate" OR invasive) ) AND (instance:"regional") AND ( collection:("01-internacional") AND db:("LILACS") AND year_cluster:("1992" OR "1995" OR "2011" OR "1990" OR "2003" OR "2010" OR "2012" OR "2014" OR "2000" OR "2004" OR "2006" OR "2009" OR "2013" OR "1991" OR "1994" OR "1996" OR "2002" OR "2007" OR "2016" OR "1997" OR "1998" OR "1999" OR "2015" OR "2017" OR "2004" OR "2005" OR "2008" OR "1980" OR "1981" OR "1982" OR "1983" OR "1984" OR "1985" OR "1986" OR "1987" OR "1988" OR "1989")))) AND ( limit:("humans")) |
| **Cochrane *(n=11)*** |
| (salmonella OR salmonellosis) AND ( nontyph* OR non-typh* OR typhimurium OR enteritidis OR Newport OR Heidelberg ) AND (bacteraemi* OR bacteremi* OR septicaemi* OR septicemi* OR septic OR sepsis OR "blood isolate" OR bloodstream OR invasive) AND ( infant OR child OR children OR adolescent OR adult OR patient OR human OR community OR participant OR volunteer OR subject OR incidence OR hospital OR men OR women OR man OR woman) AND (incidence OR prevalence OR burden OR proportion OR "fatality rate" OR "fatality ratio" OR predictor OR determinant OR "risk factor" OR "risk group" OR "at risk" OR environment OR climate OR foodborne OR "food borne" OR zoonosis OR zoonotic OR animal OR waterborne OR "water borne" OR soil OR "land use" OR flood OR drought OR earthquake OR mountain OR "drinking water" OR sanitation OR ecology OR ecological OR "sanitation condition" OR epidemic OR outbreak OR reservoir OR HIV OR "human immunodeficiency virus" OR AIDS OR malaria OR anemia OR anaemia OR malnutrition OR undernutrition OR malnourished)<br>Publication Year from 1980 to 2017, in Cochrane Reviews (Reviews and Protocols), Other Reviews, Trials, Methods Studies, Technology Assessments, Economic Evaluations and Cochrane Groups (Word variations have been searched) |
| **ANU Super Search *(n=804)*** |
| Filter: 1/1/1980 – 19/7/2017, add results beyond your library collection |

| |
|---|
| (Abstract:(salmonella OR salmonellosis)) AND (Abstract:(nontyph* OR non-typh* OR typhimurium OR enteritidis OR newport OR heidelberg)) AND (Abstract:(bacteraemi* OR bacteremi* OR septicaemi* OR septicemi* OR septic OR sepsis OR "blood isolate" OR bloodstream OR invasive)) AND (infant OR child OR children OR adolescent OR adult OR patient OR human OR community OR participant OR volunteer OR subject OR incidence OR hospital OR men OR women OR man OR woman) AND (incidence OR prevalence OR burden OR proportion OR "fatality rate" OR "fatality ratio" OR predictor OR determinant OR "risk factor" OR "risk group" OR "at risk" OR environment OR climate OR foodborne OR "food borne" OR zoonosis OR zoonotic OR animal OR waterborne OR "water borne" OR soil OR "land use" OR flood OR drought OR earthquake OR mountain OR "drinking water" OR sanitation OR ecology OR ecological OR "sanitation condition" OR epidemic OR outbreak OR reservoir OR HIV OR "human immunodeficiency virus" OR AIDS OR malaria OR anemia OR anaemia OR malnutrition OR undernutrition OR malnourished)<br>Excluded: veterinary medicine, economics, physics, engineering, forestry, mathematics, dentistry, zoology, botany, agriculture, |
| **Google Scholar *(n=510)*** |
| (salmonella OR salmonellosis) AND (nontyph* OR non-typh* OR typhimurium OR enteritidis OR newport OR heidelberg) AND (bacteraemia* OR bacteremi* OR septicaemi* OR septicemi* OR septic OR sepsis OR bloodstream OR "blood isolate" OR invasive)<br>Filter: 1980 - 2017 |
| **Ovid *(n=216)*** |
| ((salmonella or salmonellosis) and (nontyph* or non-typh* or typhimurium or enteritidis or newport or heidelberg) and (bacteraemi* or bacteremi* or septicaemi* or septicemi* or septic or sepsis or bloodstream or "blood isolate" or invasive) and (infant or child or children or adolescent or adult or patient or human or community or participant or volunteer or subject or incidence or hospital or men or women or man or woman) and (incidence or prevalence or burden or proportion or "fatality rate" or "fatality ratio" or predictor or determinant or "risk factor" or "risk group" or "at risk" or environment or climate or foodborne or "food borne" or zoonosis or zoonotic or animal or waterborne or "water borne" or soil or "land use" or flood or drought or earthquake or mountain or "drinking water" or sanitation or ecology or ecological or "sanitation condition" or epidemic or outbreak or reservoir or HIV or "human immunodeficiency virus" or AIDS or malaria or anemia or anaemia or malnutrition or undernutrition or malnourished)).mp. [mp=title, abstract, full text, caption text]<br>Limit 1 to yr="1980-Current" |
| **OATD *(n=31)*** |
| (salmonella OR salmonellosis) AND (nontyph* OR non-typh* OR typhimurium OR enteritidis OR newport OR heidelberg) AND (bacteraemi* OR bacteremi* OR septicaemi* OR septicemi* OR septic OR sepsis OR bloodstream OR "blood isolate" OR invasive) AND ( infant OR child OR children OR adolescent OR adult OR patient OR human OR community OR participant OR volunteer OR subject OR incidence OR hospital OR men OR women OR man OR woman ) AND ( incidence OR prevalence OR burden OR proportion OR "fatality rate" OR "fatality ratio" OR predictor OR determinant OR "risk factor" OR "risk group" OR "at risk" OR environment OR climate OR foodborne OR "food borne" OR zoonosis OR zoonotic OR animal OR waterborne OR "water borne" OR soil OR "land use" OR flood OR drought OR earthquake OR mountain OR "drinking water" OR sanitation OR ecology OR ecological OR "sanitation condition" OR epidemic OR outbreak OR reservoir OR HIV OR "human immunodeficiency virus" OR AIDS OR malaria OR anemia OR anaemia OR malnutrition OR undernutrition OR malnourished) |
| **Dart Europe *(n=8)*** |
| (salmonella OR salmonellosis) AND (nontyph* OR non-typh* OR typhimurium OR enteritidis OR newport OR heidelberg) AND ( bacteraemi* OR bacteremi* OR septicaemi* OR septicemi* OR septic OR sepsis OR bloodstream OR "blood isolate" OR invasive) AND ( infant OR child OR children OR adolescent OR adult OR patient OR human OR community OR participant OR volunteer OR subject OR incidence OR hospital OR men OR women OR man OR woman ) AND ( incidence OR prevalence OR burden OR proportion OR "fatality rate" OR "fatality ratio" OR predictor OR determinant OR "risk factor" OR "risk group" OR "at risk" OR environment OR climate OR foodborne OR "food borne" OR zoonosis OR zoonotic OR animal OR waterborne OR "water borne" OR soil OR "land use" OR flood OR drought OR earthquake OR mountain OR "drinking water" OR sanitation OR ecology OR ecological OR "sanitation condition" OR epidemic OR outbreak OR reservoir OR HIV OR "human immunodeficiency virus" OR AIDS OR malaria OR anemia OR anaemia OR malnutrition OR undernutrition OR malnourished) |
| **Open Doar *(n=0)*** |
| Salmonella |
| **OAISTER *(n=7)*** |
| kw:salmonella OR salmonellosis) AND (nontyph* OR non-typh* OR typhimurium OR enteritidis OR newport OR heidelberg) AND ( bacteraemi* OR bacteremi* OR septicaemi* OR septicemi* OR septic OR sepsis OR bloodstream OR "blood isolate" OR invasive) AND ( infant OR child OR children OR adolescent OR adult OR patient OR human OR community OR participant OR volunteer OR subject OR incidence OR hospital OR men OR women OR man OR woman ) AND ( incidence OR prevalence OR burden OR proportion OR "fatality rate" OR "fatality ratio" OR predictor OR determinant OR "risk factor" OR "risk group" OR "at risk" OR environment OR climate OR foodborne OR "food borne" OR zoonosis OR zoonotic OR animal OR waterborne OR "water borne" OR soil OR "land use" OR flood OR drought OR earthquake OR mountain OR "drinking water" OR sanitation OR ecology OR ecological OR "sanitation condition" OR epidemic OR outbreak OR reservoir OR HIV OR "human immunodeficiency virus" OR AIDS OR malaria OR anemia OR anaemia OR malnutrition OR undernutrition OR malnourished)<br>Limited to:1980 – 2017 |
| **Open Grey *(n=9)*** |
| (salmonella OR salmonellosis) AND (nontyph* OR non-typh* OR typhimurium OR enteritidis OR newport OR heidelberg) AND (bacteraemi* OR bacteremi* OR septicaemi* OR septicemi* OR septic OR sepsis OR bloodstream OR "blood isolate" OR invasive) |

**Table S2: Inclusion and exclusion criteria at each stage of the review process**

| | Inclusion criteria | Exclusion criteria |
|---|---|---|
| **Title selection** | The primary study outcome is related to nontyphoidal *Salmonella* (NTS).<br>Study is published from 1980 onwards. | Study outcome is related to *Salmonella* typhi or *Salmonella* paratyphi A, B, or C.<br>Study is related to animal NTS isolates.<br>Study is published before 1980.<br>Study focuses on analysis of gene, protein or molecular factors related to *Salmonella.* |
| **Abstract selection** | Study reports on invasive nontyphoidal *Salmonella* (iNTS) disease defined as laboratory confirmed human NTS infection with samples coming from blood, cerebrospinal fluid, synovial fluid, or other normally sterile site.<br>Study is an original article (case-control study, cohort study, outbreak investigation, case-series, survey, or thesis) | Study reports on NTS enterocolitis or data is based on the analysis of NTS coming from stool, urine, abscess, wounds, skin, or other non-sterile sites.<br>Study is not an original research article (editorial letter, comments).<br>Study is a review. |
| **Full-text selection – qualitative synthesis** | Study reports iNTS incidence, case fatality rate/mortality, and risk factors including co-infections with HIV and malaria. | Study does not provide a clear denominator.<br>Study does not provide data in an extractable format.<br>Study uses duplicate data.<br>Study is not representative of iNTS.<br>Study is not population representative.<br>Study is based on less than 10 iNTS cases.<br>Study reports only specific iNTS serovar. |
| **Full-text selection – quantitative synthesis** | Study reporting incidence or mortality is population-based study with a clearly defined denominator, or it is a hospital-based study with a clearly defined catchment population.<br>Study is based on >10 iNTS cases.<br>Study provides data in an extractable format. | |

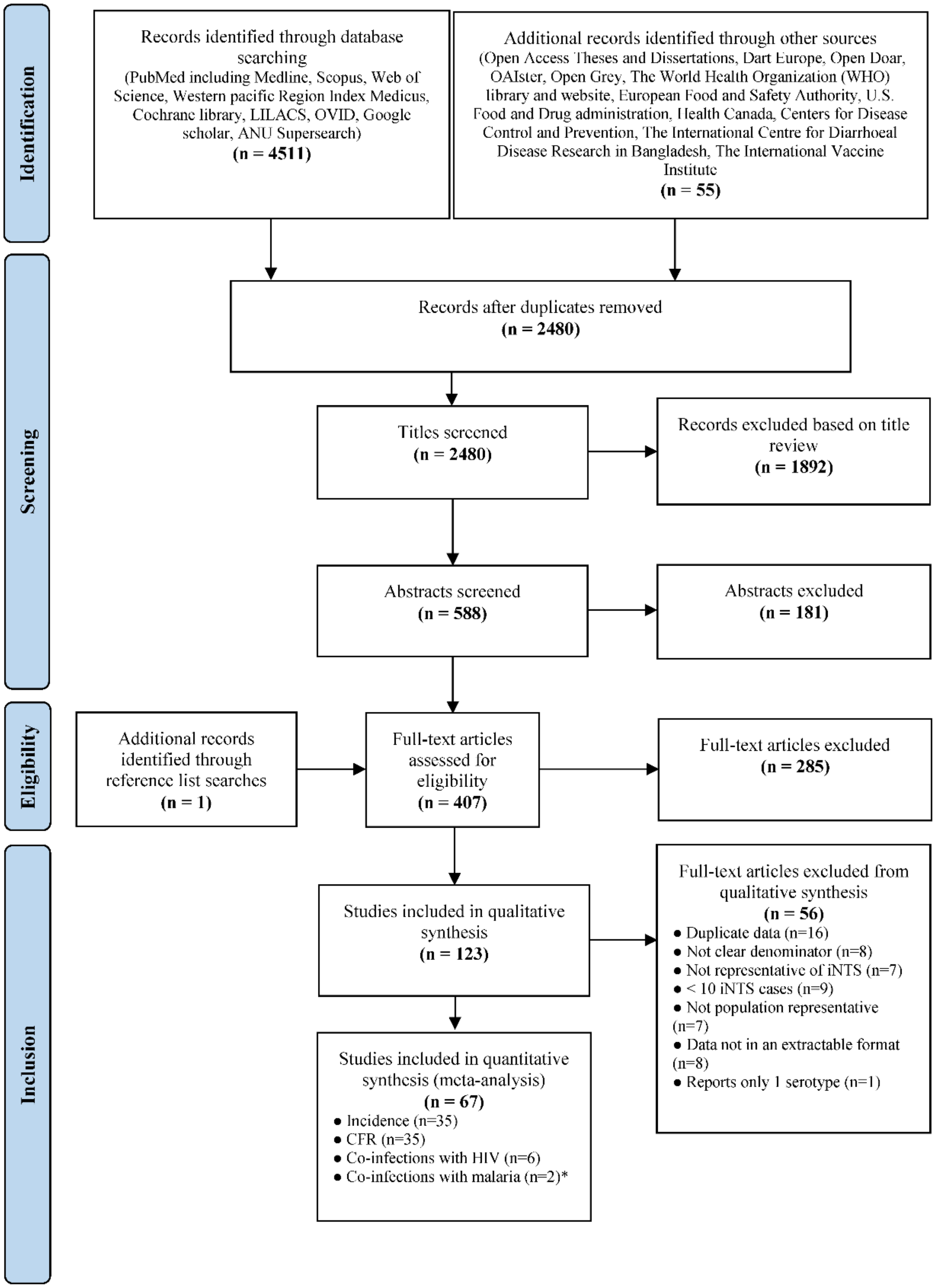


*Data on malaria coinfection were extracted, but not used in the current analysis (see section A2).

**Figure S2: Systematic review flow chart**

## A3. Overview of modelling process

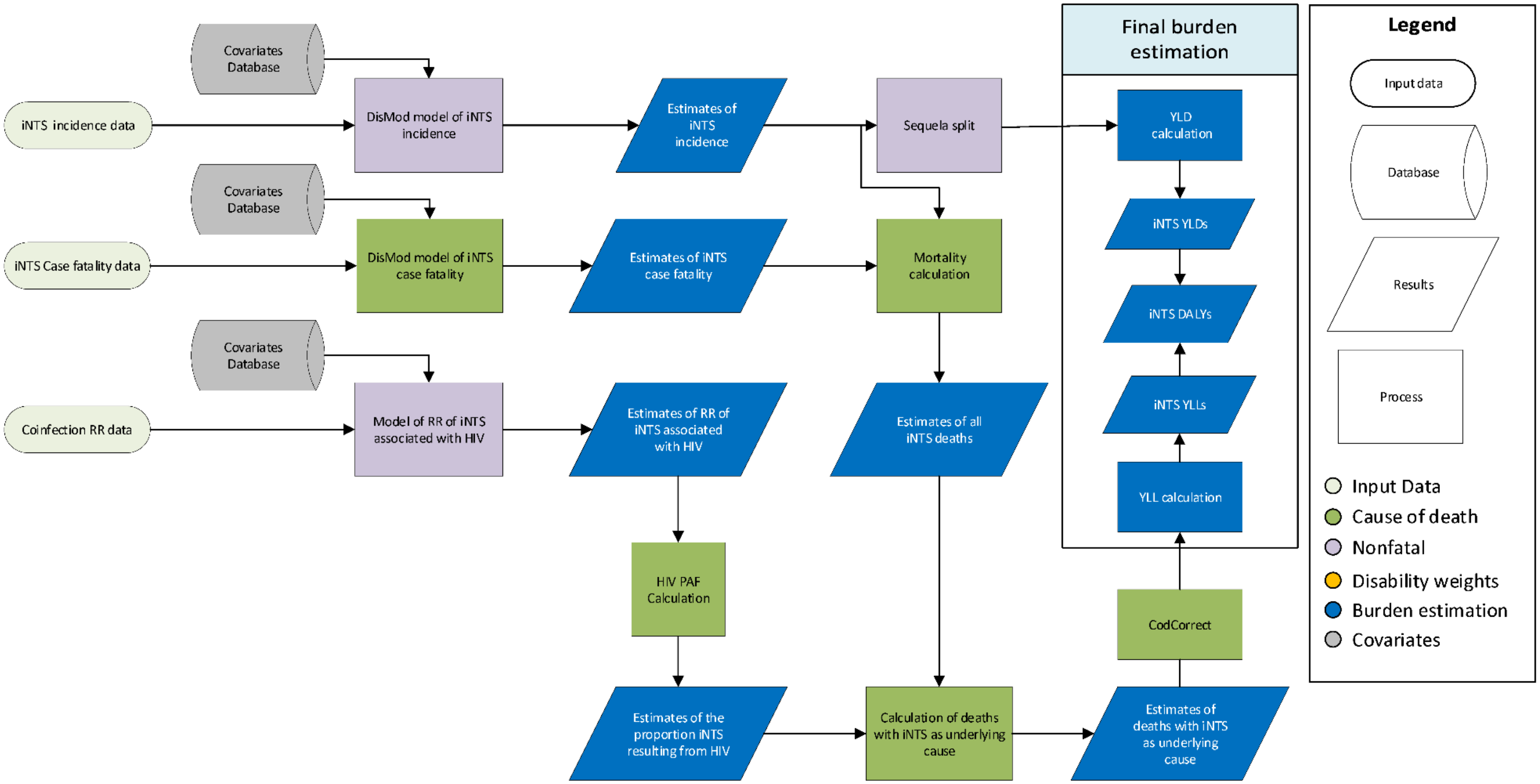


**Figure S3: Flow diagram of modelling process**

## A4. CodCorrect

Within the GBD study, we impose two constraints on estimates of cause-specific mortality to ensure consistency: 1) each death has one underlying cause, and 2) all cause-specific mortality estimates must sum to equal total all-cause mortality within every location, year, sex, and age group. CoDCorrect is the hierarchical rescaling process that we use to impose the second constraint, and it has been previously described in detail.[1] In general, cause-specific mortality estimates will have greater uncertainty in locations and years where we have less cause-specific mortality data. Where the estimates are less certain, the aggregated cause-specific estimates are likely to deviate more greatly from the all-cause mortality estimate, and the effect of CoDCorrect is likely to be more pronounced. Note that data sparsity here, refers not just to data on iNTS, but to data on all causes of death. Conversely, where cause-specific mortality data are nearly complete (i.e. where the underlying cause is known for nearly every death), we expect the aggregated cause-specific estimates to nearly match the all-cause mortality estimate, and expect CoDCorrect to have a negligible effect on the final estimates. In the case of iNTS most deaths occurred in those locations for which very little data are available across all years (e.g. countries of Eastern, Central, and Western sub-Saharan Africa). Because the absence of cause-specific mortality data is relatively constant across time in these countries, trends in the effect of CoDCorrect on iNTS are more difficult to explain. Broadly, as it relates to locations with a large iNTS burden, the effects of CoDCorrect result from cause-specific models (across the full spectrum of GBD causes) having had the tendency to produce over-estimates that required adjustment downward to fit within the all-cause mortality envelope in earlier years; and having had the tendency to produce under-estimates that required adjustment upward to fill the all-cause mortality envelope in later years.

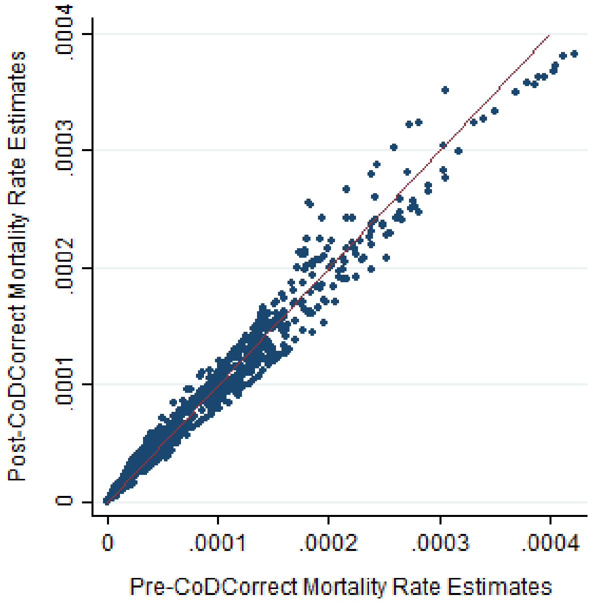


**Figure S4: Comparison of iNTS mortality rate estimates before and after CoDCorrect, for each country and year.** The red line shows equality (ie. points falling on this line were unchanged by CoDCorrect).

**Table S3:** Comparison of global estimates of iNTS deaths before and after CoDCorrect, by year

| Year | Pre-CodCorrect | Post-CodCorrect | Absolute Change | Percent Change |
|---|---|---|---|---|
| 1990 | 45,965 | 42,782 | -3,182 | -6.90% |
| 1995 | 51,167 | 48,580 | -2,588 | -5.10% |
| 2000 | 62,646 | 59,058 | -3,588 | -5.70% |
| 2005 | 80,594 | 74,809 | -5,785 | -7.20% |
| 2010 | 70,284 | 67,563 | -2,721 | -3.90% |
| 2015 | 57,503 | 61,679 | 4,176 | 7.30% |
| 2017 | 53,645 | 59,066 | 5,421 | 10.10% |

## B. Supplemental results

**A)**

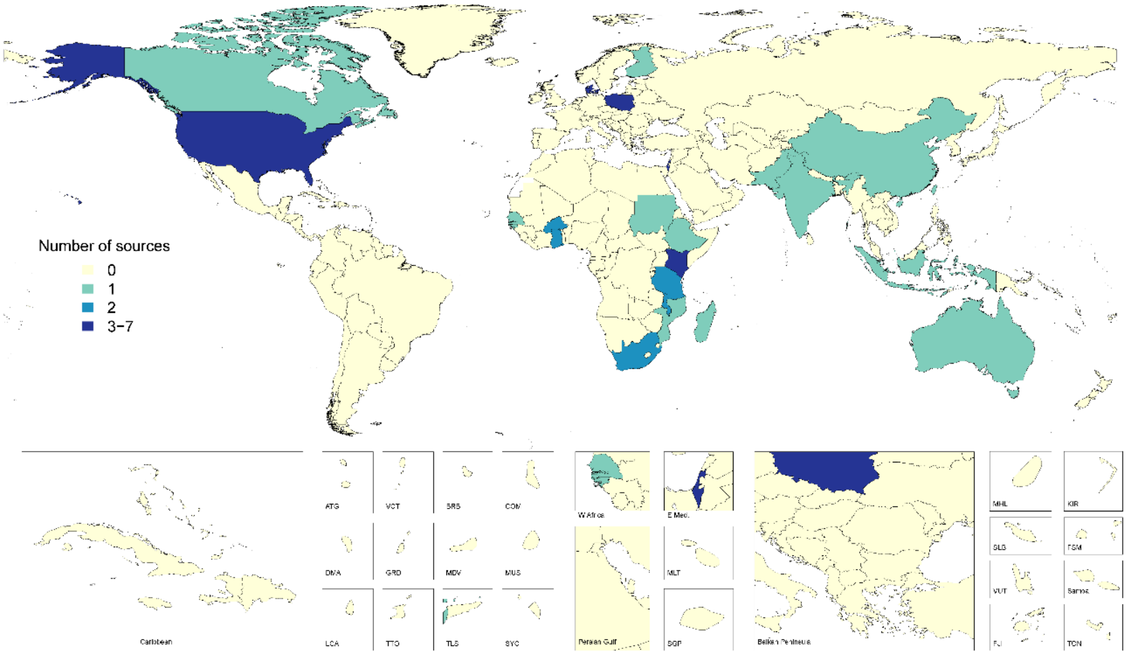


**B)**

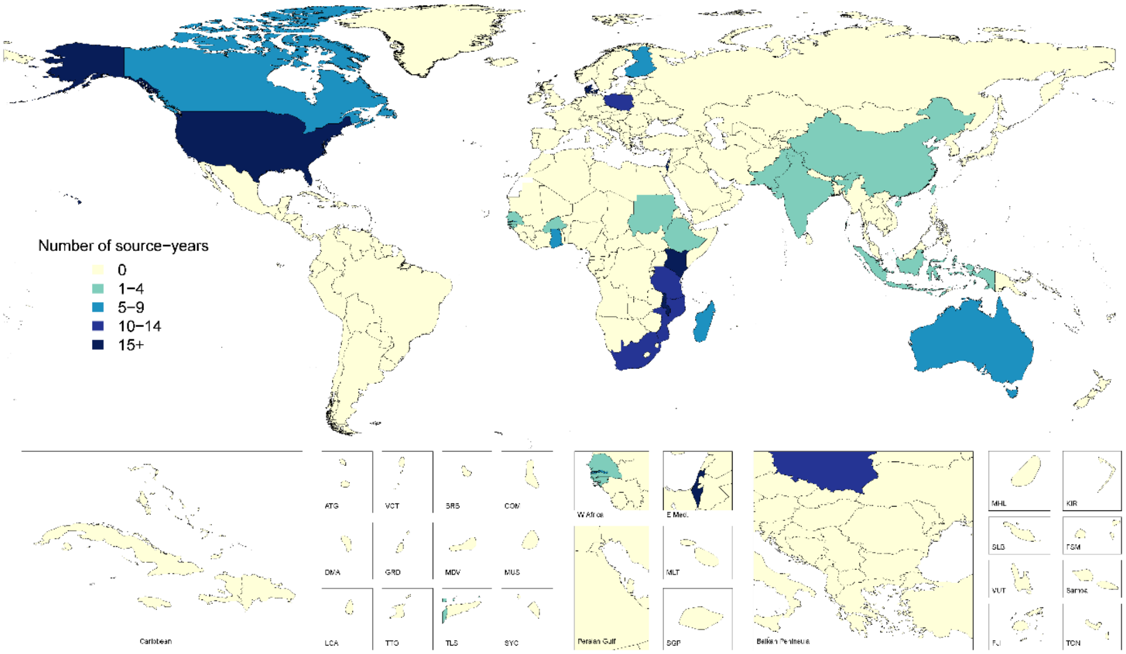


**Figure S5: The number of incidence data sources, by country, excluding vital registration sources (A), and the number of source-years, by country, including vital registration sources (B).** Source-years are calculated for each location as the sum of the number of years of observation across all sources.

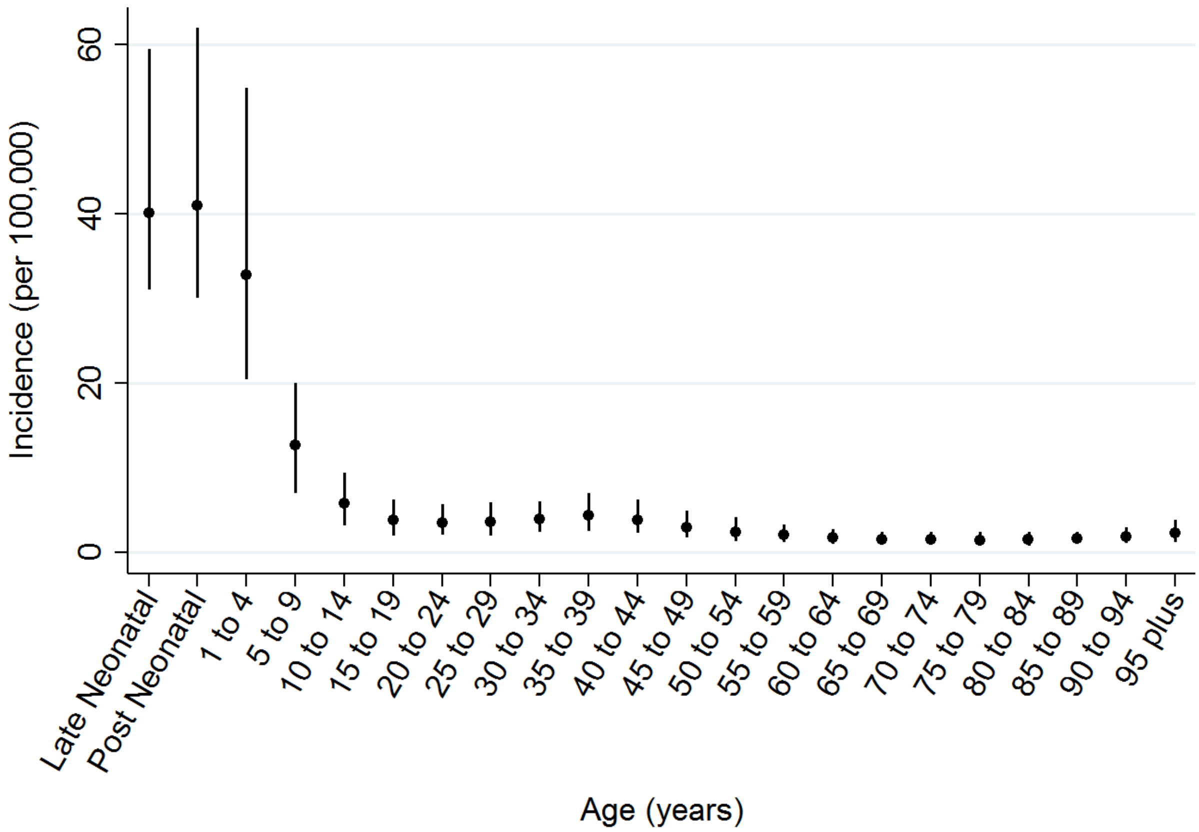


**Figure S6: Global incidence by age in 2017. 95%** Uncertainty intervals are shown.

**Table S4: iNTS case fatality, with 95% uncertainty intervals, among those living with and without HIV, and the total population, by year, super-region, age, and sex.** Rates are age-standardized, except for age-specific estimates.

| | Total case-fatality (%) | | Case fatality among HIV- (%) | | Case fatality among HIV+ (%) | |
|---|---|---|---|---|---|---|
| | **All-age / Age-specific** | **Age-standardized** | **All-age/Age-specific** | **Age-standardized** | **All-age/Age-specific** | **Age-standardized** |
| **Year** | | | | | | |
| 1990 | 15.16 (9.37, 22.21) | 15.93 (9.85, 23.35) | 13.18 (7.83, 19.73) | 13.69 (8.08, 20.73) | 49.92 (37.36, 62.07) | 50.39 (37.83, 62.46) |
| 1995 | 16.21 (10.31, 23.29) | 16.87 (10.70, 24.14) | 12.98 (7.81, 19.39) | 13.43 (8.03, 20.08) | 49.12 (36.65, 61.27) | 49.60 (37.13, 61.67) |
| 2000 | 16.63 (10.60, 23.82) | 16.96 (10.81, 24.25) | 12.73 (7.64, 19.17) | 13.03 (7.81, 19.58) | 48.69 (36.27, 60.82) | 49.10 (36.68, 61.17) |
| 2005 | 15.64 (9.98, 22.39) | 15.66 (9.95, 22.38) | 12.09 (7.32, 18.11) | 12.23 (7.39, 18.26) | 47.53 (35.22, 59.65) | 47.82 (35.51, 59.91) |
| 2010 | 14.63 (9.24, 21.23) | 14.47 (9.11, 21.02) | 11.87 (7.23, 17.81) | 11.88 (7.24, 17.80) | 45.45 (33.33, 57.58) | 45.60 (33.47, 57.73) |
| 2017 | 14.52 (9.18, 21.06) | 14.18 (8.99, 20.65) | 12.03 (7.35, 17.99) | 11.97 (7.31, 17.89) | 41.82 (30.03, 53.97) | 41.82 (29.98, 54.02) |
| **Super-region** | | | | | | |
| Southeast Asia, East Asia, & Oceania | 8.25 (4.77, 12.79) | 7.44 (4.31, 11.69) | 7.66 (4.35, 12.08) | 7.02 (4.00, 11.19) | 31.77 (21.09, 43.86) | 30.75 (19.76, 43.35) |
| Central Europe, Eastern Europe, & Central Asia | 9.36 (5.21, 14.87) | 7.86 (4.33, 12.67) | 8.49 (4.59, 13.97) | 7.15 (3.89, 11.73) | 30.58 (19.93, 42.64) | 27.50 (16.97, 40.00) |
| High-income | 12.74 (6.87, 20.31) | 8.18 (4.35, 13.52) | 8.07 (3.69, 15.00) | 5.18 (2.52, 9.28) | 30.46 (18.85, 43.15) | 23.92 (13.76, 36.46) |
| Latin America & Caribbean | 9.38 (5.55, 14.21) | 9.26 (5.46, 13.99) | 7.75 (4.40, 12.31) | 7.75 (4.41, 12.23) | 32.06 (21.05, 44.52) | 32.14 (21.05, 44.67) |
| North Africa & Middle East | 10.50 (6.06, 16.28) | 11.04 (6.33, 17.12) | 10.37 (5.94, 16.15) | 10.91 (6.24, 17.02) | 36.10 (24.61, 48.62) | 36.96 (25.41, 49.44) |
| South Asia | 9.57 (5.61, 14.74) | 10.15 (5.90, 15.61) | 9.31 (5.41, 14.45) | 9.89 (5.69, 15.26) | 36.50 (24.79, 49.19) | 37.36 (25.67, 49.89) |
| Sub-Saharan Africa | 15.81 (10.01, 22.89) | 19.38 (12.29, 27.70) | 12.94 (7.98, 19.26) | 14.69 (8.69, 22.30) | 43.84 (31.93, 55.90) | 45.46 (33.72, 57.13) |
| **Age** | | | | | | |
| Under 5 | 13.50 (8.37, 19.75) | - | 13.38 (8.31, 19.52) | - | 43.87 (31.97, 55.90) | - |
| 5-14 years | 9.25 (5.62, 14.13) | - | 8.77 (5.30, 13.40) | - | 38.50 (26.29, 51.51) | - |
| 15-49 years | 15.38 (9.54, 22.79) | - | 8.86 (4.68, 15.15) | - | 36.90 (25.65, 49.02) | - |
| 50-69 years | 32.87 (22.97, 44.25) | - | 24.92 (14.86, 38.28) | - | 57.55 (47.01, 66.98) | - |
| 70+ years | 51.20 (30.15, 72.93) | - | 45.03 (23.71, 70.50) | - | 75.71 (57.98, 88.14) | - |
| **Sex** | | | | | | |
| Males | 14.39 (9.09, 21.18) | 14.19 (8.94, 20.90) | 12.04 (7.33, 18.18) | 12.04 (7.35, 18.22) | 41.89 (30.11, 54.03) | 41.98 (30.14, 54.16) |
| Females | 14.65 (9.27, 21.09) | 14.20 (9.00, 20.55) | 12.03 (7.34, 17.78) | 11.91 (7.29, 17.65) | 41.75 (29.94, 53.92) | 41.67 (29.83, 53.89) |

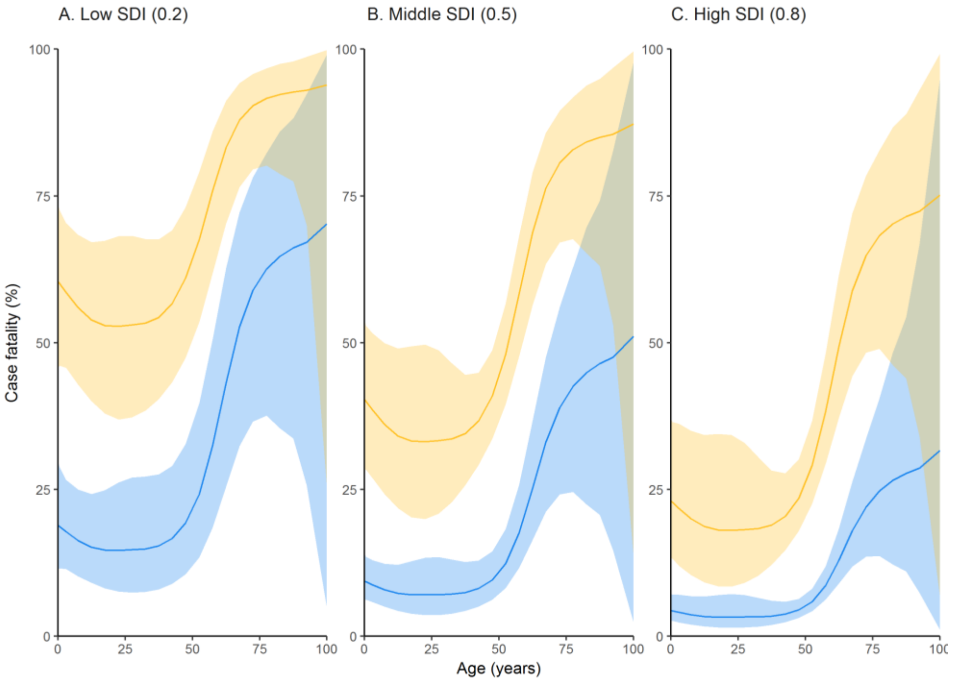


**Figure S7: Case fatality among those with HIV (yellow), and those without HIV (blue), by age and sociodemographic index (SDI).** Lines show point estimates, and shaded areas show 95% uncertainty intervals.

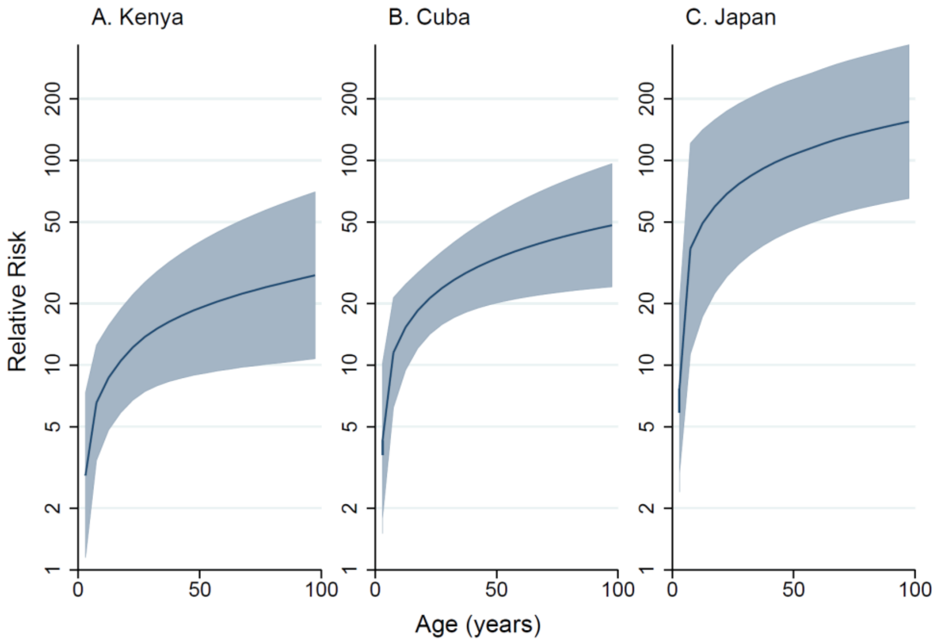


**Figure S8: The relative risk (RR) of iNTS disease comparing those with HIV to those without, by age, for three countries selected to represent those with high (A), moderate (B), and low (C) underlying risk for diarrheal disease.** Lines show point estimates, and shaded areas show 95% uncertainty intervals. Note that the y-axis is on a log-scale.

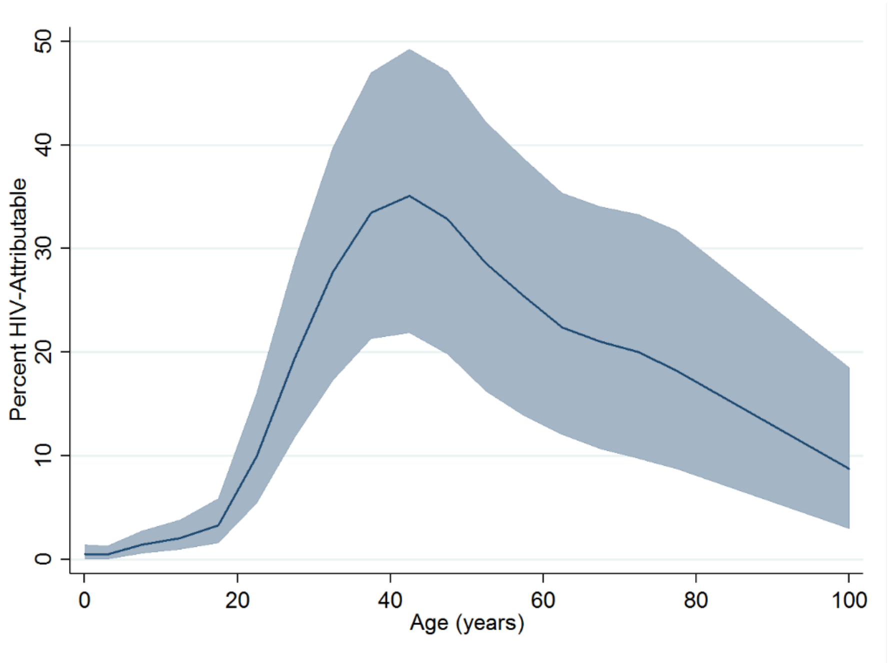


**Figure S9: The percent of iNTS cases that were attributable to HIV, globally, by age.** Lines show point estimates, and shaded areas show 95% uncertainty intervals.

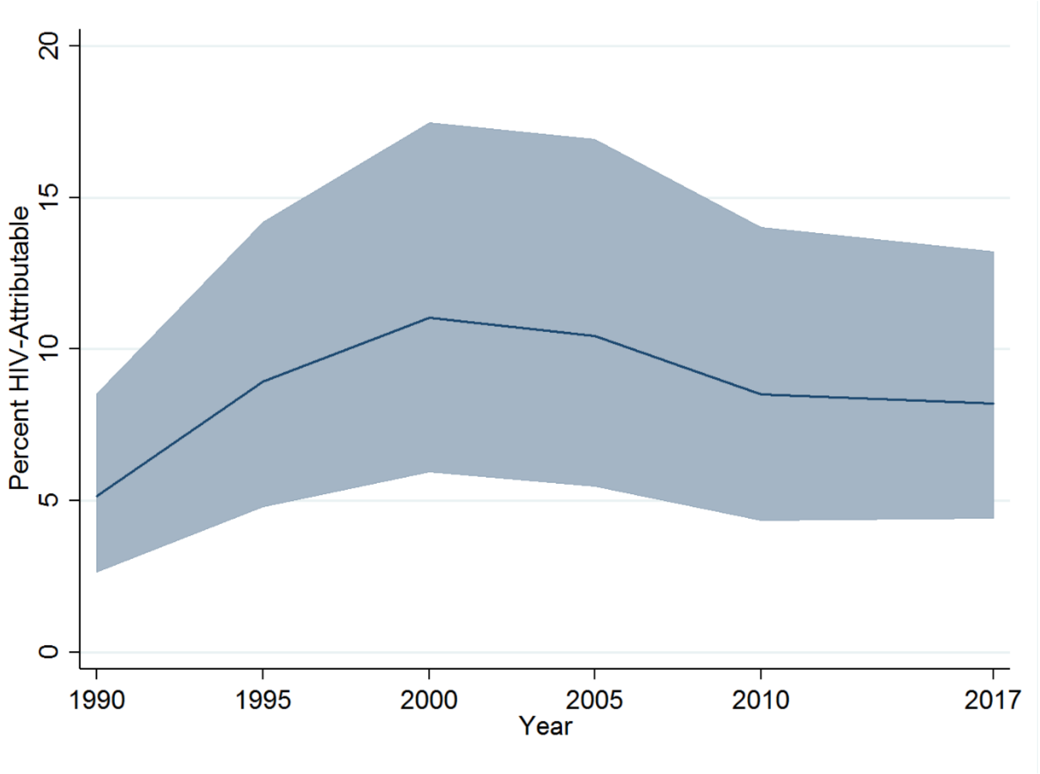


**Figure S10: The percent of iNTS cases that were attributable to HIV, globally, by year.** Lines show point estimates, and shaded areas show 95% uncertainty intervals.

**Table S5: Estimated numbers of iNTS cases, by country, with 95% uncertainty intervals, in 1990, 2005, and 2017.**

| Country | 1990 | 2005 | 2017 |
|---|---|---|---|
| Afghanistan | 458<br>(355 – 578) | 2,120<br>(1,620 – 2,754) | 2,837<br>(2,247 – 3,613) |
| Albania | 43<br>(31 – 57) | 29<br>(20 – 39) | 24<br>(17 – 31) |
| Algeria | 448<br>(324 – 600) | 731<br>(545 – 955) | 609<br>(443 – 794) |
| American Samoa | 2<br>(1 – 3) | 2<br>(1 – 4) | 1<br>(1 – 2) |
| Andorra | 0<br>(0 – <1) | 0<br>(0 – 1) | 0<br>(0 – 1) |
| Angola | 2,373<br>(1,883 – 3,172) | 8,718<br>(6,979 – 11,987) | 5,533<br>(4,389 – 7,322) |
| Antigua and Barbuda | 1<br>(1 – 2) | 2<br>(1 – 2) | 2<br>(1 – 2) |
| Argentina | 364<br>(260 – 487) | 326<br>(222 – 443) | 341<br>(226 – 466) |
| Armenia | 48<br>(35 – 62) | 34<br>(24 – 46) | 31<br>(22 – 41) |
| Australia | 98<br>(62 – 142) | 121<br>(78 – 168) | 150<br>(97 – 211) |
| Austria | 42<br>(28 – 59) | 45<br>(31 – 63) | 50<br>(32 – 69) |
| Azerbaijan | 118<br>(84 – 154) | 125<br>(88 – 162) | 125<br>(91 – 164) |
| Bahrain | 7<br>(4 – 9) | 13<br>(9 – 17) | 18<br>(13 – 25) |
| Bangladesh | 5,219<br>(3,132 – 7,971) | 6,373<br>(4,133 – 9,455) | 6,696<br>(4,715 – 9,484) |
| Barbados | 4<br>(3 – 5) | 4<br>(3 – 5) | 4<br>(3 – 5) |
| Belarus | 137<br>(103 – 178) | 102<br>(73 – 137) | 96<br>(69 – 125) |
| Belgium | 54<br>(37 – 75) | 57<br>(38 – 81) | 67<br>(46 – 91) |
| Belize | 8<br>(6 – 11) | 13<br>(10 – 17) | 19<br>(14 – 24) |
| Benin | 2,522<br>(1,731 – 4,086) | 8,041<br>(5,815 – 11,593) | 7,507<br>(5,346 – 11,241) |
| Bermuda | 1<br>(1 – 1) | 1<br>(1 – 1) | 1<br>(1 – 1) |
| Bhutan | 12<br>(8 – 18) | 9<br>(5 – 15) | 12<br>(7 – 18) |
| Bolivia | 162<br>(122 – 213) | 207<br>(154 – 271) | 199<br>(148 – 258) |
| Bosnia and Herzegovina | 41<br>(28 – 54) | 28<br>(19 – 38) | 23<br>(16 – 31) |

| Country | 1990 | 2005 | 2017 |
|---|---|---|---|
| Botswana | 214<br>(171 – 278) | 192<br>(156 – 238) | 196<br>(157 – 244) |
| Brazil | 3,152<br>(2,383 – 4,074) | 3,792<br>(2,816 – 4,925) | 3,778<br>(2,846 – 4,887) |
| Brunei | 2<br>(1 – 3) | 3<br>(2 – 4) | 3<br>(2 – 4) |
| Bulgaria | 65<br>(47 – 89) | 52<br>(36 – 71) | 47<br>(32 – 64) |
| Burkina Faso | 11,380<br>(9,069 – 14,785) | 36,901<br>(30,848 – 44,090) | 25,472<br>(20,578 – 30,930) |
| Burundi | 1,673<br>(920 – 3,947) | 1,920<br>(1,036 – 4,331) | 1,430<br>(724 – 3,706) |
| Cambodia | 281<br>(212 – 368) | 340<br>(249 – 449) | 293<br>(216 – 387) |
| Cameroon | 6,095<br>(4,200 – 9,649) | 28,753<br>(22,307 – 37,678) | 15,130<br>(10,643 – 22,727) |
| Canada | 73<br>(43 – 119) | 98<br>(59 – 149) | 97<br>(55 – 149) |
| Cape Verde | 121<br>(84 – 202) | 79<br>(55 – 122) | 48<br>(33 – 71) |
| Central African Republic | 927<br>(736 – 1,241) | 3,510<br>(2,822 – 4,451) | 1,493<br>(1,203 – 1,995) |
| Chad | 3,995<br>(2,826 – 6,270) | 7,003<br>(4,940 – 10,689) | 7,735<br>(5,395 – 12,377) |
| Chile | 85<br>(54 – 124) | 113<br>(71 – 159) | 123<br>(83 – 171) |
| China | 8,978<br>(5,987 – 12,569) | 9,813<br>(6,724 – 13,280) | 8,405<br>(5,640 – 11,661) |
| Colombia | 647<br>(482 – 851) | 860<br>(633 – 1,116) | 825<br>(601 – 1,066) |
| Comoros | 43<br>(21 – 123) | 52<br>(27 – 140) | 40<br>(16 – 108) |
| Congo | 602<br>(483 – 799) | 1,384<br>(1,111 – 1,836) | 885<br>(717 – 1,160) |
| Costa Rica | 54<br>(39 – 72) | 66<br>(47 – 86) | 61<br>(44 – 80) |
| Cote d'Ivoire | 10,558<br>(7,613 – 14,970) | 29,180<br>(22,052 – 37,443) | 17,775<br>(12,747 – 24,883) |
| Croatia | 34<br>(24 – 47) | 29<br>(20 – 39) | 29<br>(20 – 39) |
| Cuba | 209<br>(156 – 271) | 222<br>(167 – 286) | 184<br>(137 – 239) |
| Cyprus | 5<br>(3 – 6) | 5<br>(4 – 8) | 7<br>(4 – 9) |
| Czech Republic | 70<br>(47 – 96) | 65<br>(44 – 89) | 70<br>(47 – 95) |
| Democratic Republic of the Congo | 31,584<br>(25,057 – 41,604) | 63,325<br>(50,501 – 79,051) | 32,423<br>(25,931 – 42,179) |

| Country | 1990 | 2005 | 2017 |
|---|---|---|---|
| Denmark | 96<br>(77 – 116) | 98<br>(79 – 117) | 93<br>(72 – 114) |
| Djibouti | 59<br>(31 – 142) | 71<br>(34 – 191) | 74<br>(36 – 165) |
| Dominica | 2<br>(1 – 3) | 2<br>(1 – 2) | 2<br>(1 – 2) |
| Dominican Republic | 265<br>(205 – 336) | 413<br>(322 – 523) | 436<br>(343 – 546) |
| Ecuador | 193<br>(140 – 260) | 250<br>(185 – 324) | 255<br>(187 – 332) |
| Egypt | 1,098<br>(815 – 1,445) | 1,376<br>(1,011 – 1,803) | 1,507<br>(1,090 – 2,023) |
| El Salvador | 170<br>(130 – 217) | 161<br>(121 – 209) | 134<br>(101 – 174) |
| Equatorial Guinea | 237<br>(188 – 320) | 480<br>(380 – 644) | 362<br>(288 – 472) |
| Eritrea | 391<br>(203 – 949) | 512<br>(239 – 1,597) | 483<br>(227 – 1,309) |
| Estonia | 18<br>(13 – 23) | 14<br>(10 – 18) | 14<br>(10 – 18) |
| Ethiopia | 328<br>(180 – 518) | 486<br>(265 – 742) | 536<br>(287 – 840) |
| Federated States of Micronesia | 6<br>(4 – 10) | 5<br>(3 – 9) | 3<br>(2 – 5) |
| Fiji | 25<br>(17 – 39) | 23<br>(15 – 37) | 20<br>(14 – 29) |
| Finland | 13<br>(7 – 21) | 14<br>(9 – 22) | 14<br>(8 – 22) |
| France | 323<br>(212 – 461) | 342<br>(226 – 473) | 370<br>(241 – 512) |
| Gabon | 265<br>(212 – 355) | 169<br>(136 – 213) | 114<br>(91 – 148) |
| Georgia | 81<br>(59 – 105) | 60<br>(44 – 78) | 48<br>(36 – 62) |
| Germany | 434<br>(294 – 607) | 447<br>(297 – 621) | 462<br>(310 – 637) |
| Ghana | 4,958<br>(3,882 – 7,023) | 12,000<br>(10,228 – 14,023) | 9,148<br>(7,277 – 11,647) |
| Greece | 58<br>(39 – 81) | 59<br>(39 – 83) | 59<br>(39 – 81) |
| Greenland | 0<br>(0 – <1) | 0<br>(0 – <1) | 0<br>(0 – <1) |
| Grenada | 3<br>(2 – 3) | 3<br>(2 – 3) | 2<br>(2 – 3) |
| Guam | 2<br>(2 – 4) | 3<br>(2 – 6) | 3<br>(2 – 5) |
| Guatemala | 251<br>(192 – 325) | 372<br>(278 – 492) | 402<br>(301 – 516) |

| Country | 1990 | 2005 | 2017 |
|---|---|---|---|
| Guinea | 3,621<br>(2,517 – 5,741) | 13,528<br>(10,268 – 17,671) | 10,618<br>(7,566 – 15,016) |
| Guinea-Bissau | 444<br>(339 – 636) | 472<br>(360 – 681) | 410<br>(312 – 552) |
| Guyana | 25<br>(19 – 32) | 29<br>(23 – 36) | 27<br>(21 – 34) |
| Haiti | 372<br>(289 – 468) | 636<br>(489 – 813) | 815<br>(647 – 1,002) |
| Honduras | 158<br>(118 – 207) | 228<br>(174 – 293) | 260<br>(199 – 336) |
| Hungary | 76<br>(53 – 102) | 66<br>(46 – 90) | 63<br>(42 – 84) |
| Iceland | 2<br>(1 – 2) | 2<br>(1 – 2) | 2<br>(1 – 3) |
| India | 18,101<br>(13,613 – 23,605) | 23,435<br>(17,756 – 30,437) | 30,919<br>(23,491 – 39,334) |
| Indonesia | 3,237<br>(2,389 – 4,265) | 4,266<br>(3,165 – 5,601) | 4,212<br>(3,101 – 5,492) |
| Iran | 1,123<br>(829 – 1,499) | 1,208<br>(862 – 1,598) | 1,227<br>(893 – 1,602) |
| Iraq | 440<br>(326 – 581) | 839<br>(628 – 1,105) | 1,411<br>(1,093 – 1,800) |
| Ireland | 22<br>(15 – 31) | 23<br>(15 – 32) | 27<br>(18 – 38) |
| Israel | 155<br>(126 – 191) | 130<br>(113 – 149) | 265<br>(212 – 322) |
| Italy | 324<br>(220 – 454) | 325<br>(219 – 449) | 358<br>(235 – 494) |
| Jamaica | 59<br>(45 – 76) | 66<br>(49 – 85) | 62<br>(46 – 80) |
| Japan | 1,079<br>(748 – 1,444) | 1,023<br>(728 – 1,363) | 895<br>(618 – 1,209) |
| Jordan | 73<br>(51 – 98) | 106<br>(77 – 138) | 151<br>(104 – 200) |
| Kazakhstan | 244<br>(176 – 320) | 186<br>(132 – 243) | 232<br>(174 – 302) |
| Kenya | 24,697<br>(19,644 – 31,463) | 30,597<br>(24,832 – 38,050) | 27,323<br>(21,767 – 35,036) |
| Kiribati | 6<br>(4 – 10) | 8<br>(6 – 14) | 7<br>(5 – 10) |
| Kuwait | 19<br>(13 – 26) | 31<br>(22 – 40) | 47<br>(32 – 65) |
| Kyrgyzstan | 95<br>(69 – 126) | 92<br>(67 – 119) | 100<br>(72 – 134) |
| Laos | 102<br>(77 – 133) | 146<br>(106 – 192) | 134<br>(99 – 173) |
| Latvia | 33<br>(24 – 42) | 24<br>(17 – 31) | 21<br>(16 – 28) |

| Country | 1990 | 2005 | 2017 |
|---|---|---|---|
| Lebanon | 69<br>(50 – 92) | 164<br>(123 – 212) | 163<br>(120 – 212) |
| Lesotho | 366<br>(294 – 473) | 331<br>(270 – 419) | 271<br>(218 – 348) |
| Liberia | 867<br>(612 – 1,383) | 4,437<br>(3,414 – 5,756) | 2,621<br>(1,866 – 3,980) |
| Libya | 57<br>(39 – 80) | 86<br>(61 – 116) | 89<br>(62 – 117) |
| Lithuania | 45<br>(33 – 59) | 36<br>(26 – 47) | 30<br>(22 – 38) |
| Luxembourg | 2<br>(1 – 3) | 3<br>(2 – 4) | 3<br>(2 – 4) |
| Macedonia | 14<br>(10 – 20) | 14<br>(9 – 19) | 14<br>(10 – 19) |
| Madagascar | 44<br>(24 – 69) | 69<br>(36 – 110) | 90<br>(51 – 143) |
| Malawi | 16,959<br>(13,557 – 20,995) | 12,705<br>(11,535 – 14,005) | 5,307<br>(4,439 – 6,307) |
| Malaysia | 195<br>(131 – 276) | 310<br>(214 – 415) | 293<br>(199 – 396) |
| Maldives | 7<br>(6 – 10) | 10<br>(7 – 13) | 10<br>(7 – 13) |
| Mali | 8,512<br>(6,138 – 11,956) | 23,092<br>(18,024 – 29,624) | 24,636<br>(18,367 – 33,242) |
| Malta | 2<br>(1 – 3) | 2<br>(1 – 3) | 2<br>(2 – 3) |
| Marshall Islands | 3<br>(2 – 5) | 3<br>(2 – 5) | 2<br>(1 – 3) |
| Mauritania | 693<br>(482 – 1,102) | 1,110<br>(777 – 1,711) | 809<br>(548 – 1,366) |
| Mauritius | 11<br>(8 – 15) | 15<br>(10 – 19) | 12<br>(8 – 16) |
| Mexico | 2,027<br>(1,517 – 2,630) | 2,181<br>(1,643 – 2,819) | 2,211<br>(1,645 – 2,881) |
| Moldova | 94<br>(70 – 122) | 92<br>(71 – 116) | 63<br>(47 – 80) |
| Mongolia | 40<br>(29 – 53) | 43<br>(31 – 57) | 67<br>(51 – 87) |
| Montenegro | 5<br>(4 – 7) | 5<br>(3 – 6) | 4<br>(3 – 6) |
| Morocco | 862<br>(667 – 1,097) | 1,008<br>(763 – 1,314) | 737<br>(550 – 963) |
| Mozambique | 11,385<br>(9,034 – 14,724) | 16,600<br>(14,159 – 19,289) | 7,328<br>(6,086 – 8,896) |
| Myanmar | 1,128<br>(853 – 1,453) | 1,407<br>(1,037 – 1,885) | 1,111<br>(835 – 1,431) |
| Namibia | 375<br>(297 – 485) | 370<br>(299 – 469) | 259<br>(209 – 332) |

| Country | 1990 | 2005 | 2017 |
|---|---|---|---|
| Nepal | 935<br>(565 – 1,483) | 1,091<br>(690 – 1,659) | 978<br>(666 – 1,360) |
| Netherlands | 82<br>(55 – 114) | 91<br>(64 – 125) | 96<br>(63 – 134) |
| New Zealand | 27<br>(18 – 37) | 34<br>(23 – 46) | 38<br>(26 – 52) |
| Nicaragua | 128<br>(96 – 170) | 187<br>(144 – 241) | 188<br>(146 – 239) |
| Niger | 5,873<br>(4,062 – 8,847) | 16,967<br>(12,341 – 22,710) | 18,817<br>(13,287 – 28,030) |
| Nigeria | 73,073<br>(53,872 – 101,157) | 212,428<br>(160,678 – 278,877) | 161,933<br>(115,120 – 230,157) |
| North Korea | 590<br>(245 – 1,107) | 716<br>(399 – 1,177) | 636<br>(350 – 1,010) |
| Northern Mariana Islands | 1<br>(1 – 1) | 1<br>(1 – 2) | 1<br>(0 – 1) |
| Norway | 34<br>(25 – 45) | 37<br>(27 – 48) | 42<br>(30 – 54) |
| Oman | 97<br>(75 – 126) | 91<br>(69 – 118) | 70<br>(50 – 94) |
| Pakistan | 6,305<br>(4,357 – 9,123) | 7,279<br>(5,155 – 9,954) | 10,258<br>(6,580 – 15,050) |
| Palestine | 52<br>(39 – 68) | 103<br>(77 – 131) | 184<br>(143 – 234) |
| Panama | 41<br>(30 – 54) | 59<br>(43 – 77) | 67<br>(49 – 88) |
| Papua New Guinea | 358<br>(261 – 564) | 728<br>(496 – 1,339) | 729<br>(519 – 1,078) |
| Paraguay | 114<br>(85 – 151) | 132<br>(97 – 173) | 127<br>(94 – 168) |
| Peru | 353<br>(252 – 471) | 460<br>(331 – 615) | 509<br>(373 – 669) |
| Philippines | 1,536<br>(1,150 – 2,002) | 2,293<br>(1,701 – 3,000) | 2,022<br>(1,495 – 2,646) |
| Poland | 293<br>(203 – 400) | 182<br>(127 – 240) | 248<br>(171 – 339) |
| Portugal | 60<br>(41 – 82) | 59<br>(41 – 81) | 61<br>(42 – 85) |
| Puerto Rico | 48<br>(34 – 63) | 52<br>(39 – 68) | 48<br>(35 – 63) |
| Qatar | 5<br>(3 – 6) | 11<br>(7 – 15) | 30<br>(20 – 43) |
| Romania | 260<br>(186 – 340) | 165<br>(115 – 217) | 135<br>(92 – 184) |
| Russian Federation | 2,275<br>(1,679 – 2,966) | 1,955<br>(1,443 – 2,552) | 1,967<br>(1,469 – 2,541) |
| Rwanda | 1,138<br>(597 – 2,886) | 1,091<br>(552 – 2,994) | 835<br>(400 – 2,100) |

| Country | 1990 | 2005 | 2017 |
|---|---|---|---|
| Saint Lucia | 4<br>(3 – 5) | 4<br>(3 – 5) | 3<br>(3 – 4) |
| Saint Vincent and the Grenadines | 3<br>(3 – 4) | 3<br>(2 – 4) | 3<br>(2 – 3) |
| Samoa | 7<br>(5 – 13) | 9<br>(6 – 15) | 7<br>(5 – 11) |
| Sao Tome and Principe | 37<br>(26 – 64) | 43<br>(30 – 66) | 34<br>(24 – 53) |
| Saudi Arabia | 488<br>(373 – 629) | 657<br>(497 – 859) | 527<br>(377 – 705) |
| Senegal | 3,048<br>(2,302 – 4,476) | 3,127<br>(2,360 – 4,600) | 1,823<br>(1,418 – 2,459) |
| Serbia | 87<br>(61 – 114) | 79<br>(56 – 105) | 65<br>(47 – 87) |
| Seychelles | 1<br>(1 – 1) | 1<br>(1 – 1) | 1<br>(1 – 1) |
| Sierra Leone | 2,774<br>(1,972 – 4,063) | 10,836<br>(8,405 – 13,402) | 6,332<br>(4,547 – 8,949) |
| Singapore | 23<br>(15 – 33) | 31<br>(20 – 44) | 32<br>(20 – 46) |
| Slovakia | 38<br>(26 – 52) | 35<br>(24 – 49) | 35<br>(24 – 48) |
| Slovenia | 14<br>(9 – 19) | 13<br>(9 – 17) | 14<br>(9 – 19) |
| Solomon Islands | 25<br>(17 – 42) | 43<br>(29 – 81) | 37<br>(26 – 55) |
| Somalia | 1,140<br>(575 – 2,953) | 2,005<br>(996 – 4,722) | 1,680<br>(819 – 4,373) |
| South Africa | 5,956<br>(3,838 – 9,897) | 4,553<br>(3,244 – 7,401) | 3,315<br>(2,450 – 5,261) |
| South Korea | 347<br>(221 – 486) | 319<br>(204 – 448) | 296<br>(188 – 427) |
| South Sudan | 1,147<br>(604 – 2,689) | 2,251<br>(1,225 – 4,735) | 1,367<br>(683 – 3,761) |
| Spain | 215<br>(141 – 309) | 238<br>(160 – 331) | 260<br>(171 – 365) |
| Sri Lanka | 357<br>(259 – 464) | 341<br>(252 – 451) | 269<br>(190 – 354) |
| Sudan | 744<br>(576 – 958) | 2,548<br>(2,000 – 3,180) | 2,221<br>(1,732 – 2,826) |
| Suriname | 11<br>(8 – 15) | 15<br>(12 – 20) | 15<br>(11 – 19) |
| Swaziland | 162<br>(128 – 213) | 155<br>(124 – 201) | 127<br>(101 – 161) |
| Sweden | 70<br>(50 – 92) | 69<br>(50 – 90) | 82<br>(60 – 107) |
| Switzerland | 39<br>(27 – 53) | 41<br>(27 – 57) | 49<br>(33 – 66) |

| Country | 1990 | 2005 | 2017 |
|---|---|---|---|
| Syria | 300<br>(220 – 393) | 473<br>(352 – 622) | 303<br>(218 – 405) |
| Taiwan | 1,036<br>(791 – 1,330) | 709<br>(550 – 878) | 749<br>(577 – 935) |
| Tajikistan | 108<br>(80 – 142) | 116<br>(84 – 156) | 147<br>(105 – 194) |
| Tanzania | 4,967<br>(3,877 – 6,854) | 6,677<br>(5,344 – 8,456) | 3,217<br>(2,503 – 4,263) |
| Thailand | 1,406<br>(1,059 – 1,790) | 1,554<br>(1,181 – 2,008) | 1,267<br>(952 – 1,656) |
| The Bahamas | 4<br>(3 – 6) | 6<br>(4 – 8) | 7<br>(5 – 8) |
| The Gambia | 821<br>(635 – 1,107) | 990<br>(805 – 1,238) | 717<br>(561 – 979) |
| Timor-Leste | 13<br>(10 – 18) | 20<br>(14 – 27) | 20<br>(14 – 27) |
| Togo | 2,884<br>(2,085 – 4,250) | 7,822<br>(5,800 – 10,193) | 7,856<br>(5,855 – 10,278) |
| Tonga | 5<br>(4 – 8) | 6<br>(4 – 10) | 4<br>(3 – 6) |
| Trinidad and Tobago | 26<br>(19 – 34) | 25<br>(19 – 33) | 25<br>(18 – 32) |
| Tunisia | 175<br>(130 – 235) | 206<br>(155 – 265) | 202<br>(149 – 261) |
| Turkey | 1,233<br>(915 – 1,640) | 1,787<br>(1,341 – 2,297) | 1,377<br>(1,022 – 1,784) |
| Turkmenistan | 96<br>(72 – 124) | 74<br>(54 – 98) | 74<br>(55 – 96) |
| Uganda | 6,495<br>(3,534 – 13,943) | 8,616<br>(4,650 – 17,397) | 3,307<br>(1,518 – 9,023) |
| Ukraine | 767<br>(559 – 984) | 674<br>(494 – 867) | 628<br>(464 – 799) |
| United Arab Emirates | 30<br>(21 – 40) | 56<br>(38 – 76) | 107<br>(67 – 159) |
| United Kingdom | 449<br>(328 – 587) | 457<br>(332 – 604) | 509<br>(374 – 668) |
| United States | 4,552<br>(3,444 – 5,747) | 5,133<br>(3,915 – 6,443) | 5,981<br>(4,539 – 7,512) |
| Uruguay | 19<br>(12 – 27) | 23<br>(16 – 32) | 23<br>(14 – 33) |
| Uzbekistan | 303<br>(215 – 407) | 303<br>(208 – 403) | 355<br>(245 – 473) |
| Vanuatu | 12<br>(9 – 19) | 21<br>(14 – 39) | 19<br>(13 – 29) |
| Venezuela | 319<br>(231 – 423) | 418<br>(304 – 558) | 442<br>(322 – 581) |
| Vietnam | 940<br>(650 – 1,257) | 1,146<br>(813 – 1,528) | 1,039<br>(743 – 1,366) |

| Country | 1990 | 2005 | 2017 |
|---|---|---|---|
| Virgin Islands, U.S. | 1<br>(1 – 2) | 2<br>(1 – 2) | 1<br>(1 – 2) |
| Yemen | 715<br>(554 – 903) | 1,771<br>(1,388 – 2,227) | 2,003<br>(1,561 – 2,500) |
| Zambia | 1,252<br>(636 – 2,803) | 1,721<br>(884 – 4,388) | 1,667<br>(787 – 4,399) |
| Zimbabwe | 1,822<br>(1,456 – 2,405) | 2,054<br>(1,641 – 2,735) | 2,445<br>(1,954 – 3,192) |

**Table S6: The numbers of iNTS cases, deaths, and DALYs, with 95% uncertainty intervals, by country, in 2017.**

| Country | Cases | Deaths | DALYs |
|---|---|---|---|
| Afghanistan | 2,837 (2,247 – 3,613) | 579 (321 – 944) | 41,408 (23,115 – 68,167) |
| Albania | 24 (17 – 31) | 3 (1 – 5) | 105 (54 – 179) |
| Algeria | 609 (443 – 794) | 39 (20 – 69) | 2,271 (1,118 – 4,198) |
| American Samoa | 1 (1 – 2) | 0 (0 – <1) | 4 (2 – 8) |
| Andorra | 0 (0 – 1) | 0 (0 – <1) | 1 (0 – 2) |
| Angola | 5,533 (4,389 – 7,322) | 548 (303 – 894) | 39,238 (21,523 – 66,201) |
| Antigua and Barbuda | 2 (1 – 2) | 0 (0 – <1) | 6 (3 – 10) |
| Argentina | 341 (226 – 466) | 20 (10 – 36) | 1,065 (526 – 1,897) |
| Armenia | 31 (22 – 41) | 2 (1 – 4) | 111 (58 – 194) |
| Australia | 150 (97 – 211) | 8 (4 – 15) | 330 (151 – 629) |
| Austria | 50 (32 – 69) | 4 (2 – 8) | 100 (47 – 188) |
| Azerbaijan | 125 (91 – 164) | 9 (5 – 15) | 469 (240 – 843) |
| Bahrain | 18 (13 – 25) | 1 (0 – 2) | 52 (26 – 93) |
| Bangladesh | 6,696 (4,715 – 9,484) | 752 (403 – 1,305) | 51,116 (26,691 – 92,812) |
| Barbados | 4 (3 – 5) | 0 (0 – <1) | 11 (6 – 20) |
| Belarus | 96 (69 – 125) | 7 (4 – 12) | 258 (135 – 451) |
| Belgium | 67 (46 – 91) | 5 (2 – 9) | 131 (59 – 248) |
| Belize | 19 (14 – 24) | 1 (1 – 2) | 77 (42 – 132) |
| Benin | 7,507 (5,346 – 11,241) | 1,023 (531 – 1,856) | 76,560 (39,280 – 143,762) |
| Bermuda | 1 (1 – 1) | 0 (0 – <1) | 3 (1 – 5) |
| Bhutan | 12 (7 – 18) | 1 (0 – 2) | 64 (30 – 127) |
| Bolivia | 199 (148 – 258) | 17 (9 – 29) | 1,026 (508 – 1,801) |
| Bosnia and Herzegovina | 23 (16 – 31) | 2 (1 – 4) | 84 (42 – 142) |

| Country | Cases | Deaths | DALYs |
|---|---|---|---|
| Botswana | 196<br>(157 – 244) | 6<br>(3 – 11) | 422<br>(218 – 777) |
| Brazil | 3,778<br>(2,846 – 4,887) | 249<br>(135 – 416) | 14,485<br>(7,718 – 24,859) |
| Brunei | 3<br>(2 – 4) | 0<br>(0 – <1) | 7<br>(3 – 15) |
| Bulgaria | 47<br>(32 – 64) | 5<br>(2 – 8) | 140<br>(72 – 240) |
| Burkina Faso | 25,472<br>(20,578 – 30,930) | 4,217<br>(2,336 – 6,795) | 319,627<br>(176,339 – 521,278) |
| Burundi | 1,430<br>(724 – 3,706) | 171<br>(71 – 473) | 13,123<br>(5,176 – 38,886) |
| Cambodia | 293<br>(216 – 387) | 23<br>(12 – 39) | 1,396<br>(769 – 2,407) |
| Cameroon | 15,130<br>(10,643 – 22,727) | 1,333<br>(676 – 2,467) | 99,196<br>(48,046 – 183,937) |
| Canada | 97<br>(55 – 149) | 5<br>(2 – 10) | 183<br>(77 – 353) |
| Cape Verde | 48<br>(33 – 71) | 5<br>(3 – 9) | 345<br>(181 – 659) |
| Central African Republic | 1,493<br>(1,203 – 1,995) | 178<br>(97 – 310) | 12,642<br>(6,760 – 23,112) |
| Chad | 7,735<br>(5,395 – 12,377) | 1,240<br>(648 – 2,243) | 95,445<br>(49,375 – 178,712) |
| Chile | 123<br>(83 – 171) | 7<br>(3 – 13) | 341<br>(166 – 640) |
| China | 8,405<br>(5,640 – 11,661) | 586<br>(294 – 1,001) | 26,852<br>(13,699 – 47,233) |
| Colombia | 825<br>(601 – 1,066) | 59<br>(31 – 100) | 3,389<br>(1,743 – 5,964) |
| Comoros | 40<br>(16 – 108) | 5<br>(2 – 14) | 372<br>(116 – 1,100) |
| Congo | 885<br>(717 – 1,160) | 65<br>(35 – 112) | 4,312<br>(2,224 – 7,544) |
| Costa Rica | 61<br>(44 – 80) | 4<br>(2 – 6) | 207<br>(109 – 362) |
| Cote d'Ivoire | 17,775<br>(12,747 – 24,883) | 2,139<br>(1,183 – 3,723) | 160,812<br>(86,817 – 288,183) |
| Croatia | 29<br>(20 – 39) | 2<br>(1 – 4) | 70<br>(34 – 124) |
| Cuba | 184<br>(137 – 239) | 14<br>(7 – 23) | 651<br>(345 – 1,123) |
| Cyprus | 7<br>(4 – 9) | 1<br>(0 – 1) | 17<br>(8 – 31) |
| Czech Republic | 70<br>(47 – 95) | 5<br>(2 – 10) | 167<br>(83 – 295) |
| Democratic Republic of the Congo | 32,423<br>(25,931 – 42,179) | 3,897<br>(2,126 – 6,491) | 270,866<br>(144,590 – 467,116) |

| Country | Cases | Deaths | DALYs |
|---|---|---|---|
| Denmark | 93<br>(72 – 114) | 9<br>(4 – 17) | 201<br>(97 – 349) |
| Djibouti | 74<br>(36 – 165) | 7<br>(3 – 18) | 511<br>(196 – 1,390) |
| Dominica | 2<br>(1 – 2) | 0<br>(0 – <1) | 7<br>(4 – 12) |
| Dominican Republic | 436<br>(343 – 546) | 40<br>(22 – 68) | 2,532<br>(1,401 – 4,358) |
| Ecuador | 255<br>(187 – 332) | 19<br>(10 – 33) | 1,151<br>(595 – 2,022) |
| Egypt | 1,507<br>(1,090 – 2,023) | 126<br>(67 – 212) | 8,215<br>(4,370 – 14,347) |
| El Salvador | 134<br>(101 – 174) | 11<br>(6 – 18) | 619<br>(321 – 1,077) |
| Equatorial Guinea | 362<br>(288 – 472) | 17<br>(8 – 33) | 1,255<br>(597 – 2,433) |
| Eritrea | 483<br>(227 – 1,309) | 89<br>(35 – 260) | 6,638<br>(2,467 – 20,918) |
| Estonia | 14<br>(10 – 18) | 1<br>(0 – 2) | 31<br>(16 – 57) |
| Ethiopia | 536<br>(287 – 840) | 74<br>(35 – 136) | 5,066<br>(2,192 – 9,472) |
| Federated States of Micronesia | 3<br>(2 – 5) | 0<br>(0 – <1) | 20<br>(9 – 36) |
| Fiji | 20<br>(14 – 29) | 2<br>(1 – 3) | 116<br>(59 – 215) |
| Finland | 14<br>(8 – 22) | 1<br>(0 – 2) | 29<br>(12 – 57) |
| France | 370<br>(241 – 512) | 31<br>(12 – 61) | 793<br>(373 – 1,526) |
| Gabon | 114<br>(91 – 148) | 7<br>(4 – 12) | 436<br>(225 – 794) |
| Georgia | 48<br>(36 – 62) | 4<br>(2 – 7) | 183<br>(96 – 312) |
| Germany | 462<br>(310 – 637) | 41<br>(16 – 80) | 995<br>(434 – 1,836) |
| Ghana | 9,148<br>(7,277 – 11,647) | 866<br>(494 – 1,429) | 62,687<br>(34,438 – 107,455) |
| Greece | 59<br>(39 – 81) | 7<br>(3 – 13) | 155<br>(75 – 277) |
| Greenland | 0<br>(0 – <1) | 0<br>(0 – <1) | 1<br>(0 – 1) |
| Grenada | 2<br>(2 – 3) | 0<br>(0 – <1) | 9<br>(4 – 15) |
| Guam | 3<br>(2 – 5) | 0<br>(0 – <1) | 9<br>(4 – 18) |
| Guatemala | 402<br>(301 – 516) | 36<br>(20 – 60) | 2,478<br>(1,326 – 4,106) |

| Country | Cases | Deaths | DALYs |
| --- | --- | --- | --- |
| Guinea | 10,618<br>(7,566 – 15,016) | 1,564<br>(824 – 2,693) | 116,097<br>(59,306 – 205,805) |
| Guinea-Bissau | 410<br>(312 – 552) | 57<br>(31 – 101) | 4,353<br>(2,274 – 7,834) |
| Guyana | 27<br>(21 – 34) | 2<br>(1 – 3) | 120<br>(66 – 209) |
| Haiti | 815<br>(647 – 1,002) | 77<br>(44 – 123) | 5,268<br>(3,028 – 8,555) |
| Honduras | 260<br>(199 – 336) | 29<br>(15 – 49) | 1,780<br>(906 – 3,163) |
| Hungary | 63<br>(42 – 84) | 5<br>(2 – 9) | 154<br>(78 – 271) |
| Iceland | 2<br>(1 – 3) | 0<br>(0 – <1) | 4<br>(2 – 7) |
| India | 30,919<br>(23,491 – 39,334) | 2,349<br>(1,297 – 3,821) | 138,869<br>(76,561 – 226,330) |
| Indonesia | 4,212<br>(3,101 – 5,492) | 290<br>(154 – 489) | 16,666<br>(9,123 – 28,401) |
| Iran | 1,227<br>(893 – 1,602) | 85<br>(44 – 150) | 4,875<br>(2,499 – 8,750) |
| Iraq | 1,411<br>(1,093 – 1,800) | 96<br>(53 – 162) | 6,593<br>(3,614 – 11,323) |
| Ireland | 27<br>(18 – 38) | 2<br>(1 – 4) | 58<br>(26 – 111) |
| Israel | 265<br>(212 – 322) | 23<br>(11 – 41) | 707<br>(376 – 1,239) |
| Italy | 358<br>(235 – 494) | 35<br>(13 – 68) | 786<br>(352 – 1,461) |
| Jamaica | 62<br>(46 – 80) | 4<br>(2 – 7) | 247<br>(129 – 439) |
| Japan | 895<br>(618 – 1,209) | 66<br>(29 – 123) | 2,031<br>(1,006 – 3,651) |
| Jordan | 151<br>(104 – 200) | 10<br>(5 – 18) | 661<br>(337 – 1,215) |
| Kazakhstan | 232<br>(174 – 302) | 15<br>(8 – 25) | 837<br>(431 – 1,498) |
| Kenya | 27,323<br>(21,767 – 35,036) | 2,466<br>(1,446 – 3,900) | 176,829<br>(103,381 – 285,558) |
| Kiribati | 7<br>(5 – 10) | 1<br>(0 – 2) | 66<br>(36 – 124) |
| Kuwait | 47<br>(32 – 65) | 2<br>(1 – 4) | 121<br>(58 – 229) |
| Kyrgyzstan | 100<br>(72 – 134) | 8<br>(4 – 14) | 510<br>(271 – 880) |
| Laos | 134<br>(99 – 173) | 12<br>(7 – 20) | 830<br>(453 – 1,404) |
| Latvia | 21<br>(16 – 28) | 2<br>(1 – 3) | 54<br>(27 – 95) |

| Country | Cases | Deaths | DALYs |
|---|---|---|---|
| Lebanon | 163<br>(120 – 212) | 10<br>(5 – 19) | 596<br>(280 – 1,148) |
| Lesotho | 271<br>(218 – 348) | 11<br>(6 – 19) | 764<br>(399 – 1,303) |
| Liberia | 2,621<br>(1,866 – 3,980) | 326<br>(162 – 595) | 23,772<br>(11,216 – 45,067) |
| Libya | 89<br>(62 – 117) | 7<br>(3 – 13) | 427<br>(203 – 825) |
| Lithuania | 30<br>(22 – 38) | 2<br>(1 – 4) | 69<br>(35 – 121) |
| Luxembourg | 3<br>(2 – 4) | 0<br>(0 – <1) | 7<br>(3 – 13) |
| Macedonia | 14<br>(10 – 19) | 1<br>(1 – 2) | 52<br>(26 – 90) |
| Madagascar | 90<br>(51 – 143) | 11<br>(5 – 21) | 713<br>(314 – 1,382) |
| Malawi | 5,307<br>(4,439 – 6,307) | 609<br>(338 – 1,024) | 44,876<br>(24,960 – 75,955) |
| Malaysia | 293<br>(199 – 396) | 13<br>(6 – 23) | 716<br>(333 – 1,372) |
| Maldives | 10<br>(7 – 13) | 1<br>(0 – 1) | 35<br>(18 – 61) |
| Mali | 24,636<br>(18,367 – 33,242) | 5,128<br>(2,763 – 8,491) | 395,208<br>(205,981 – 673,495) |
| Malta | 2<br>(2 – 3) | 0<br>(0 – <1) | 7<br>(3 – 12) |
| Marshall Islands | 2<br>(1 – 3) | 0<br>(0 – <1) | 13<br>(6 – 23) |
| Mauritania | 809<br>(548 – 1,366) | 102<br>(54 – 183) | 7,165<br>(3,631 – 13,361) |
| Mauritius | 12<br>(8 – 16) | 1<br>(0 – 1) | 39<br>(20 – 69) |
| Mexico | 2,211<br>(1,645 – 2,881) | 144<br>(79 – 242) | 8,549<br>(4,625 – 14,461) |
| Moldova | 63<br>(47 – 80) | 5<br>(3 – 8) | 224<br>(122 – 374) |
| Mongolia | 67<br>(51 – 87) | 5<br>(3 – 8) | 297<br>(157 – 529) |
| Montenegro | 4<br>(3 – 6) | 0<br>(0 – 1) | 13<br>(7 – 23) |
| Morocco | 737<br>(550 – 963) | 74<br>(39 – 123) | 4,397<br>(2,274 – 7,520) |
| Mozambique | 7,328<br>(6,086 – 8,896) | 973<br>(542 – 1,584) | 76,075<br>(42,202 – 126,279) |
| Myanmar | 1,111<br>(835 – 1,431) | 106<br>(58 – 178) | 6,212<br>(3,429 – 10,497) |
| Namibia | 259<br>(209 – 332) | 12<br>(6 – 21) | 807<br>(416 – 1,515) |

| Country | Cases | Deaths | DALYs |
|---|---|---|---|
| Nepal | 978<br>(666 – 1,360) | 89<br>(46 – 156) | 5,588<br>(2,892 – 9,987) |
| Netherlands | 96<br>(63 – 134) | 7<br>(3 – 14) | 194<br>(86 – 358) |
| New Zealand | 38<br>(26 – 52) | 2<br>(1 – 4) | 93<br>(44 – 175) |
| Nicaragua | 188<br>(146 – 239) | 17<br>(9 – 27) | 1,058<br>(586 – 1,762) |
| Niger | 18,817<br>(13,287 – 28,030) | 3,055<br>(1,547 – 5,409) | 229,779<br>(112,077 – 425,454) |
| Nigeria | 161,933<br>(115,120 – 230,157) | 15,345<br>(8,122 – 27,297) | 1,157,878<br>(601,676 – 2,128,304) |
| North Korea | 636<br>(350 – 1,010) | 73<br>(33 – 135) | 3,815<br>(1,705 – 7,510) |
| Northern Mariana Islands | 1<br>(0 – 1) | 0<br>(0 – <1) | 2<br>(1 – 4) |
| Norway | 42<br>(30 – 54) | 3<br>(1 – 5) | 77<br>(38 – 143) |
| Oman | 70<br>(50 – 94) | 3<br>(2 – 6) | 221<br>(105 – 405) |
| Pakistan | 10,258<br>(6,580 – 15,050) | 1,312<br>(652 – 2,409) | 90,819<br>(44,410 – 171,126) |
| Palestine | 184<br>(143 – 234) | 17<br>(10 – 29) | 1,119<br>(609 – 1,898) |
| Panama | 67<br>(49 – 88) | 4<br>(2 – 7) | 264<br>(138 – 464) |
| Papua New Guinea | 729<br>(519 – 1,078) | 80<br>(44 – 142) | 6,044<br>(3,270 – 10,993) |
| Paraguay | 127<br>(94 – 168) | 7<br>(4 – 13) | 440<br>(225 – 787) |
| Peru | 509<br>(373 – 669) | 36<br>(19 – 63) | 2,112<br>(1,105 – 3,716) |
| Philippines | 2,022<br>(1,495 – 2,646) | 157<br>(87 – 268) | 10,110<br>(5,607 – 17,248) |
| Poland | 248<br>(171 – 339) | 19<br>(9 – 34) | 602<br>(299 – 1,101) |
| Portugal | 61<br>(42 – 85) | 5<br>(2 – 10) | 126<br>(59 – 231) |
| Puerto Rico | 48<br>(35 – 63) | 3<br>(2 – 6) | 133<br>(65 – 245) |
| Qatar | 30<br>(20 – 43) | 2<br>(1 – 4) | 124<br>(51 – 251) |
| Romania | 135<br>(92 – 184) | 13<br>(6 – 23) | 395<br>(207 – 694) |
| Russian Federation | 1,967<br>(1,469 – 2,541) | 121<br>(62 – 205) | 4,855<br>(2,573 – 8,416) |
| Rwanda | 835<br>(400 – 2,100) | 107<br>(41 – 303) | 8,233<br>(3,104 – 24,772) |

| Country | Cases | Deaths | DALYs |
|---|---|---|---|
| Saint Lucia | 3<br>(3 – 4) | 0<br>(0 – <1) | 13<br>(7 – 22) |
| Saint Vincent and the Grenadines | 3<br>(2 – 3) | 0<br>(0 – <1) | 14<br>(7 – 24) |
| Samoa | 7<br>(5 – 11) | 0<br>(0 – 1) | 34<br>(17 – 67) |
| Sao Tome and Principe | 34<br>(24 – 53) | 5<br>(3 – 10) | 367<br>(184 – 701) |
| Saudi Arabia | 527<br>(377 – 705) | 21<br>(10 – 39) | 1,229<br>(586 – 2,323) |
| Senegal | 1,823<br>(1,418 – 2,459) | 288<br>(163 – 484) | 20,510<br>(11,265 – 35,459) |
| Serbia | 65<br>(47 – 87) | 6<br>(3 – 11) | 194<br>(101 – 334) |
| Seychelles | 1<br>(1 – 1) | 0<br>(0 – <1) | 4<br>(2 – 6) |
| Sierra Leone | 6,332<br>(4,547 – 8,949) | 780<br>(419 – 1,362) | 56,889<br>(30,289 – 102,366) |
| Singapore | 32<br>(20 – 46) | 1<br>(1 – 3) | 65<br>(27 – 123) |
| Slovakia | 35<br>(24 – 48) | 3<br>(1 – 5) | 89<br>(45 – 157) |
| Slovenia | 14<br>(9 – 19) | 1<br>(0 – 2) | 31<br>(15 – 55) |
| Solomon Islands | 37<br>(26 – 55) | 4<br>(2 – 6) | 275<br>(147 – 484) |
| Somalia | 1,680<br>(819 – 4,373) | 395<br>(147 – 1,086) | 30,642<br>(10,850 – 88,796) |
| South Africa | 3,315<br>(2,450 – 5,261) | 139<br>(72 – 299) | 9,878<br>(4,902 – 22,291) |
| South Korea | 296<br>(188 – 427) | 16<br>(7 – 30) | 629<br>(288 – 1,180) |
| South Sudan | 1,367<br>(683 – 3,761) | 227<br>(88 – 680) | 17,478<br>(6,432 – 55,846) |
| Spain | 260<br>(171 – 365) | 21<br>(8 – 43) | 526<br>(233 – 1,008) |
| Sri Lanka | 269<br>(190 – 354) | 17<br>(9 – 29) | 837<br>(442 – 1,460) |
| Sudan | 2,221<br>(1,732 – 2,826) | 215<br>(120 – 357) | 14,968<br>(8,295 – 25,156) |
| Suriname | 15<br>(11 – 19) | 1<br>(1 – 2) | 54<br>(29 – 89) |
| Swaziland | 127<br>(101 – 161) | 5<br>(3 – 9) | 367<br>(193 – 635) |
| Sweden | 82<br>(60 – 107) | 7<br>(3 – 12) | 200<br>(97 – 359) |
| Switzerland | 49<br>(33 – 66) | 3<br>(1 – 7) | 94<br>(42 – 186) |

| Country | Cases | Deaths | DALYs |
|---|---|---|---|
| Syria | 303<br>(218 – 405) | 26<br>(13 – 46) | 1,605<br>(817 – 2,873) |
| Taiwan | 749<br>(577 – 935) | 44<br>(22 – 75) | 1,751<br>(897 – 3,056) |
| Tajikistan | 147<br>(105 – 194) | 16<br>(9 – 27) | 1,047<br>(542 – 1,782) |
| Tanzania | 3,217<br>(2,503 – 4,263) | 458<br>(248 – 778) | 34,922<br>(19,050 – 60,687) |
| Thailand | 1,267<br>(952 – 1,656) | 87<br>(46 – 147) | 4,265<br>(2,179 – 7,421) |
| The Bahamas | 7<br>(5 – 8) | 0<br>(0 – 1) | 17<br>(9 – 32) |
| The Gambia | 717<br>(561 – 979) | 100<br>(54 – 174) | 7,314<br>(3,891 – 13,107) |
| Timor-Leste | 20<br>(14 – 27) | 2<br>(1 – 3) | 135<br>(66 – 234) |
| Togo | 7,856<br>(5,855 – 10,278) | 735<br>(403 – 1,242) | 52,294<br>(28,117 – 89,593) |
| Tonga | 4<br>(3 – 6) | 0<br>(0 – <1) | 17<br>(9 – 31) |
| Trinidad and Tobago | 25<br>(18 – 32) | 2<br>(1 – 3) | 85<br>(43 – 148) |
| Tunisia | 202<br>(149 – 261) | 12<br>(7 – 21) | 640<br>(335 – 1,164) |
| Turkey | 1,377<br>(1,022 – 1,784) | 82<br>(43 – 141) | 4,398<br>(2,284 – 7,849) |
| Turkmenistan | 74<br>(55 – 96) | 6<br>(3 – 9) | 327<br>(171 – 566) |
| Uganda | 3,307<br>(1,518 – 9,023) | 339<br>(127 – 1,040) | 27,076<br>(9,751 – 86,684) |
| Ukraine | 628<br>(464 – 799) | 86<br>(45 – 148) | 4,064<br>(2,092 – 7,120) |
| United Arab Emirates | 107<br>(67 – 159) | 6<br>(3 – 11) | 349<br>(169 – 657) |
| United Kingdom | 509<br>(374 – 668) | 37<br>(17 – 68) | 1,154<br>(575 – 2,036) |
| United States | 5,981<br>(4,539 – 7,512) | 334<br>(155 – 608) | 10,979<br>(5,447 – 19,874) |
| Uruguay | 23<br>(14 – 33) | 1<br>(1 – 3) | 67<br>(31 – 125) |
| Uzbekistan | 355<br>(245 – 473) | 27<br>(14 – 47) | 1,574<br>(844 – 2,773) |
| Vanuatu | 19<br>(13 – 29) | 3<br>(1 – 5) | 188<br>(97 – 328) |
| Venezuela | 442<br>(322 – 581) | 29<br>(15 – 49) | 1,743<br>(925 – 3,074) |
| Vietnam | 1,039<br>(743 – 1,366) | 75<br>(39 – 126) | 4,008<br>(2,131 – 6,913) |

| Country | Cases | Deaths | DALYs |
|---|---|---|---|
| Virgin Islands, U.S. | 1<br>(1 – 2) | 0<br>(0 – <1) | 5<br>(3 – 9) |
| Yemen | 2,003<br>(1,561 – 2,500) | 214<br>(118 – 364) | 14,728<br>(8,135 – 25,091) |
| Zambia | 1,667<br>(787 – 4,399) | 163<br>(62 – 524) | 13,108<br>(4,767 – 43,886) |
| Zimbabwe | 2,445<br>(1,954 – 3,192) | 151<br>(83 – 252) | 11,171<br>(6,162 – 18,951) |

**Table S7: iNTS incidence rates and mortality rates, by country, with 95% uncertainty intervals, in 1990 and 2017.**

| Country | Incidence (per 100,000) | | Mortality (per million) | |
|---|---|---|---|---|
| | 1990 | 2017 | 1990 | 2017 |
| Afghanistan | 3.9 (3.0, 4.8) | 6.9 (5.5, 8.4) | 8.2 (2.8, 14.5) | 16.8 (9.3, 26.8) |
| Albania | 1.3 (0.9, 1.6) | 0.93 (0.65, 1.23) | 1.4 (0.8, 2.2) | 0.88 (0.45, 1.48) |
| Algeria | 1.5 (1.1, 2.0) | 1.5 (1.1, 2.0) | 1.6 (0.9, 2.6) | 1.0 (0.5, 1.8) |
| American Samoa | 2.6 (1.8, 3.9) | 2.1 (1.5, 3.0) | 1.8 (1.0, 3.1) | 1.1 (0.6, 2.1) |
| Andorra | 0.59 (0.37, 0.87) | 0.60 (0.38, 0.85) | 0.36 (0.16, 0.66) | 0.29 (0.13, 0.57) |
| Angola | 19.7 (16.2, 24.6) | 17.0 (14.0, 21.1) | 36.5 (18.7, 59.3) | 19.1 (10.7, 29.8) |
| Antigua and Barbuda | 1.9 (1.4, 2.5) | 2.1 (1.6, 2.7) | 1.5 (0.8, 2.5) | 1.4 (0.7, 2.3) |
| Argentina | 1.1 (0.8, 1.4) | 0.82 (0.55, 1.12) | 0.92 (0.50, 1.52) | 0.46 (0.23, 0.80) |
| Armenia | 1.4 (1.0, 1.8) | 1.1 (0.8, 1.5) | 1.3 (0.7, 2.1) | 0.81 (0.42, 1.40) |
| Australia | 0.62 (0.39, 0.89) | 0.72 (0.46, 1.03) | 0.35 (0.17, 0.61) | 0.30 (0.14, 0.56) |
| Austria | 0.59 (0.37, 0.87) | 0.61 (0.38, 0.86) | 0.44 (0.21, 0.79) | 0.30 (0.13, 0.57) |
| Azerbaijan | 1.5 (1.1, 2.0) | 1.3 (1.0, 1.7) | 1.4 (0.7, 2.2) | 1.00 (0.51, 1.69) |
| Bahrain | 1.2 (0.8, 1.6) | 1.4 (1.0, 1.9) | 1.00 (0.52, 1.74) | 0.76 (0.39, 1.31) |
| Bangladesh | 3.7 (2.4, 5.5) | 4.2 (3.0, 5.9) | 6.0 (3.1, 10.7) | 4.9 (2.6, 8.4) |
| Barbados | 1.5 (1.1, 2.0) | 1.7 (1.3, 2.2) | 1.0 (0.6, 1.8) | 0.85 (0.43, 1.50) |
| Belarus | 1.4 (1.1, 1.8) | 1.2 (0.9, 1.6) | 1.2 (0.6, 1.9) | 0.65 (0.33, 1.16) |
| Belgium | 0.58 (0.36, 0.82) | 0.63 (0.41, 0.88) | 0.41 (0.19, 0.72) | 0.29 (0.13, 0.55) |
| Belize | 3.6 (2.8, 4.5) | 4.6 (3.5, 5.8) | 3.7 (2.1, 5.9) | 3.3 (1.8, 5.4) |
| Benin | 39.3 (28.9, 57.3) | 52.9 (39.7, 74.1) | 84.0 (44.3, 142.4) | 79.2 (42.6, 135.4) |

| Country | Incidence (per 100,000) | | Mortality (per million) | |
|---|---|---|---|---|
| | 1990 | 2017 | 1990 | 2017 |
| Bermuda | 1.5 (1.1, 2.0) | 1.9 (1.4, 2.4) | 0.96 (0.50, 1.65) | 0.91 (0.46, 1.63) |
| Bhutan | 1.7 (1.1, 2.5) | 1.3 (0.8, 1.9) | 2.3 (1.1, 4.0) | 1.1 (0.5, 2.0) |
| Bolivia | 2.1 (1.5, 2.7) | 1.6 (1.2, 2.1) | 2.6 (1.4, 4.2) | 1.5 (0.7, 2.5) |
| Bosnia and Herzegovina | 0.97 (0.66, 1.28) | 0.77 (0.54, 1.05) | 1.1 (0.6, 1.8) | 0.60 (0.30, 1.03) |
| Botswana | 16.1 (13.2, 20.2) | 8.5 (6.9, 10.5) | 11.6 (6.0, 19.2) | 2.7 (1.4, 4.8) |
| Brazil | 1.9 (1.4, 2.4) | 2.0 (1.5, 2.6) | 2.1 (1.2, 3.4) | 1.3 (0.7, 2.2) |
| Brunei | 0.78 (0.52, 1.10) | 0.71 (0.46, 1.00) | 0.46 (0.23, 0.81) | 0.34 (0.15, 0.63) |
| Bulgaria | 0.80 (0.57, 1.10) | 0.75 (0.52, 1.04) | 0.72 (0.38, 1.24) | 0.47 (0.23, 0.83) |
| Burkina Faso | 93.8 (77.5, 114.5) | 94.7 (79.6, 112.4) | 172.6 (92.2, 281.6) | 168.0 (92.9, 263.6) |
| Burundi | 23.2 (13.8, 45.9) | 10.2 (5.5, 23.1) | 34.6 (16.1, 72.3) | 13.2 (6.0, 30.4) |
| Cambodia | 2.1 (1.6, 2.7) | 1.7 (1.3, 2.3) | 3.0 (1.6, 4.8) | 1.5 (0.8, 2.6) |
| Cameroon | 46.3 (33.8, 66.9) | 46.3 (34.1, 66.5) | 77.5 (40.7, 132.8) | 45.0 (22.8, 78.6) |
| Canada | 0.28 (0.17, 0.45) | 0.29 (0.17, 0.46) | 0.15 (0.07, 0.28) | 0.12 (0.05, 0.22) |
| Cape Verde | 28.9 (21.2, 43.5) | 9.0 (6.3, 13.6) | 43.5 (23.1, 75.5) | 10.1 (5.5, 18.2) |
| Central African Republic | 29.5 (24.4, 37.0) | 30.9 (25.5, 39.9) | 40.4 (20.1, 68.5) | 39.7 (22.2, 66.4) |
| Chad | 50.0 (36.9, 71.2) | 37.6 (27.7, 55.0) | 88.2 (44.8, 148.3) | 64.8 (34.4, 108.9) |
| Chile | 0.63 (0.39, 0.91) | 0.77 (0.51, 1.08) | 0.55 (0.27, 0.95) | 0.40 (0.19, 0.74) |
| China | 0.74 (0.51, 1.03) | 0.73 (0.50, 1.02) | 0.92 (0.49, 1.57) | 0.44 (0.22, 0.75) |
| Colombia | 1.8 (1.3, 2.3) | 1.7 (1.2, 2.2) | 1.8 (1.0, 3.0) | 1.2 (0.6, 2.0) |
| Comoros | 7.2 (3.5, 17.1) | 5.1 (2.1, 13.1) | 14.4 (6.3, 33.7) | 7.5 (2.7, 18.4) |

| Country | Incidence (per 100,000) | | Mortality (per million) | |
|---|---|---|---|---|
| | 1990 | 2017 | 1990 | 2017 |
| Congo | 22.2 (18.3, 27.9) | 17.0 (14.0, 21.3) | 25.0 (13.8, 41.3) | 14.7 (8.0, 24.3) |
| Costa Rica | 1.6 (1.1, 2.0) | 1.4 (1.0, 1.9) | 1.5 (0.8, 2.5) | 0.86 (0.45, 1.48) |
| Cote d'Ivoire | 67.4 (50.2, 89.5) | 59.7 (44.6, 80.4) | 90.0 (49.5, 145.7) | 77.0 (43.3, 129.2) |
| Croatia | 0.77 (0.53, 1.07) | 0.75 (0.51, 1.04) | 0.60 (0.31, 1.05) | 0.40 (0.19, 0.72) |
| Cuba | 2.0 (1.5, 2.6) | 2.0 (1.5, 2.6) | 1.6 (0.9, 2.7) | 1.3 (0.7, 2.2) |
| Cyprus | 0.62 (0.41, 0.88) | 0.59 (0.38, 0.86) | 0.60 (0.29, 1.02) | 0.34 (0.15, 0.64) |
| Czech Republic | 0.75 (0.50, 1.04) | 0.74 (0.49, 1.02) | 0.62 (0.32, 1.05) | 0.37 (0.18, 0.67) |
| Democratic Republic of the Congo | 71.1 (58.5, 87.7) | 35.9 (29.8, 44.2) | 93.3 (50.9, 152.7) | 49.4 (27.8, 78.4) |
| Denmark | 1.8 (1.4, 2.2) | 1.5 (1.2, 2.0) | 1.4 (0.7, 2.4) | 0.96 (0.44, 1.72) |
| Djibouti | 9.1 (5.0, 19.1) | 5.8 (2.9, 12.1) | 13.2 (5.6, 29.3) | 6.3 (2.6, 14.5) |
| Dominica | 2.5 (1.9, 3.2) | 2.5 (1.8, 3.2) | 2.2 (1.2, 3.6) | 2.0 (1.0, 3.4) |
| Dominican Republic | 3.2 (2.5, 4.0) | 4.2 (3.3, 5.3) | 4.0 (2.3, 6.4) | 4.0 (2.2, 6.7) |
| Ecuador | 1.7 (1.2, 2.2) | 1.5 (1.1, 2.0) | 1.6 (0.9, 2.7) | 1.2 (0.6, 2.0) |
| Egypt | 1.7 (1.3, 2.2) | 1.4 (1.1, 1.9) | 2.2 (1.2, 3.5) | 1.3 (0.7, 2.2) |
| El Salvador | 2.8 (2.1, 3.5) | 2.2 (1.7, 2.9) | 3.5 (2.0, 5.7) | 1.8 (1.0, 3.0) |
| Equatorial Guinea | 46.7 (38.6, 58.4) | 25.7 (21.1, 31.9) | 82.0 (43.1, 134.2) | 14.2 (7.2, 25.3) |
| Eritrea | 10.1 (5.7, 20.8) | 6.9 (3.4, 17.1) | 22.9 (9.4, 49.5) | 14.2 (6.1, 34.9) |
| Estonia | 1.2 (0.9, 1.6) | 1.2 (0.9, 1.6) | 0.86 (0.46, 1.49) | 0.56 (0.27, 1.01) |
| Ethiopia | 0.53 (0.32, 0.80) | 0.46 (0.27, 0.70) | 0.88 (0.39, 1.62) | 0.76 (0.37, 1.31) |
| Federated States of Micronesia | 4.4 (3.1, 6.8) | 3.0 (2.2, 4.1) | 4.9 (2.6, 8.6) | 2.7 (1.3, 4.6) |

| Country | Incidence (per 100,000) | | Mortality (per million) | |
|---|---|---|---|---|
| | 1990 | 2017 | 1990 | 2017 |
| Fiji | 2.7 (1.9, 4.1) | 2.2 (1.6, 3.2) | 2.4 (1.3, 4.4) | 1.8 (0.9, 3.2) |
| Finland | 0.27 (0.16, 0.43) | 0.28 (0.17, 0.46) | 0.15 (0.07, 0.28) | 0.12 (0.05, 0.23) |
| France | 0.59 (0.37, 0.87) | 0.56 (0.35, 0.82) | 0.43 (0.21, 0.78) | 0.30 (0.13, 0.57) |
| Gabon | 24.6 (20.2, 30.8) | 6.6 (5.3, 8.4) | 27.5 (15.2, 44.3) | 4.2 (2.3, 7.1) |
| Georgia | 1.6 (1.1, 2.0) | 1.5 (1.1, 1.9) | 1.2 (0.6, 1.9) | 1.1 (0.6, 1.8) |
| Germany | 0.60 (0.39, 0.85) | 0.58 (0.38, 0.82) | 0.45 (0.21, 0.80) | 0.31 (0.13, 0.57) |
| Ghana | 27.6 (22.5, 36.2) | 27.3 (22.2, 33.9) | 32.0 (18.1, 51.5) | 28.0 (16.2, 44.5) |
| Greece | 0.61 (0.41, 0.86) | 0.58 (0.35, 0.84) | 0.53 (0.26, 0.92) | 0.38 (0.18, 0.68) |
| Greenland | 0.53 (0.31, 0.80) | 0.46 (0.27, 0.74) | 0.44 (0.21, 0.79) | 0.29 (0.13, 0.56) |
| Grenada | 2.8 (2.1, 3.5) | 2.2 (1.7, 2.8) | 3.2 (1.8, 5.1) | 1.5 (0.8, 2.5) |
| Guam | 1.6 (1.1, 2.4) | 1.9 (1.3, 2.9) | 0.96 (0.51, 1.76) | 0.87 (0.42, 1.66) |
| Guatemala | 2.5 (1.9, 3.2) | 2.2 (1.7, 2.8) | 4.0 (2.3, 6.3) | 2.1 (1.1, 3.4) |
| Guinea | 45.6 (33.6, 65.1) | 72.6 (54.0, 98.5) | 107.9 (54.5, 184.5) | 116.5 (61.8, 194.2) |
| Guinea-Bissau | 34.6 (28.0, 45.7) | 18.1 (14.5, 23.1) | 73.7 (38.9, 122.8) | 26.7 (14.4, 45.8) |
| Guyana | 2.8 (2.1, 3.6) | 3.6 (2.8, 4.6) | 2.9 (1.6, 4.7) | 2.6 (1.5, 4.4) |
| Haiti | 4.8 (3.8, 5.9) | 6.3 (5.0, 7.7) | 6.4 (3.7, 10.3) | 6.6 (3.8, 10.4) |
| Honduras | 2.7 (2.0, 3.4) | 2.5 (1.9, 3.2) | 4.7 (2.7, 7.5) | 3.1 (1.7, 5.3) |
| Hungary | 0.80 (0.55, 1.09) | 0.72 (0.48, 1.00) | 0.68 (0.35, 1.14) | 0.39 (0.19, 0.68) |
| Iceland | 0.63 (0.42, 0.87) | 0.60 (0.37, 0.85) | 0.42 (0.20, 0.75) | 0.30 (0.13, 0.56) |
| India | 1.8 (1.4, 2.4) | 2.2 (1.7, 2.8) | 2.4 (1.3, 3.9) | 1.8 (1.0, 2.9) |

| Country | Incidence (per 100,000) | | Mortality (per million) | |
|---|---|---|---|---|
| | 1990 | 2017 | 1990 | 2017 |
| Indonesia | 1.5 (1.1, 2.0) | 1.7 (1.3, 2.2) | 1.5 (0.8, 2.4) | 1.2 (0.7, 2.1) |
| Iran | 1.6 (1.2, 2.1) | 1.6 (1.2, 2.1) | 1.7 (0.9, 2.9) | 1.1 (0.6, 2.0) |
| Iraq | 2.1 (1.5, 2.6) | 2.9 (2.3, 3.7) | 2.5 (1.3, 4.2) | 2.1 (1.2, 3.5) |
| Ireland | 0.65 (0.44, 0.90) | 0.60 (0.37, 0.85) | 0.51 (0.24, 0.88) | 0.31 (0.14, 0.60) |
| Israel | 3.1 (2.5, 3.8) | 2.8 (2.2, 3.4) | 3.3 (1.7, 5.4) | 2.1 (1.0, 3.7) |
| Italy | 0.64 (0.43, 0.92) | 0.62 (0.42, 0.89) | 0.48 (0.23, 0.87) | 0.34 (0.14, 0.62) |
| Jamaica | 2.2 (1.7, 2.9) | 2.4 (1.8, 3.1) | 1.9 (1.1, 3.2) | 1.7 (0.9, 2.9) |
| Japan | 1.0 (0.7, 1.4) | 0.88 (0.59, 1.20) | 0.52 (0.26, 0.94) | 0.38 (0.18, 0.69) |
| Jordan | 1.6 (1.2, 2.1) | 1.3 (0.9, 1.8) | 1.9 (1.0, 3.1) | 1.0 (0.6, 1.8) |
| Kazakhstan | 1.4 (1.0, 1.8) | 1.3 (1.0, 1.7) | 1.2 (0.7, 2.1) | 0.87 (0.45, 1.51) |
| Kenya | 93.4 (77.5, 112.6) | 53.7 (43.9, 66.7) | 126.0 (71.4, 197.0) | 52.2 (30.4, 81.5) |
| Kiribati | 6.1 (4.4, 9.4) | 4.9 (3.5, 7.0) | 9.0 (5.0, 15.5) | 7.2 (3.9, 11.9) |
| Kuwait | 1.0 (0.7, 1.4) | 1.2 (0.9, 1.6) | 0.71 (0.36, 1.20) | 0.56 (0.28, 1.03) |
| Kyrgyzstan | 1.9 (1.4, 2.5) | 1.5 (1.1, 2.0) | 2.0 (1.1, 3.2) | 1.4 (0.8, 2.3) |
| Laos | 2.0 (1.5, 2.6) | 1.8 (1.4, 2.3) | 2.8 (1.5, 4.5) | 1.9 (1.0, 3.0) |
| Latvia | 1.3 (1.0, 1.7) | 1.3 (0.9, 1.7) | 0.92 (0.49, 1.54) | 0.65 (0.32, 1.15) |
| Lebanon | 1.4 (1.0, 1.9) | 1.9 (1.4, 2.4) | 1.4 (0.7, 2.4) | 1.2 (0.6, 2.2) |
| Lesotho | 19.9 (16.3, 24.6) | 14.0 (11.4, 17.8) | 21.6 (11.8, 34.6) | 6.0 (3.1, 10.4) |
| Liberia | 35.1 (25.9, 51.3) | 46.8 (34.6, 67.5) | 74.7 (38.8, 131.1) | 66.4 (34.4, 115.9) |
| Libya | 1.2 (0.8, 1.6) | 1.3 (0.9, 1.7) | 1.1 (0.6, 1.9) | 1.1 (0.6, 2.0) |

| Country | Incidence (per 100,000) | | Mortality (per million) | |
|---|---|---|---|---|
| | 1990 | 2017 | 1990 | 2017 |
| Lithuania | 1.3 (1.0, 1.7) | 1.2 (0.9, 1.6) | 0.91 (0.48, 1.54) | 0.57 (0.28, 1.00) |
| Luxembourg | 0.60 (0.38, 0.85) | 0.60 (0.41, 0.86) | 0.37 (0.17, 0.68) | 0.32 (0.14, 0.63) |
| Macedonia | 0.74 (0.50, 1.02) | 0.73 (0.50, 1.01) | 0.73 (0.38, 1.26) | 0.56 (0.28, 0.98) |
| Madagascar | 0.33 (0.19, 0.49) | 0.31 (0.19, 0.48) | 0.64 (0.30, 1.16) | 0.49 (0.22, 0.87) |
| Malawi | 155.4 (129.0, 186.1) | 30.7 (26.2, 35.6) | 193.8 (79.6, 327.9) | 33.1 (17.7, 56.0) |
| Malaysia | 0.97 (0.66, 1.33) | 1.0 (0.7, 1.4) | 0.78 (0.40, 1.31) | 0.46 (0.22, 0.84) |
| Maldives | 2.7 (2.0, 3.4) | 2.3 (1.7, 3.0) | 3.4 (1.9, 5.4) | 1.5 (0.8, 2.5) |
| Mali | 75.3 (56.2, 101.0) | 94.9 (73.0, 123.4) | 167.1 (88.2, 281.1) | 202.4 (113.7, 322.5) |
| Malta | 0.59 (0.37, 0.87) | 0.63 (0.41, 0.88) | 0.53 (0.25, 0.93) | 0.39 (0.18, 0.71) |
| Marshall Islands | 4.1 (2.9, 6.5) | 3.3 (2.4, 4.7) | 4.8 (2.7, 8.1) | 3.2 (1.7, 5.4) |
| Mauritania | 26.4 (19.3, 38.6) | 17.6 (12.5, 27.6) | 44.8 (24.4, 74.3) | 25.5 (14.0, 43.4) |
| Mauritius | 0.98 (0.69, 1.33) | 1.2 (0.8, 1.5) | 0.94 (0.52, 1.60) | 0.69 (0.36, 1.20) |
| Mexico | 2.0 (1.5, 2.6) | 1.8 (1.3, 2.3) | 2.0 (1.1, 3.3) | 1.2 (0.7, 2.0) |
| Moldova | 2.1 (1.6, 2.8) | 2.0 (1.5, 2.6) | 2.2 (1.2, 3.5) | 1.4 (0.8, 2.4) |
| Mongolia | 1.6 (1.2, 2.1) | 2.1 (1.6, 2.6) | 1.8 (1.0, 2.9) | 1.7 (0.9, 2.9) |
| Montenegro | 0.85 (0.59, 1.16) | 0.77 (0.53, 1.06) | 0.76 (0.39, 1.26) | 0.50 (0.25, 0.90) |
| Morocco | 3.0 (2.3, 3.8) | 2.1 (1.6, 2.8) | 4.0 (2.2, 6.4) | 2.2 (1.2, 3.7) |
| Mozambique | 71.4 (59.0, 88.4) | 24.3 (20.4, 29.2) | 164.0 (88.5, 263.9) | 28.2 (15.2, 45.9) |
| Myanmar | 2.5 (1.9, 3.1) | 2.2 (1.7, 2.8) | 3.8 (2.0, 6.1) | 2.2 (1.2, 3.6) |
| Namibia | 25.7 (21.1, 32.0) | 11.0 (9.0, 13.7) | 25.1 (13.8, 40.4) | 4.9 (2.5, 8.5) |

| Country | Incidence (per 100,000) | | Mortality (per million) | |
|---|---|---|---|---|
| | 1990 | 2017 | 1990 | 2017 |
| Nepal | 3.9 (2.4, 5.9) | 3.1 (2.1, 4.2) | 5.3 (2.5, 9.5) | 3.1 (1.6, 5.2) |
| Netherlands | 0.61 (0.42, 0.86) | 0.60 (0.38, 0.86) | 0.41 (0.19, 0.73) | 0.29 (0.13, 0.55) |
| New Zealand | 0.83 (0.56, 1.15) | 0.99 (0.66, 1.35) | 0.52 (0.26, 0.91) | 0.43 (0.21, 0.80) |
| Nicaragua | 2.6 (2.0, 3.3) | 2.8 (2.2, 3.6) | 4.0 (2.2, 6.4) | 2.6 (1.5, 4.3) |
| Niger | 54.7 (40.0, 74.7) | 64.3 (47.7, 89.7) | 119.4 (60.9, 205.1) | 123.6 (64.3, 208.1) |
| Nigeria | 67.7 (50.8, 89.7) | 63.8 (47.7, 85.8) | 85.2 (47.1, 136.9) | 65.0 (34.7, 109.2) |
| North Korea | 2.9 (1.3, 5.4) | 3.1 (1.7, 5.0) | 3.5 (1.3, 7.4) | 3.2 (1.5, 6.0) |
| Northern Mariana Islands | 1.9 (1.3, 2.8) | 2.1 (1.4, 2.9) | 0.87 (0.43, 1.58) | 1.0 (0.5, 1.9) |
| Norway | 0.89 (0.64, 1.19) | 0.88 (0.63, 1.19) | 0.56 (0.28, 0.99) | 0.36 (0.17, 0.68) |
| Oman | 4.3 (3.4, 5.4) | 1.7 (1.2, 2.2) | 4.9 (2.7, 8.0) | 0.97 (0.49, 1.71) |
| Pakistan | 4.7 (3.3, 6.5) | 4.2 (2.8, 6.0) | 6.7 (3.5, 11.4) | 5.8 (3.0, 10.3) |
| Palestine | 2.0 (1.5, 2.6) | 3.4 (2.6, 4.2) | 3.6 (1.9, 6.1) | 3.7 (2.1, 6.2) |
| Panama | 1.6 (1.2, 2.0) | 1.7 (1.3, 2.3) | 1.4 (0.7, 2.3) | 1.1 (0.6, 2.0) |
| Papua New Guinea | 6.6 (4.9, 10.1) | 6.3 (4.6, 9.1) | 9.9 (5.4, 16.9) | 7.8 (4.4, 13.3) |
| Paraguay | 2.4 (1.8, 3.1) | 1.8 (1.3, 2.3) | 2.6 (1.4, 4.3) | 1.1 (0.6, 1.9) |
| Peru | 1.4 (1.0, 1.8) | 1.5 (1.1, 2.0) | 1.6 (0.9, 2.7) | 1.1 (0.6, 1.9) |
| Philippines | 2.1 (1.5, 2.6) | 1.8 (1.4, 2.4) | 2.1 (1.2, 3.5) | 1.6 (0.9, 2.7) |
| Poland | 0.79 (0.55, 1.08) | 0.72 (0.49, 1.01) | 0.71 (0.37, 1.18) | 0.38 (0.19, 0.71) |
| Portugal | 0.65 (0.44, 0.88) | 0.63 (0.40, 0.90) | 0.68 (0.34, 1.16) | 0.32 (0.15, 0.59) |
| Puerto Rico | 1.4 (1.0, 1.8) | 1.6 (1.2, 2.1) | 0.86 (0.45, 1.52) | 0.80 (0.40, 1.46) |

| Country | Incidence (per 100,000) | | Mortality (per million) | |
|---|---|---|---|---|
| | 1990 | 2017 | 1990 | 2017 |
| Qatar | 1.1 (0.7, 1.4) | 1.3 (0.9, 1.7) | 1.1 (0.6, 2.0) | 0.98 (0.47, 1.80) |
| Romania | 1.2 (0.9, 1.5) | 0.77 (0.52, 1.05) | 1.1 (0.6, 1.8) | 0.49 (0.25, 0.86) |
| Russian Federation | 1.6 (1.2, 2.1) | 1.6 (1.2, 2.1) | 1.2 (0.7, 2.1) | 0.76 (0.39, 1.34) |
| Rwanda | 11.7 (6.6, 25.1) | 5.5 (2.6, 12.1) | 18.5 (8.4, 39.8) | 7.1 (2.9, 17.6) |
| Saint Lucia | 2.5 (1.9, 3.2) | 2.2 (1.7, 2.8) | 2.4 (1.3, 4.0) | 1.6 (0.9, 2.6) |
| Saint Vincent and the Grenadines | 2.8 (2.1, 3.5) | 2.6 (2.0, 3.3) | 2.8 (1.6, 4.5) | 2.3 (1.2, 3.7) |
| Samoa | 3.6 (2.5, 5.9) | 2.8 (2.0, 4.3) | 3.2 (1.7, 5.9) | 2.2 (1.1, 3.8) |
| Sao Tome and Principe | 25.2 (18.2, 38.9) | 15.5 (11.2, 23.5) | 52.9 (27.9, 92.0) | 24.9 (13.6, 44.3) |
| Saudi Arabia | 2.5 (1.9, 3.1) | 1.7 (1.2, 2.2) | 2.7 (1.5, 4.4) | 0.79 (0.39, 1.41) |
| Senegal | 31.4 (25.0, 42.7) | 10.6 (8.5, 13.7) | 65.7 (36.6, 105.1) | 18.7 (10.8, 29.9) |
| Serbia | 0.99 (0.71, 1.31) | 0.80 (0.56, 1.09) | 1.1 (0.6, 1.8) | 0.53 (0.27, 0.92) |
| Seychelles | 1.0 (0.7, 1.4) | 1.1 (0.8, 1.5) | 0.91 (0.46, 1.51) | 0.73 (0.38, 1.22) |
| Sierra Leone | 59.2 (43.5, 81.8) | 68.9 (51.9, 93.4) | 127.0 (65.6, 217.6) | 94.6 (51.2, 154.4) |
| Singapore | 0.79 (0.52, 1.10) | 0.67 (0.42, 0.96) | 0.51 (0.25, 0.88) | 0.26 (0.12, 0.49) |
| Slovakia | 0.76 (0.52, 1.04) | 0.74 (0.51, 1.01) | 0.65 (0.34, 1.09) | 0.40 (0.19, 0.72) |
| Slovenia | 0.76 (0.52, 1.05) | 0.72 (0.51, 0.99) | 0.57 (0.30, 0.98) | 0.36 (0.17, 0.66) |
| Solomon Islands | 5.1 (3.6, 8.2) | 4.5 (3.2, 6.5) | 6.6 (3.5, 11.0) | 5.0 (2.8, 8.4) |
| Somalia | 11.5 (6.3, 25.8) | 7.4 (3.8, 16.6) | 21.1 (6.6, 52.4) | 18.6 (7.3, 45.6) |
| South Africa | 14.9 (9.9, 23.7) | 6.1 (4.5, 9.8) | 12.0 (6.5, 21.4) | 2.7 (1.4, 5.8) |
| South Korea | 0.82 (0.54, 1.13) | 0.67 (0.42, 0.96) | 0.59 (0.29, 1.01) | 0.29 (0.13, 0.55) |

| Country | Incidence (per 100,000) | | Mortality (per million) | |
|---|---|---|---|---|
| | 1990 | 2017 | 1990 | 2017 |
| South Sudan | 14.9<br>(8.3, 31.1) | 10.4<br>(5.5, 24.6) | 23.1<br>(8.1, 56.7) | 18.6<br>(8.4, 48.3) |
| Spain | 0.60<br>(0.38, 0.87) | 0.60<br>(0.39, 0.86) | 0.48<br>(0.23, 0.84) | 0.30<br>(0.13, 0.59) |
| Sri Lanka | 2.0<br>(1.4, 2.5) | 1.4<br>(1.0, 1.8) | 1.7<br>(0.9, 2.7) | 0.80<br>(0.43, 1.37) |
| Sudan | 3.0<br>(2.4, 3.9) | 4.7<br>(3.7, 5.8) | 4.6<br>(2.3, 7.7) | 5.2<br>(3.0, 8.5) |
| Suriname | 2.6<br>(2.0, 3.4) | 2.7<br>(2.1, 3.4) | 2.3<br>(1.3, 3.8) | 1.7<br>(0.9, 2.8) |
| Swaziland | 18.9<br>(15.5, 23.5) | 11.2<br>(9.1, 14.0) | 24.7<br>(13.9, 39.6) | 4.2<br>(2.2, 7.4) |
| Sweden | 0.90<br>(0.63, 1.20) | 0.89<br>(0.62, 1.20) | 0.61<br>(0.31, 1.07) | 0.46<br>(0.22, 0.84) |
| Switzerland | 0.62<br>(0.41, 0.85) | 0.62<br>(0.42, 0.85) | 0.35<br>(0.16, 0.63) | 0.29<br>(0.12, 0.56) |
| Syria | 1.9<br>(1.4, 2.5) | 1.6<br>(1.2, 2.1) | 2.5<br>(1.3, 4.0) | 1.5<br>(0.8, 2.5) |
| Taiwan | 5.3<br>(4.1, 6.7) | 4.4<br>(3.3, 5.5) | 3.9<br>(2.2, 6.3) | 1.9<br>(1.0, 3.4) |
| Tajikistan | 1.7<br>(1.2, 2.2) | 1.5<br>(1.1, 1.9) | 2.1<br>(1.1, 3.4) | 1.9<br>(1.0, 3.0) |
| Tanzania | 17.0<br>(13.8, 21.9) | 5.3<br>(4.3, 6.7) | 21.6<br>(10.7, 36.4) | 7.1<br>(4.0, 11.7) |
| Thailand | 2.4<br>(1.8, 3.0) | 2.3<br>(1.7, 2.9) | 2.3<br>(1.3, 3.8) | 1.4<br>(0.7, 2.4) |
| The Bahamas | 1.6<br>(1.2, 2.0) | 1.9<br>(1.4, 2.5) | 1.1<br>(0.5, 1.8) | 0.91<br>(0.46, 1.62) |
| The Gambia | 65.9<br>(53.9, 82.7) | 29.3<br>(23.5, 38.2) | 130.3<br>(72.7, 213.3) | 45.4<br>(24.8, 74.9) |
| Timor-Leste | 1.4<br>(1.0, 1.8) | 1.3<br>(1.0, 1.8) | 1.9<br>(1.0, 3.3) | 1.6<br>(0.8, 2.6) |
| Togo | 61.3<br>(45.8, 83.1) | 90.3<br>(68.6, 117.1) | 113.8<br>(62.6, 191.2) | 96.6<br>(54.2, 161.0) |
| Tonga | 4.3<br>(3.1, 6.6) | 3.4<br>(2.4, 4.9) | 3.0<br>(1.6, 5.2) | 2.3<br>(1.2, 4.0) |
| Trinidad and Tobago | 2.0<br>(1.5, 2.6) | 2.1<br>(1.5, 2.7) | 1.6<br>(0.9, 2.7) | 1.2<br>(0.6, 2.1) |
| Tunisia | 1.8<br>(1.4, 2.4) | 1.9<br>(1.4, 2.4) | 2.0<br>(1.1, 3.3) | 1.1<br>(0.6, 2.0) |

| Country | Incidence (per 100,000) | | Mortality (per million) | |
|---|---|---|---|---|
| | 1990 | 2017 | 1990 | 2017 |
| Turkey | 1.9 (1.5, 2.6) | 1.9 (1.4, 2.4) | 2.2 (1.2, 3.5) | 1.1 (0.6, 1.8) |
| Turkmenistan | 2.3 (1.7, 2.9) | 1.5 (1.1, 1.9) | 2.3 (1.3, 3.8) | 1.2 (0.7, 2.1) |
| Uganda | 27.2 (16.2, 51.8) | 6.3 (3.1, 14.6) | 27.1 (12.2, 58.2) | 6.4 (2.6, 16.9) |
| Ukraine | 1.6 (1.2, 2.1) | 1.7 (1.3, 2.2) | 1.2 (0.7, 2.1) | 2.1 (1.1, 3.6) |
| United Arab Emirates | 1.5 (1.1, 2.0) | 1.4 (1.0, 1.9) | 1.4 (0.7, 2.4) | 0.93 (0.46, 1.69) |
| United Kingdom | 0.86 (0.62, 1.14) | 0.85 (0.61, 1.13) | 0.67 (0.35, 1.13) | 0.42 (0.20, 0.74) |
| United States | 1.9 (1.4, 2.4) | 2.0 (1.5, 2.5) | 1.2 (0.6, 2.0) | 0.84 (0.40, 1.51) |
| Uruguay | 0.64 (0.41, 0.91) | 0.76 (0.46, 1.10) | 0.56 (0.28, 0.95) | 0.40 (0.19, 0.72) |
| Uzbekistan | 1.2 (0.9, 1.6) | 1.1 (0.8, 1.4) | 1.4 (0.8, 2.3) | 0.98 (0.52, 1.72) |
| Vanuatu | 5.8 (4.2, 8.8) | 5.3 (3.8, 7.7) | 9.6 (5.0, 16.2) | 8.7 (4.6, 14.5) |
| Venezuela | 1.5 (1.1, 1.9) | 1.5 (1.1, 1.9) | 1.4 (0.8, 2.4) | 0.99 (0.53, 1.69) |
| Vietnam | 1.2 (0.8, 1.6) | 1.2 (0.9, 1.5) | 1.4 (0.7, 2.4) | 0.85 (0.45, 1.43) |
| Virgin Islands, U.S. | 1.4 (1.0, 1.8) | 1.7 (1.3, 2.2) | 0.87 (0.47, 1.46) | 1.0 (0.5, 1.8) |
| Yemen | 4.0 (3.2, 4.9) | 5.5 (4.3, 6.8) | 6.4 (2.7, 11.1) | 7.0 (3.9, 11.5) |
| Zambia | 11.8 (6.4, 22.9) | 7.6 (3.8, 17.3) | 15.4 (6.4, 34.9) | 6.9 (2.7, 20.0) |
| Zimbabwe | 16.9 (13.9, 21.1) | 16.0 (13.0, 20.0) | 11.3 (6.0, 18.2) | 9.4 (5.0, 15.1) |

**Table S8: Estimated percentage of iNTS cases that were attributable to HIV, by country, with 95% uncertainty intervals, in 1990, 2005, and 2017.**

| Country | 1990 | 2005 | 2017 |
|---|---|---|---|
| Afghanistan | 0.0<br>(0.0 – 0.1) | 0.0<br>(0.0 – 0.1) | 0.1<br>(0.0 – 0.3) |
| Albania | 0.0<br>(0.0 – <0.1) | 0.0<br>(0.0 – <0.1) | 0.0<br>(0.0 – 0.1) |
| Algeria | 0.0<br>(0.0 – 0.1) | 0.2<br>(0.1 – 0.3) | 0.6<br>(0.3 – 1.1) |
| American Samoa | 0.0<br>(0.0 – 0.1) | 0.0<br>(0.0 – 0.1) | 0.1<br>(0.0 – 0.2) |
| Andorra | 6.5<br>(1.7 – 14.8) | 10.4<br>(2.8 – 23.5) | 13.2<br>(4.3 – 28.0) |
| Angola | 0.3<br>(0.1 – 0.8) | 6.6<br>(3.0 – 12.0) | 8.5<br>(4.1 – 14.7) |
| Antigua and Barbuda | 1.4<br>(0.7 – 2.5) | 1.9<br>(1.0 – 3.3) | 1.8<br>(1.0 – 3.0) |
| Argentina | 1.5<br>(0.7 – 2.6) | 6.4<br>(3.2 – 10.6) | 12.2<br>(6.5 – 19.9) |
| Armenia | 0.0<br>(0.0 – <0.1) | 0.1<br>(0.1 – 0.2) | 1.4<br>(0.7 – 2.4) |
| Australia | 3.0<br>(1.2 – 5.9) | 4.3<br>(1.6 – 9.3) | 4.8<br>(1.9 – 9.8) |
| Austria | 2.1<br>(0.7 – 4.8) | 9.1<br>(3.2 – 19.4) | 16.0<br>(6.2 – 31.7) |
| Azerbaijan | 0.0<br>(0.0 – 0.1) | 0.1<br>(0.1 – 0.2) | 0.6<br>(0.3 – 1.0) |
| Bahrain | 0.2<br>(0.1 – 0.4) | 0.4<br>(0.2 – 0.6) | 0.5<br>(0.3 – 0.8) |
| Bangladesh | 0.0<br>(0.0 – <0.1) | 0.0<br>(0.0 – <0.1) | 0.1<br>(0.0 – 0.1) |
| Barbados | 2.6<br>(1.4 – 4.4) | 6.4<br>(3.7 – 10.1) | 9.3<br>(5.6 – 14.3) |
| Belarus | 0.1<br>(0.1 – 0.3) | 1.2<br>(0.6 – 2.1) | 4.5<br>(2.3 – 7.4) |
| Belgium | 2.9<br>(0.8 – 7.3) | 15.4<br>(5.1 – 32.8) | 20.9<br>(7.7 – 40.6) |
| Belize | 1.3<br>(0.6 – 2.5) | 4.9<br>(2.6 – 8.3) | 6.7<br>(3.8 – 11.0) |
| Benin | 0.2<br>(0.1 – 0.4) | 4.5<br>(1.8 – 8.8) | 4.2<br>(1.7 – 8.2) |
| Bermuda | 3.7<br>(2.1 – 6.2) | 3.2<br>(1.8 – 5.3) | 2.3<br>(1.3 – 3.7) |
| Bhutan | 0.2<br>(0.0 – 0.4) | 0.7<br>(0.2 – 1.5) | 1.5<br>(0.6 – 3.3) |
| Bolivia | 0.7<br>(0.3 – 1.4) | 1.3<br>(0.6 – 2.4) | 3.1<br>(1.5 – 5.5) |
| Bosnia and Herzegovina | 0.0<br>(0.0 – <0.1) | 0.0<br>(0.0 – 0.1) | 0.0<br>(0.0 – 0.1) |

| Country | 1990 | 2005 | 2017 |
|---|---|---|---|
| Botswana | 23.3<br>(14.5 – 33.3) | 43.8<br>(33.9 – 54.8) | 49.2<br>(39.4 – 59.1) |
| Brazil | 1.3<br>(0.7 – 2.1) | 3.5<br>(1.9 – 5.7) | 7.0<br>(4.2 – 11.1) |
| Brunei | 0.4<br>(0.2 – 0.8) | 2.7<br>(0.9 – 6.0) | 5.7<br>(2.2 – 11.8) |
| Bulgaria | 0.2<br>(0.1 – 0.3) | 0.6<br>(0.3 – 1.2) | 1.0<br>(0.5 – 1.8) |
| Burkina Faso | 9.0<br>(4.5 – 15.2) | 7.1<br>(3.2 – 12.6) | 3.3<br>(1.4 – 6.2) |
| Burundi | 4.9<br>(1.6 – 10.2) | 10.0<br>(3.9 – 19.1) | 4.8<br>(1.6 – 10.4) |
| Cambodia | 0.1<br>(0.0 – 0.1) | 4.4<br>(2.1 – 7.9) | 4.2<br>(2.2 – 7.4) |
| Cameroon | 2.8<br>(1.1 – 5.6) | 12.7<br>(6.5 – 21.4) | 10.7<br>(5.5 – 17.9) |
| Canada | 7.6<br>(2.9 – 15.1) | 16.9<br>(6.1 – 33.3) | 19.5<br>(7.5 – 38.3) |
| Cape Verde | 1.9<br>(0.7 – 3.9) | 6.3<br>(2.8 – 11.2) | 8.9<br>(4.7 – 15.0) |
| Central African Republic | 16.1<br>(8.6 – 25.1) | 21.8<br>(12.6 – 32.8) | 15.4<br>(8.0 – 24.6) |
| Chad | 3.6<br>(1.4 – 6.9) | 6.7<br>(2.8 – 12.5) | 5.0<br>(2.0 – 9.3) |
| Chile | 0.9<br>(0.4 – 1.5) | 4.5<br>(2.1 – 8.0) | 9.9<br>(4.9 – 17.0) |
| China | 0.1<br>(0.0 – 0.1) | 0.5<br>(0.2 – 0.8) | 1.0<br>(0.5 – 1.6) |
| Colombia | 0.3<br>(0.1 – 0.5) | 1.7<br>(0.9 – 2.8) | 4.0<br>(2.3 – 6.5) |
| Comoros | 0.0<br>(0.0 – <0.1) | 0.0<br>(0.0 – 0.1) | 0.1<br>(0.0 – 0.2) |
| Congo | 16.0<br>(8.5 – 25.5) | 15.9<br>(8.2 – 25.4) | 16.3<br>(8.6 – 26.0) |
| Costa Rica | 0.6<br>(0.3 – 1.0) | 2.3<br>(1.3 – 3.8) | 4.3<br>(2.3 – 7.3) |
| Cote d'Ivoire | 11.9<br>(5.9 – 20.1) | 14.0<br>(7.5 – 23.3) | 11.5<br>(5.9 – 19.4) |
| Croatia | 0.1<br>(0.0 – 0.2) | 0.3<br>(0.2 – 0.6) | 0.8<br>(0.4 – 1.4) |
| Cuba | 0.1<br>(0.1 – 0.3) | 0.9<br>(0.5 – 1.6) | 4.5<br>(2.6 – 7.3) |
| Cyprus | 0.2<br>(0.1 – 0.4) | 2.1<br>(0.5 – 5.7) | 5.3<br>(1.4 – 13.1) |
| Czech Republic | 0.0<br>(0.0 – 0.1) | 0.3<br>(0.1 – 0.5) | 0.7<br>(0.4 – 1.3) |
| Democratic Republic of the Congo | 7.4<br>(3.3 – 13.2) | 8.6<br>(3.9 – 15.2) | 4.7<br>(1.9 – 9.0) |

| Country | 1990 | 2005 | 2017 |
|---|---|---|---|
| Denmark | 5.1<br>(1.4 – 12.1) | 13.5<br>(3.9 – 30.1) | 18.2<br>(5.7 – 37.4) |
| Djibouti | 0.0<br>(0.0 – 0.1) | 8.1<br>(2.7 – 16.7) | 6.9<br>(2.1 – 14.1) |
| Dominica | 0.9<br>(0.4 – 1.7) | 1.5<br>(0.8 – 2.7) | 1.6<br>(0.8 – 2.7) |
| Dominican Republic | 0.6<br>(0.3 – 1.2) | 6.0<br>(3.1 – 10.1) | 7.0<br>(3.9 – 11.5) |
| Ecuador | 0.2<br>(0.1 – 0.3) | 1.5<br>(0.8 – 2.5) | 3.7<br>(2.0 – 5.9) |
| Egypt | 0.0<br>(0.0 – <0.1) | 0.0<br>(0.0 – <0.1) | 0.1<br>(0.0 – 0.1) |
| El Salvador | 0.3<br>(0.1 – 0.6) | 2.4<br>(1.2 – 4.2) | 4.4<br>(2.4 – 7.4) |
| Equatorial Guinea | 2.0<br>(0.7 – 4.1) | 13.9<br>(7.4 – 21.8) | 20.3<br>(12.8 – 29.8) |
| Eritrea | 1.1<br>(0.3 – 2.7) | 5.4<br>(1.8 – 11.7) | 3.1<br>(0.9 – 7.1) |
| Estonia | 0.0<br>(0.0 – <0.1) | 2.1<br>(1.1 – 3.5) | 6.0<br>(3.3 – 9.6) |
| Ethiopia | 5.8<br>(2.3 – 12.0) | 8.2<br>(3.6 – 15.3) | 5.2<br>(2.2 – 10.3) |
| Federated States of Micronesia | 0.1<br>(0.0 – 0.3) | 0.4<br>(0.1 – 1.0) | 2.3<br>(0.9 – 4.2) |
| Fiji | 0.1<br>(0.0 – 0.1) | 0.2<br>(0.1 – 0.4) | 0.5<br>(0.2 – 0.9) |
| Finland | 1.2<br>(0.3 – 3.2) | 6.2<br>(1.5 – 16.7) | 9.2<br>(2.2 – 22.9) |
| France | 7.1<br>(2.5 – 15.2) | 12.3<br>(4.0 – 27.1) | 16.3<br>(5.7 – 32.7) |
| Gabon | 5.7<br>(2.5 – 10.5) | 21.3<br>(13.2 – 31.0) | 19.9<br>(12.5 – 28.5) |
| Georgia | 0.0<br>(0.0 – <0.1) | 0.3<br>(0.1 – 0.5) | 2.6<br>(1.4 – 4.5) |
| Germany | 4.1<br>(1.3 – 9.8) | 10.0<br>(2.9 – 23.7) | 18.4<br>(6.2 – 38.0) |
| Ghana | 5.7<br>(2.5 – 10.6) | 10.0<br>(4.9 – 17.2) | 7.9<br>(3.9 – 13.6) |
| Greece | 0.8<br>(0.3 – 1.9) | 2.7<br>(0.6 – 7.6) | 5.2<br>(1.3 – 13.1) |
| Greenland | 6.1<br>(2.7 – 11.3) | 11.6<br>(5.1 – 21.8) | 17.5<br>(7.4 – 32.1) |
| Grenada | 0.7<br>(0.3 – 1.3) | 1.2<br>(0.6 – 2.2) | 1.1<br>(0.6 – 1.9) |
| Guam | 0.3<br>(0.1 – 0.6) | 0.3<br>(0.1 – 0.6) | 0.4<br>(0.2 – 0.8) |
| Guatemala | 0.2<br>(0.1 – 0.4) | 1.5<br>(0.7 – 2.6) | 2.2<br>(1.1 – 3.7) |

| Country | 1990 | 2005 | 2017 |
|---|---|---|---|
| Guinea | 1.3 (0.5 – 2.7) | 5.8 (2.5 – 11.0) | 5.6 (2.5 – 10.2) |
| Guinea-Bissau | 1.9 (0.7 – 3.9) | 9.8 (4.5 – 17.0) | 10.3 (5.1 – 17.2) |
| Guyana | 1.9 (0.8 – 3.5) | 7.5 (4.0 – 12.7) | 11.0 (6.6 – 17.3) |
| Haiti | 6.4 (3.0 – 11.4) | 8.7 (4.3 – 14.8) | 8.9 (4.5 – 15.3) |
| Honduras | 0.2 (0.1 – 0.5) | 0.6 (0.3 – 1.0) | 1.2 (0.6 – 2.1) |
| Hungary | 0.6 (0.3 – 1.2) | 0.6 (0.3 – 1.1) | 0.8 (0.4 – 1.6) |
| Iceland | 1.9 (0.5 – 5.1) | 5.4 (1.2 – 14.0) | 9.5 (2.4 – 23.2) |
| India | 0.0 (0.0 – 0.1) | 1.4 (0.7 – 2.6) | 1.3 (0.6 – 2.3) |
| Indonesia | 0.0 (0.0 – <0.1) | 0.3 (0.2 – 0.5) | 1.0 (0.5 – 1.7) |
| Iran | 0.0 (0.0 – <0.1) | 0.2 (0.1 – 0.3) | 0.7 (0.4 – 1.2) |
| Iraq | 0.0 (0.0 – <0.1) | 0.0 (0.0 – <0.1) | 0.0 (0.0 – 0.1) |
| Ireland | 0.7 (0.2 – 1.6) | 6.4 (1.7 – 16.1) | 10.3 (2.9 – 25.4) |
| Israel | 1.2 (0.5 – 2.4) | 6.7 (2.4 – 13.6) | 12.1 (4.8 – 23.8) |
| Italy | 5.4 (2.0 – 11.2) | 13.3 (5.6 – 24.9) | 19.9 (9.0 – 34.9) |
| Jamaica | 1.4 (0.6 – 2.6) | 4.0 (2.1 – 6.7) | 7.2 (4.0 – 12.0) |
| Japan | 0.2 (0.1 – 0.4) | 1.1 (0.4 – 2.5) | 2.0 (0.8 – 4.4) |
| Jordan | 0.0 (0.0 – <0.1) | 0.0 (0.0 – 0.1) | 0.1 (0.1 – 0.2) |
| Kazakhstan | 0.1 (0.1 – 0.3) | 0.5 (0.2 – 0.8) | 1.3 (0.7 – 2.3) |
| Kenya | 8.4 (4.6 – 13.5) | 22.6 (13.9 – 32.7) | 19.8 (12.2 – 28.8) |
| Kiribati | 0.0 (0.0 – 0.1) | 0.0 (0.0 – 0.1) | 0.0 (0.0 – 0.1) |
| Kuwait | 0.1 (0.0 – 0.1) | 0.1 (0.1 – 0.2) | 0.2 (0.1 – 0.4) |
| Kyrgyzstan | 0.1 (0.0 – 0.1) | 0.4 (0.2 – 0.8) | 1.3 (0.6 – 2.3) |
| Laos | 0.0 (0.0 – <0.1) | 0.8 (0.2 – 2.1) | 1.3 (0.5 – 2.9) |
| Latvia | 0.4 (0.2 – 0.8) | 1.7 (1.0 – 2.9) | 4.3 (2.3 – 6.9) |

| Country | 1990 | 2005 | 2017 |
|---|---|---|---|
| Lebanon | 0.2<br>(0.1 – 0.4) | 0.3<br>(0.1 – 0.5) | 0.5<br>(0.2 – 0.9) |
| Lesotho | 13.7<br>(6.6 – 22.7) | 42.3<br>(29.6 – 55.2) | 46.5<br>(34.9 – 58.0) |
| Liberia | 2.1<br>(0.7 – 4.7) | 8.1<br>(3.7 – 14.5) | 5.0<br>(2.1 – 9.4) |
| Libya | 0.0<br>(0.0 – 0.1) | 0.2<br>(0.1 – 0.4) | 0.4<br>(0.2 – 0.9) |
| Lithuania | 0.2<br>(0.1 – 0.3) | 0.9<br>(0.5 – 1.5) | 1.7<br>(0.9 – 2.8) |
| Luxembourg | 3.4<br>(0.9 – 8.8) | 9.9<br>(2.6 – 23.6) | 15.8<br>(4.7 – 34.6) |
| Macedonia | 0.0<br>(0.0 – <0.1) | 0.1<br>(0.0 – 0.1) | 0.2<br>(0.1 – 0.4) |
| Madagascar | 0.0<br>(0.0 – <0.1) | 2.0<br>(0.7 – 4.7) | 1.5<br>(0.5 – 3.4) |
| Malawi | 19.8<br>(11.3 – 29.8) | 32.7<br>(21.7 – 45.0) | 28.7<br>(18.8 – 39.7) |
| Malaysia | 0.2<br>(0.1 – 0.4) | 2.5<br>(1.3 – 4.4) | 3.6<br>(1.8 – 6.2) |
| Maldives | 0.0<br>(0.0 – <0.1) | 0.0<br>(0.0 – <0.1) | 0.0<br>(0.0 – 0.1) |
| Mali | 1.1<br>(0.4 – 2.4) | 5.0<br>(2.1 – 9.6) | 4.1<br>(1.7 – 7.9) |
| Malta | 0.9<br>(0.3 – 1.9) | 4.2<br>(1.1 – 11.4) | 9.4<br>(2.5 – 22.5) |
| Marshall Islands | 0.1<br>(0.0 – 0.2) | 0.2<br>(0.1 – 0.4) | 0.5<br>(0.2 – 1.2) |
| Mauritania | 0.3<br>(0.1 – 0.8) | 0.2<br>(0.0 – 0.4) | 0.2<br>(0.1 – 0.6) |
| Mauritius | 0.1<br>(0.0 – 0.1) | 1.5<br>(0.7 – 2.5) | 6.5<br>(3.5 – 10.8) |
| Mexico | 0.5<br>(0.3 – 0.9) | 1.9<br>(1.0 – 3.1) | 3.8<br>(2.1 – 6.2) |
| Moldova | 0.2<br>(0.1 – 0.4) | 1.2<br>(0.6 – 2.3) | 3.7<br>(1.9 – 6.5) |
| Mongolia | 0.0<br>(0.0 – <0.1) | 0.0<br>(0.0 – 0.1) | 0.2<br>(0.1 – 0.5) |
| Montenegro | 0.0<br>(0.0 – 0.1) | 0.2<br>(0.1 – 0.3) | 0.6<br>(0.3 – 1.0) |
| Morocco | 0.1<br>(0.0 – 0.1) | 0.4<br>(0.2 – 0.9) | 0.7<br>(0.3 – 1.4) |
| Mozambique | 5.7<br>(2.5 – 10.7) | 22.7<br>(13.5 – 33.9) | 30.7<br>(20.4 – 41.8) |
| Myanmar | 0.1<br>(0.0 – 0.2) | 3.6<br>(1.7 – 6.7) | 4.1<br>(2.0 – 7.4) |
| Namibia | 13.5<br>(7.0 – 21.9) | 38.6<br>(27.4 – 50.4) | 40.8<br>(30.6 – 51.6) |

| Country | 1990 | 2005 | 2017 |
|---|---|---|---|
| Nepal | 0.0<br>(0.0 – <0.1) | 1.1<br>(0.4 – 2.5) | 1.2<br>(0.5 – 2.5) |
| Netherlands | 5.0<br>(1.5 – 12.2) | 11.8<br>(3.3 – 27.3) | 17.8<br>(6.0 – 37.2) |
| New Zealand | 2.0<br>(0.8 – 4.0) | 3.9<br>(1.5 – 8.1) | 5.5<br>(2.2 – 10.9) |
| Nicaragua | 0.1<br>(0.0 – 0.1) | 0.6<br>(0.3 – 1.2) | 2.8<br>(1.5 – 4.8) |
| Niger | 0.5<br>(0.2 – 1.1) | 3.3<br>(1.3 – 6.5) | 1.4<br>(0.5 – 2.9) |
| Nigeria | 2.8<br>(1.1 – 5.7) | 11.7<br>(5.8 – 20.0) | 9.5<br>(4.7 – 16.8) |
| North Korea | 0.1<br>(0.0 – 0.2) | 0.4<br>(0.1 – 1.0) | 0.9<br>(0.3 – 2.1) |
| Northern Mariana Islands | 0.1<br>(0.0 – 0.1) | 0.1<br>(0.0 – 0.2) | 0.2<br>(0.1 – 0.3) |
| Norway | 1.7<br>(0.5 – 4.2) | 7.6<br>(2.2 – 17.7) | 12.2<br>(3.8 – 26.5) |
| Oman | 0.0<br>(0.0 – 0.1) | 0.5<br>(0.3 – 0.9) | 1.8<br>(1.0 – 3.0) |
| Pakistan | 0.0<br>(0.0 – 0.1) | 0.1<br>(0.0 – 0.2) | 0.4<br>(0.1 – 0.9) |
| Palestine | 0.0<br>(0.0 – <0.1) | 0.0<br>(0.0 – <0.1) | 0.0<br>(0.0 – 0.1) |
| Panama | 1.2<br>(0.6 – 2.2) | 4.1<br>(2.3 – 6.8) | 7.5<br>(4.5 – 11.4) |
| Papua New Guinea | 0.0<br>(0.0 – <0.1) | 1.5<br>(0.6 – 3.1) | 1.8<br>(0.7 – 3.5) |
| Paraguay | 0.1<br>(0.1 – 0.2) | 1.3<br>(0.7 – 2.3) | 3.8<br>(2.1 – 6.3) |
| Peru | 0.4<br>(0.2 – 0.7) | 1.6<br>(0.9 – 2.7) | 3.6<br>(2.0 – 5.9) |
| Philippines | 0.3<br>(0.1 – 0.6) | 0.7<br>(0.3 – 1.2) | 1.4<br>(0.7 – 2.4) |
| Poland | 0.1<br>(0.0 – 0.1) | 0.7<br>(0.4 – 1.3) | 1.4<br>(0.7 – 2.6) |
| Portugal | 3.2<br>(1.5 – 5.7) | 26.1<br>(14.5 – 40.6) | 39.9<br>(24.5 – 56.5) |
| Puerto Rico | 5.1<br>(2.8 – 8.0) | 2.9<br>(1.6 – 4.9) | 1.4<br>(0.8 – 2.4) |
| Qatar | 0.2<br>(0.1 – 0.3) | 0.2<br>(0.1 – 0.4) | 0.2<br>(0.1 – 0.3) |
| Romania | 0.1<br>(0.0 – 0.1) | 0.8<br>(0.4 – 1.3) | 2.2<br>(1.2 – 3.7) |
| Russian Federation | 0.6<br>(0.3 – 1.0) | 2.3<br>(1.3 – 3.6) | 9.1<br>(5.5 – 13.8) |
| Rwanda | 3.2<br>(1.0 – 7.1) | 8.8<br>(3.2 – 18.1) | 8.7<br>(2.6 – 17.2) |

| Country | 1990 | 2005 | 2017 |
|---|---|---|---|
| Saint Lucia | 0.5<br>(0.3 – 1.0) | 1.0<br>(0.5 – 1.8) | 1.0<br>(0.5 – 1.6) |
| Saint Vincent and the Grenadines | 1.5<br>(0.7 – 2.7) | 3.4<br>(1.9 – 5.9) | 3.0<br>(1.6 – 5.0) |
| Samoa | 0.1<br>(0.0 – 0.3) | 0.2<br>(0.1 – 0.5) | 0.5<br>(0.2 – 1.2) |
| Sao Tome and Principe | 0.0<br>(0.0 – 0.1) | 0.1<br>(0.0 – 0.2) | 0.2<br>(0.1 – 0.4) |
| Saudi Arabia | 0.1<br>(0.0 – 0.1) | 0.2<br>(0.1 – 0.4) | 0.4<br>(0.2 – 0.7) |
| Senegal | 0.6<br>(0.2 – 1.3) | 3.9<br>(1.8 – 7.2) | 3.3<br>(1.5 – 6.1) |
| Serbia | 0.1<br>(0.0 – 0.2) | 0.5<br>(0.2 – 0.8) | 1.1<br>(0.5 – 1.9) |
| Seychelles | 0.3<br>(0.2 – 0.6) | 1.1<br>(0.6 – 1.9) | 1.4<br>(0.8 – 2.4) |
| Sierra Leone | 0.1<br>(0.0 – 0.2) | 5.8<br>(2.5 – 10.7) | 4.9<br>(2.1 – 9.3) |
| Singapore | 0.8<br>(0.3 – 1.5) | 4.8<br>(1.9 – 10.0) | 5.9<br>(2.3 – 12.2) |
| Slovakia | 0.0<br>(0.0 – <0.1) | 0.1<br>(0.0 – 0.2) | 0.3<br>(0.1 – 0.5) |
| Slovenia | 0.1<br>(0.0 – 0.1) | 0.2<br>(0.1 – 0.4) | 0.4<br>(0.2 – 0.8) |
| Solomon Islands | 0.1<br>(0.0 – 0.2) | 0.1<br>(0.0 – 0.4) | 0.3<br>(0.1 – 0.8) |
| Somalia | 0.4<br>(0.1 – 1.0) | 2.0<br>(0.6 – 4.7) | 1.1<br>(0.3 – 2.7) |
| South Africa | 0.9<br>(0.4 – 1.9) | 36.7<br>(24.1 – 49.4) | 41.2<br>(25.9 – 52.9) |
| South Korea | 0.2<br>(0.1 – 0.4) | 2.8<br>(1.0 – 6.0) | 4.5<br>(1.8 – 9.5) |
| South Sudan | 1.1<br>(0.3 – 2.8) | 4.3<br>(1.3 – 9.9) | 3.8<br>(1.1 – 8.9) |
| Spain | 11.1<br>(3.8 – 23.2) | 21.6<br>(9.1 – 39.5) | 27.4<br>(12.0 – 46.3) |
| Sri Lanka | 0.0<br>(0.0 – 0.1) | 0.1<br>(0.0 – 0.1) | 0.2<br>(0.1 – 0.3) |
| Sudan | 0.3<br>(0.1 – 0.5) | 1.3<br>(0.5 – 2.5) | 1.8<br>(0.8 – 3.4) |
| Suriname | 1.5<br>(0.8 – 2.9) | 6.0<br>(3.4 – 9.9) | 7.8<br>(4.6 – 12.2) |
| Swaziland | 1.4<br>(0.5 – 2.9) | 41.4<br>(29.4 – 53.8) | 45.5<br>(34.9 – 56.4) |
| Sweden | 1.7<br>(0.5 – 3.9) | 3.7<br>(0.9 – 9.1) | 5.1<br>(1.3 – 12.4) |
| Switzerland | 8.7<br>(2.7 – 19.4) | 18.9<br>(6.7 – 37.4) | 24.1<br>(9.0 – 44.5) |

| Country | 1990 | 2005 | 2017 |
|---|---|---|---|
| Syria | 0.0<br>(0.0 – <0.1) | 0.0<br>(0.0 – <0.1) | 0.1<br>(0.0 – 0.1) |
| Taiwan | 0.0<br>(0.0 – 0.1) | 0.3<br>(0.2 – 0.5) | 0.7<br>(0.4 – 1.2) |
| Tajikistan | 0.1<br>(0.0 – 0.1) | 0.3<br>(0.1 – 0.5) | 0.6<br>(0.3 – 1.1) |
| Tanzania | 17.1<br>(9.5 – 26.0) | 17.9<br>(10.2 – 27.4) | 16.1<br>(9.1 – 24.9) |
| Thailand | 1.5<br>(0.7 – 2.7) | 7.3<br>(4.2 – 11.9) | 11.1<br>(6.6 – 17.1) |
| The Bahamas | 6.6<br>(3.6 – 10.3) | 10.0<br>(5.8 – 15.3) | 14.9<br>(9.5 – 21.8) |
| The Gambia | 0.5<br>(0.2 – 1.0) | 6.7<br>(3.2 – 12.0) | 7.4<br>(3.6 – 12.7) |
| Timor-Leste | 0.7<br>(0.2 – 1.7) | 1.8<br>(0.6 – 3.9) | 1.7<br>(0.6 – 3.5) |
| Togo | 2.2<br>(0.8 – 4.4) | 12.4<br>(6.1 – 21.1) | 8.7<br>(4.0 – 15.2) |
| Tonga | 0.0<br>(0.0 – 0.1) | 0.1<br>(0.0 – 0.1) | 0.1<br>(0.1 – 0.2) |
| Trinidad and Tobago | 2.0<br>(1.0 – 3.5) | 7.3<br>(4.3 – 11.3) | 10.0<br>(6.0 – 15.4) |
| Tunisia | 0.0<br>(0.0 – 0.1) | 0.2<br>(0.1 – 0.4) | 0.6<br>(0.3 – 1.2) |
| Turkey | 0.0<br>(0.0 – <0.1) | 0.0<br>(0.0 – 0.1) | 0.1<br>(0.1 – 0.2) |
| Turkmenistan | 0.2<br>(0.1 – 0.4) | 0.5<br>(0.2 – 0.8) | 0.4<br>(0.2 – 0.7) |
| Uganda | 15.6<br>(6.9 – 27.7) | 12.2<br>(5.1 – 22.7) | 11.6<br>(4.3 – 22.1) |
| Ukraine | 1.0<br>(0.5 – 1.7) | 4.6<br>(2.5 – 7.6) | 7.2<br>(4.1 – 11.6) |
| United Arab Emirates | 0.0<br>(0.0 – 0.1) | 0.3<br>(0.1 – 0.6) | 0.6<br>(0.2 – 1.4) |
| United Kingdom | 1.8<br>(0.6 – 4.2) | 8.7<br>(2.5 – 19.9) | 14.7<br>(4.7 – 30.7) |
| United States | 12.0<br>(6.2 – 19.8) | 18.5<br>(9.1 – 30.7) | 24.2<br>(12.1 – 39.2) |
| Uruguay | 1.7<br>(0.8 – 2.9) | 7.5<br>(3.9 – 12.6) | 12.4<br>(6.1 – 21.8) |
| Uzbekistan | 0.1<br>(0.0 – 0.1) | 0.5<br>(0.2 – 0.9) | 1.0<br>(0.5 – 1.8) |
| Vanuatu | 0.1<br>(0.0 – 0.2) | 0.1<br>(0.0 – 0.3) | 0.3<br>(0.1 – 0.8) |
| Venezuela | 0.4<br>(0.2 – 0.7) | 3.3<br>(1.8 – 5.4) | 6.7<br>(3.8 – 10.6) |
| Vietnam | 0.2<br>(0.1 – 0.5) | 2.2<br>(1.1 – 4.0) | 3.9<br>(2.0 – 6.6) |

| Country | 1990 | 2005 | 2017 |
|---|---|---|---|
| Virgin Islands, U.S. | 1.9<br>(1.0 – 3.1) | 2.5<br>(1.4 – 4.1) | 2.9<br>(1.6 – 4.6) |
| Yemen | 0.1<br>(0.0 – 0.1) | 0.1<br>(0.0 – 0.2) | 0.2<br>(0.1 – 0.4) |
| Zambia | 12.5<br>(5.0 – 23.6) | 18.7<br>(8.1 – 32.8) | 17.9<br>(7.2 – 31.1) |
| Zimbabwe | 27.6<br>(18.5 – 37.6) | 37.5<br>(26.6 – 49.1) | 34.5<br>(24.6 – 44.8) |

# C. Source Appendix

## C1. Incidence data sources


1. Arshad MM, Wilkins MJ, Downes FP, Rahbar MH, Erskine RJ, Boulton ML, et al. Epidemiologic attributes of invasive non-typhoidal *Salmonella* infections in Michigan, 1995–2001. *Int J Infect Dis* 2008;12(2):176–82.

2. Berkley JA, Lowe BS, Mwangi I, Williams T, Bauni E, Mwarumba S, et al. Bacteremia among children admitted to a rural hospital in Kenya. *N Engl J Med* 2005;352(1):39–47.

3. Chen PL, Li CY, Hsieh TH, Chang CM, Lee HC, Lee NY, et al. Epidemiology, disease spectrum and economic burden of non-typhoidal *Salmonella* infections in Taiwan, 2006–2008. *Epidemiol Infect* 2012;140(12):2256–63.

4. Cheng LH, Crim SM, Cole CR, Shane AL, Henao OL, Mahon BE. Epidemiology of Infant Salmonellosis in the United States, 1996-2008: A Foodborne Diseases Active Surveillance Network Study. *J Pediatric Infect Dis Soc* 2013;2(3):232–9.

5. Czerwińrski M. [Salmonellosis in Poland in 2004]. *Przegl Epidemiol* 2006;60(3):429–40.

6. Czerwiński M, Gonera E. [Salmonellosis in Poland in 2003]. *Przegl Epidemiol* 2005;59(2):253–62.

7. Enwere G, Biney E, Cheung Y, Zaman SM, Okoko B, Oluwalana C, et al. Epidemiologic and clinical characteristics of community-acquired invasive bacterial infections in children aged 2–29 months in The Gambia. *Pediatr Infect Dis J* 2006;25(8):700–705.

8. Feasey NA, Houston A, Mukaka M, Komrower D, Mwalukomo T, Tenthani L, et al. A Reduction in Adult Blood Stream Infection and Case Fatality at a Large African Hospital following Antiretroviral Therapy Roll-Out. *PLoS One* 2014;9(3):e92226.

9. Feasey NA, Masesa C, Jassi C, Faragher EB, Mallewa J, Mallewa M, et al. Three Epidemics of Invasive Multidrug-Resistant *Salmonella* Bloodstream Infection in Blantyre, Malawi, 1998–2014. *Clin Infect Dis* 2015;61( 4):S363–71.

10. Gonera E. [Salmonellosis in 1997]. *Przegl Epidemiol* 1999;53(1–2):83–91.

11. Gonera E. [Salmonellosis in Poland in 2000]. *Przegl Epidemiol* 2002;56(2):275–84.

12. Gonera E. [Salmonellosis in Poland in 2001]. *Przegl Epidemiol* 2003;57(1):67–76.

13. Gonera E, Czerwiński M. [Salmonellosis in Poland in 2002]. *Przegl Epidemiol* 2004;58:67–76.

14. Gradel KO, Schønheyder HC, Pedersen L, Thomsen RW, Nørgaard M, Nielsen H. Incidence and prognosis of nontyphoid *Salmonella* bacteraemia in Denmark: a 10-year county-based follow-up study. *Eur J Clin Microbiol Infect Dis* 2006;25(3):151–8.

15. Guiraud I, Post A, Diallo SN, Lompo P, Maltha J, Thriemer K, et al. Population-based incidence, seasonality and serotype distribution of invasive salmonellosis among children in Nanoro, rural Burkina Faso. *PLoS One* 2017;12(7):e0178577.

16. Jones TF, Ingram LA, Cieslak PR, Vugia DJ, Tobin-D'Angelo M, Hurd S, et al. Salmonellosis Outcomes Differ Substantially by Serotype. *J Infect Dis* 2008;198(1):109–14.

17. Keddy KH, Takuva S, Musekiwa A, Puren AJ, Sooka A, Karstaedt A, et al. An association between decreasing incidence of invasive non-typhoidal salmonellosis and increased use of antiretroviral therapy, Gauteng Province, South Africa, 2003–2013. *PLoS One* 2017;12(3):e0173091.

18. Khan MI, Ochiai RL, Von Seidlein L, Dong B, Bhattacharya SK, Agtini MD, et al. Non-typhoidal *Salmonella* rates in febrile children at sites in five Asian countries: Non-typhoidal *Salmonella* in Asia. *Trop Med Int Health* 2010;15(8):960–3.

19. Laupland KB, Schønheyder HC, Kennedy KJ, Lyytikäinen O, Valiquette L, Galbraith J, et al. *Salmonella* enterica bacteraemia: a multi-national population-based cohort study. *BMC Infect Dis* 2010;10(1):95.

20. Mandomando I, Bassat Q, Sigaúque B, Massora S, Quintó L, Ácacio S, et al. Invasive *Salmonella* Infections Among Children From Rural Mozambique, 2001–2014. *Clin Infect Dis* 2015;61(suppl 4):S339–45.

21. Marks F, von Kalckreuth V, Aaby P, Adu-Sarkodie Y, El Tayeb MA, Ali M, et al. Incidence of invasive *Salmonella* disease in sub-Saharan Africa: a multicentre population-based surveillance study. *Lancet Glob Health* 2017;5(3):e310–e323.

22. Marzel A, Desai PT, Nissan I, Schorr YI, Suez J, Valinsky L, et al. Integrative Analysis of Salmonellosis in Israel Reveals Association of *Salmonella* enterica Serovar 9,12:l,v:- with Extraintestinal Infections, Dissemination of Endemic S. enterica Serovar Typhimurium DT104 Biotypes, and Severe Underreporting of Outbreaks. *J Clin Microb* 2014;52(6):2078–88.

23. Mayanja BN, Todd J, Hughes P, Van der Paal L, Mugisha JO, Atuhumuza E, et al. Septicaemia in a population-based HIV clinical cohort in rural Uganda, 1996-2007: incidence, aetiology, antimicrobial drug resistance and impact of antiretroviral therapy: Septicaemia in a rural Ugandan HIV cohort. *Trop Med Int Health* 2010;15(6):697–705.

24. Mtove G, Amos B, Nadjm B, Hendriksen IC, Dondorp AM, Mwambuli A, et al. Decreasing incidence of severe malaria and community-acquired bacteraemia among hospitalized children in Muheza, north-eastern Tanzania, 2006-2010. *Malar J* 2011;10(1):320.

25. Muthumbi E, Morpeth SC, Ooko M, Mwanzu A, Mwarumba S, Mturi N, et al. Invasive Salmonellosis in Kilifi, Kenya. *Clin Infect Dis* 2015;61(suppl 4):S290–301.

26. Nielsen MV, Sarpong N, Krumkamp R, Dekker D, Loag W, Amemasor S, et al. Incidence and Characteristics of Bacteremia among Children in Rural Ghana. Wertheim HFL, editor. *PLoS One* 2012;7(9):e44063.

27. Oneko M, Kariuki S, Muturi-Kioi V, Otieno K, Otieno VO, Williamson JM, et al. Emergence of Community-Acquired, Multidrug-Resistant Invasive Nontyphoidal *Salmonella* Disease in Rural Western Kenya, 2009–2013. *Clinical Infect Dis* 2015;61(suppl 4):S310–6.

28. Orysiak P, Czarkowski MP, Sadkowska-Todys M. Salmonelozy w Polsce w 2008 roku. *Przegl Epidemiol* 2010;64:221–230.

29. Schønheyder H, Ejlertsen T. Survey of extraintestinal nontyphoid *Salmonella* infections in a Danish region. *APMIS* 1995;103(7–8):686–688.

30. Tabu C, Breiman RF, Ochieng B, Aura B, Cosmas L, Audi A, et al. Differing Burden and Epidemiology of Non-Typhi *Salmonella* Bacteremia in Rural and Urban Kenya, 2006–2009. *PLoS One* 2012;7(2):e31237.

31. Verani JR, Toroitich S, Auko J, Kiplang'at S, Cosmas L, Audi A, et al. Burden of Invasive Nontyphoidal *Salmonella* Disease in a Rural and Urban Site in Kenya, 2009–2014. *Clin Infect Dis* 2015;61(suppl 4):S302–9.

32. Vugia DJ, Samuel M, Farley MM, Marcus R, Shiferaw B, Shallow S, et al. Invasive *Salmonella* infections in the United States, FoodNet, 1996–1999: incidence, serotype distribution, and outcome. *Clin Infect Dis* 2004;38(suppl 3):S149–S156.

33. Williams TN, Uyoga S, Macharia A, Ndila C, McAuley CF, Opi DH, et al. Bacteraemia in Kenyan children with sickle-cell anaemia: a retrospective cohort and case–control study. *Lancet* 2009;374(9698):1364–1370.

34. Yagupsky P, Maimon N, Dagan R. Increasing incidence of non-typhi *Salmonella* bacteremia among children living in southern Israel. *Int J Infect Dis* 2002;6(2):94–97.

35. Zaidenstein R, Peretz C, Nissan I, Reisfeld A, Yaron S, Agmon V, et al. The epidemiology of extraintestinal non-typhoid *Salmonella* in Israel: the effects of patients' age and sex. *Eur J Clin Microbiol Infect Dis* 2010 Sep;29(9):1103–9.

### C2. Case fatality data sources

1. Berkowitz FE. Bacteremia in hospitalized black South African children: a one-year study emphasizing nosocomial bacteremia and bacteremia in severely malnourished children. *Am J Dis Child* 1984;138(6):551–556.

2. Brown M, Eykyn SJ. Non-typhoidal *Salmonella* Bacteraemia Without Gastroenteritis: a Marker of Underlying Immunosuppression. Review of Casesat St. Thomas' Hospital 1970–1999. *J Infect* 2000;41(3):256–9.

3. Chen PL, Li CY, Hsieh TH, Chang CM, Lee HC, Lee NY, et al. Epidemiology, disease spectrum and economic burden of non-typhoidal *Salmonella* infections in Taiwan, 2006–2008. *Epidemiol Infect* 2012;140(12):2256–63.

4. Cheng LH, Crim SM, Cole CR, Shane AL, Henao OL, Mahon BE. Epidemiology of Infant Salmonellosis in the United States, 1996-2008: A Foodborne Diseases Active Surveillance Network Study. *J Pediatric Infect Dis Soc* 2013;2(3):232–9.

5. Cheng M-W, Lee C-M, Wang N-Y, Wu AY, Lin C-C, Weng L-C, et al. Clinical characteristics in adult patients with Salmonella bacteremia and analysis of ciprofloxacin-nonsusceptible isolates. *J Microbiol Immunol Infect* 2015;48(6):692–8.

6. Dhanoa A, Fatt QK. Non-typhoidal *Salmonella* bacteraemia: Epidemiology, clinical characteristics and its' association with severe immunosuppression. *Ann Clin Microbiol Antimicrob* 2009;8(1):15.

7. Falay D, Kuijpers LMF, Phoba M-F, De Boeck H, Lunguya O, Vakaniaki E, et al. Microbiological, clinical and molecular findings of non-typhoidal *Salmonella* bloodstream infections associated with malaria, Oriental Province, Democratic Republic of the Congo. *BMC Infect Dis* 2016 Dec;16(1).

8. Feasey NA, Houston A, Mukaka M, Komrower D, Mwalukomo T, Tenthani L, et al. A Reduction in Adult Blood Stream Infection and Case Fatality at a Large African Hospital following Antiretroviral Therapy Roll-Out. *PLoS One* 2014;9(3):e92226.

9. Galofré J, Moreno A, Mensa J, Miró JM, Gatell JM, Almela M, et al. Analysis of factors influencing the outcome and development of septic metastasis or relapse in *Salmonella* bacteremia. *Clin Infect Dis* 1994;18(6):873–878.

10. Gordon MA, Banda HT, Gondwe M, Gordon SB, Boeree MJ, Walsh AL, et al. Non-typhoidal *Salmonella* bacteraemia among HIV-infected Malawian adults: high mortality and frequent recrudescence. *AIDS* 2002;16(12):1633–1641.

11. Gradel KO, Schønheyder HC, Pedersen L, Thomsen RW, Nørgaard M, Nielsen H. Incidence and prognosis of nontyphoid *Salmonella* bacteraemia in Denmark: a 10-year county-based follow-up study. *Eur J Clin Microb Infect Dis* 2006;25(3):151–8.

12. Graham SM, Walsh AL, Molyneux EM, Phiri AJ, Molyneux ME. Clinical presentation of non-typhoidal *Salmonella* bacteraemia in Malawian children. *Trans R Soc Trop Med* 2000;94(3):310–314.

13. Huang C-F, Chen P-L, Liu M-F, Lee C-C, Lee N-Y, Chang C-M, et al. Nontyphoidal *Salmonella* bacteremia in patients with connective tissue diseases. J *Microbiol Immunol Infect* 2012;45(5):350–5.

14. Ispahani P, Slack RCB. Enteric fever and other extraintestinal salmonellosis in University Hospital, Nottingham, UK, between 1980 and 1997. *Eur J Clin Microb Infect Dis* 2000;19(9):679–687.

15. Keddy KH, Musekiwa A, Sooka A, Karstaedt A, Nana T, Seetharam S, et al. Clinical and microbiological features of invasive nontyphoidal *Salmonella* associated with HIV-infected patients, Gauteng Province, South Africa: *Medicine* 2017;96(13):e6448.

16. Kiratisin P. Bacteraemia due to non-typhoidal *Salmonella* in Thailand: clinical and microbiological analysis. *Trans R Soc Trop Med* 2008;102(4):384–8.

17. Koch K, Kristensen B, Holt HM, Ethelberg S, Mølbak K are, Schønheyder HC. International travel and the risk of hospitalization with non-typhoidal *Salmonella* bacteremia. A Danish population-based cohort study, 1999-2008. *BMC Infect Dis* 2011;11(1):277.

18. Lee SC, Yang PH, Shieh WB, Lasserre R. Bacteremia due to non-typhi *Salmonella*: analysis of 64 cases and review. *Clin Infect Dis* 1994;19(4):693–6.

19. Lee WS, Puthucheary SD, Parasakthi N. Extra-intestinal non-typhoidal *Salmonella* infections in children. *Ann Trop Paediatr* 2000;20(2):125–9.

20. Lepage P, Bogaerts J, Van Goethem C, Ntahorutaba M, Nsengumuremyi F, Hitimana D, et al. Community-acquired bacteraemia in African children. *Lancet* 1987;329(8548):1458–1461.

21. Li C-W, Chen P-L, Lee N-Y, Lee H-C, Chang C-M, Lee C-C, et al. Non-typhoidal *Salmonella* bacteremia among adults: An adverse prognosis in patients with malignancy. J *Microbiol Immunol Infect* J *Microbiol Immunol Infect* 2012;45(5):343–9.

22. Mandomando I, Bassat Q, Sigaúque B, Massora S, Quintó L, Ácacio S, et al. Invasive *Salmonella* Infections Among Children From Rural Mozambique, 2001–2014. *Clin Infect Dis* 2015;61(suppl 4):S339–45.

23. Muthumbi E, Morpeth SC, Ooko M, Mwanzu A, Mwarumba S, Mturi N, et al. Invasive Salmonellosis in Kilifi, Kenya. *Clin Infect Dis* 2015;61(suppl 4):S290–301.

24. Nesbitt A, Mirza NB. *Salmonella* septicaemias in Kenyan children. *J Trop Pediatr* 1989;35(1):35–9.

25. Parry CM, Thomas S, Aspinall EJ, Cooke RPD, Rogerson SJ, Harries AD, et al. A retrospective study of secondary bacteraemia in hospitalised adults with community acquired non-typhoidal *Salmonella* gastroenteritis. *BMC Infect Dis* 2013;13:107.

26. Phoba M-F, Boeck H, Ifeka BB, Dawili J, Lunguya O, Vanhoof R, et al. Epidemic increase in *Salmonella* bloodstream infection in children, Bwamanda, the Democratic Republic of Congo. *Eur J Clin Microb Infect Dis* 2014;33(1):79–87.

27. Phu Huong Lan N, Le Thi Phuong T, Nguyen Huu H, Thuy L, Mather AE, Park SE, et al. Invasive Non-typhoidal *Salmonella* Infections in Asia: Clinical Observations, Disease Outcome and Dominant Serovars from an Infectious Disease Hospital in Vietnam. *PLoS Negl Trop Dis* 2016;10(8):e0004857.

28. Ramos JM, García-Corbeira P, Aguado JM, Arjona R, Alés JM, Soriano F. Clinical significance of primary vs. secondary bacteremia due to nontyphoid *Salmonella* in patients without AIDS. *Clin Infect Dis* 1994;19(4):777–80.

29. Ryu C-B, Lee M-L, Namgoong EK, Kee SY, Lee WG, Woo JH. Bacteremia With Nontyphi *Salmonella* and Therapeutic Implication. *Korean J Intern Med* 1995;10(2):146–9.

30. Seydi M, Soumare M, Sow AI, Diop SA, Sow I, Dieng AB, et al. Étude descriptive des bactériémies à *Salmonella* non typhi chez les patients infectés par le VIH au CHU de Dakar (Sénégal). *Med Mal Infect* 2008;38(1):25–8.

31. Shimoni Z, Pitlik S, Leibovici L, Samra Z, Konigsberger H, Drucker M, et al. Nontyphoid *Salmonella* bacteremia: age-related differences in clinical presentation, bacteriology, and outcome. *Clin Infect Dis* 1999;28(4):822–7.

32. Sirinavin S, Chiemchanya S, Vorachit M. Systemic nontyphoidal *Salmonella* infection in normal infants in Thailand. *Pediatr Infect Dis J* 2001;20(6):581–7.

33. V. J. Pithie AD. *Salmonella* and *Shigella* Bacteraemia in Zimbabwe. *Cent Afr J Med* 1993;39(6):110–2.

34. Wang J-Y, Hwang J-J, Hsu C-N, Lin L-C, Hsueh P-R. Bacteraemia due to ciprofloxacin-resistant *Salmonella* enterica serotype Choleraesuis in adult patients at a university hospital in Taiwan, 1996–2004. *Epidemiol Infect* 2006;134(05):977.

35. Zaidi E, Bachur R, Harper M. Non-typhi *Salmonella* bacteremia in children. *Pediatr Infect Dis J* 1999;18(12):1073–1077.

### C3. HIV coinfection data sources

1. Berkley JA, Lowe BS, Mwangi I, Williams T, Bauni E, Mwarumba S, et al. Bacteremia among children admitted to a rural hospital in Kenya. *N Engl J Med* 2005;352(1):39–47.

2. Gruenewald R, Blum S, Chan J. Relationship between human immunodeficiency virus infection and salmonellosis in 20-to 59-year-old residents of New York City. *Clin Infect Dis* 1994;18(3):358–363.

3. Keddy KH, Takuva S, Musekiwa A, Puren AJ, Sooka A, Karstaedt A, et al. An association between decreasing incidence of invasive non-typhoidal salmonellosis and increased use of antiretroviral therapy, Gauteng Province, South Africa, 2003–2013. *PLoS One* 2017;12(3):e0173091.

4. Mayanja BN, Todd J, Hughes P, Van der Paal L, Mugisha JO, Atuhumuza E, et al. Septicaemia in a population-based HIV clinical cohort in rural Uganda, 1996-2007: incidence, aetiology, antimicrobial drug resistance and impact of antiretroviral therapy: Septicaemia in a rural Ugandan HIV cohort. *Trop Med Int Health* 2010;15(6):697–705.

5. Muthumbi E, Morpeth SC, Ooko M, Mwanzu A, Mwarumba S, Mturi N, et al. Invasive Salmonellosis in Kilifi, Kenya. *Clin Infect Dis* 2015;61(suppl 4):S290–301.

6. Onchiri FM, Pavlinac PB, Singa BO, *et al.* Low Bacteremia Prevalence Among Febrile Children in Areas of Differing Malaria Transmission in Rural Kenya: A Cross-Sectional Study. *J Pediatric Infect Dis Soc* 2016; 5: 385–94.
